# Supplementary material for: FOXF1 promotes tumor vessel normalization and prevents lung cancer progression through FZD4
Source: EMBO Mol Med. 2024 Apr 8;16(5):3. doi: 10.1038/s44321-024-00064-8 (PMC11099127; doi:10.1038/s44321-024-00064-8)
Supplement: Supplementary file 1 — Appendix [file 44321_2024_64_MOESM1_ESM.pdf]

Appendix

FOXF1 Promotes Tumor Vessel Normalization and Prevents Lung Cancer Progression through FZD4

Table of contents

Appendix Figure S1. Increased proliferation of tumors cells in end*Foxf1*$^{+/-}$ lungs……….1

Appendix Figure S2. Proliferation of end*Foxf1*$^{+/-}$ in LLC lung tumors……………………….3

Appendix Figure S3. Nanoparticle delivery of Fzd4 improved the expression of Claudin-5 and VE-cadherin in TEC……………………………………………………………4

Appendix Table S1. NSCLC patient samples……………………………………...…...6

Appendix Table S2. Antibodies for immunofluorescence……………………..…………7

Appendix Table S3. Primers used in this tudy…………………………………………..…8

Appendix Table S4. Clinical chemistry of the nanoparticle treated and control mice…....9

Appendix Table S5. Hematology indices of the nanoparticle treated and control mice..10

Appendix Figure S1

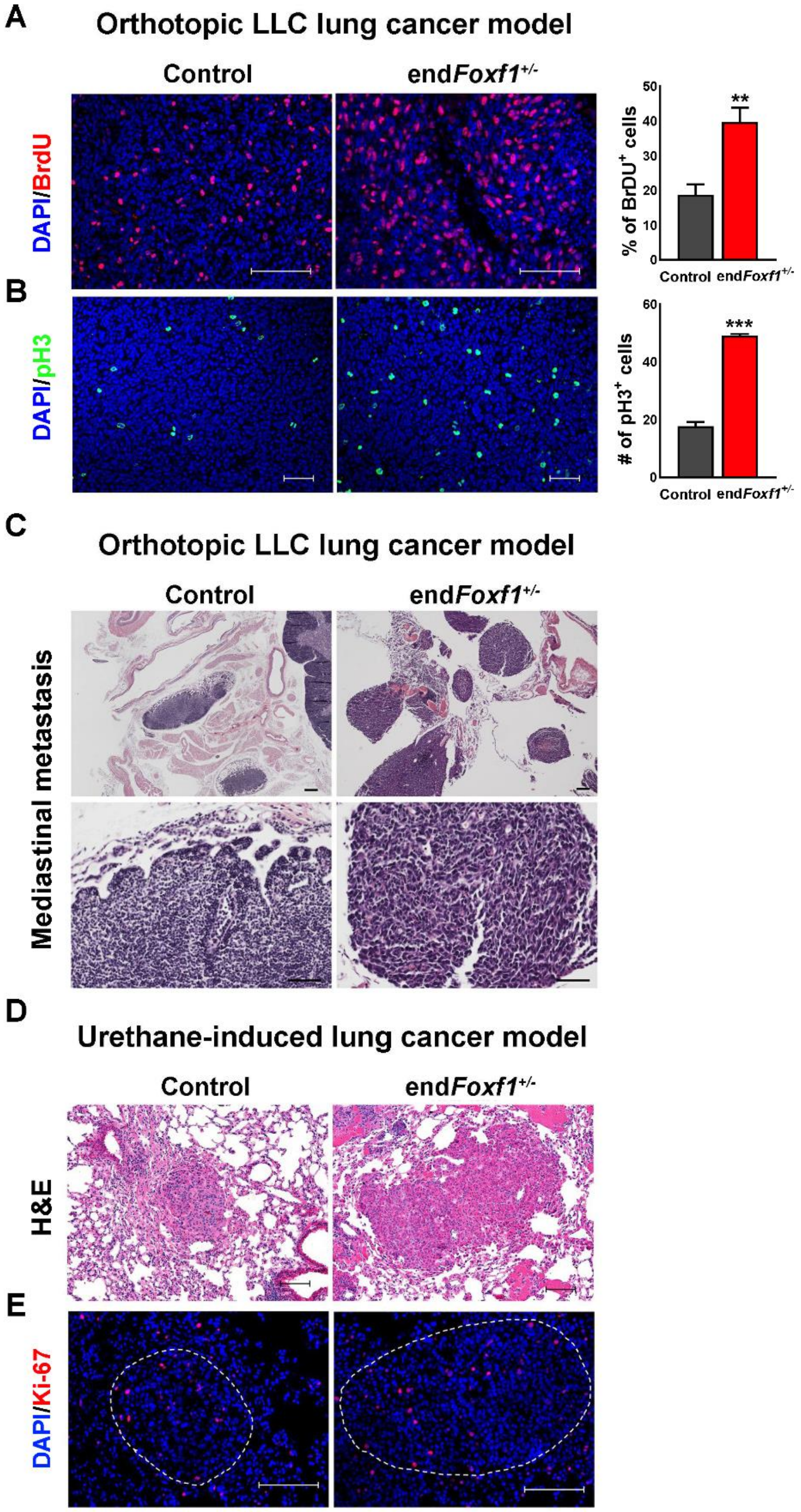

Appendix Figure S1. Increased proliferation of tumors cells in end*Foxf1*$^{+/-}$ lungs. (A) Depletion of *Foxf1* in endothelial cells (end*Foxf1*$^{+/-}$ mice) increases proliferation of tumors cells in orthotopic LLC mouse models of lung cancer, shown by immunostaining for BrDU (red). Nuclei are counterstained with Hoechst 33342. Number of BrDU^{+} cells are counted in 5 random fields and presented as mean ± SEM. (N = 4-6 mice per group). $^{**}P = 0.0067$. **(B)** Immunostaining shows increased number of pH3-positive (green) cells within LLC tumors of end*Foxf1*$^{+/-}$ lungs compared to controls. Nuclei were counterstained with Hoechst 33342. Number of pH3^{+} cells were counted in 5 random fields and presented as mean ± SEM. (N = 3 mice per group). $^{***}P < 0.001$. **(C)** H&E stained images of mediastinal lymph node metastases found in control and endFoxf1+/- mice. (N = 8-11 mice per group). **(D)** Depletion of *Foxf1* in endothelial cells increases size of lung tumors in urethane-induced mouse model of lung cancer, shown using H&E staining. **(E)** Higher proliferation of tumor cells in urethane-treated end*Foxf1*$^{+/-}$ mice is shown by increased number of Ki-67^{+} cells (red) as compared to control mice. Scale bars: 100μm. Data information: Data represent different numbers (N) of biological replicates. The data with error bars are shown as mean ± SEM. Statistical analysis was performed using the two-tailed unpaired-sample Student *t* test. Source data are available online for this figure.

Appendix Figure S2

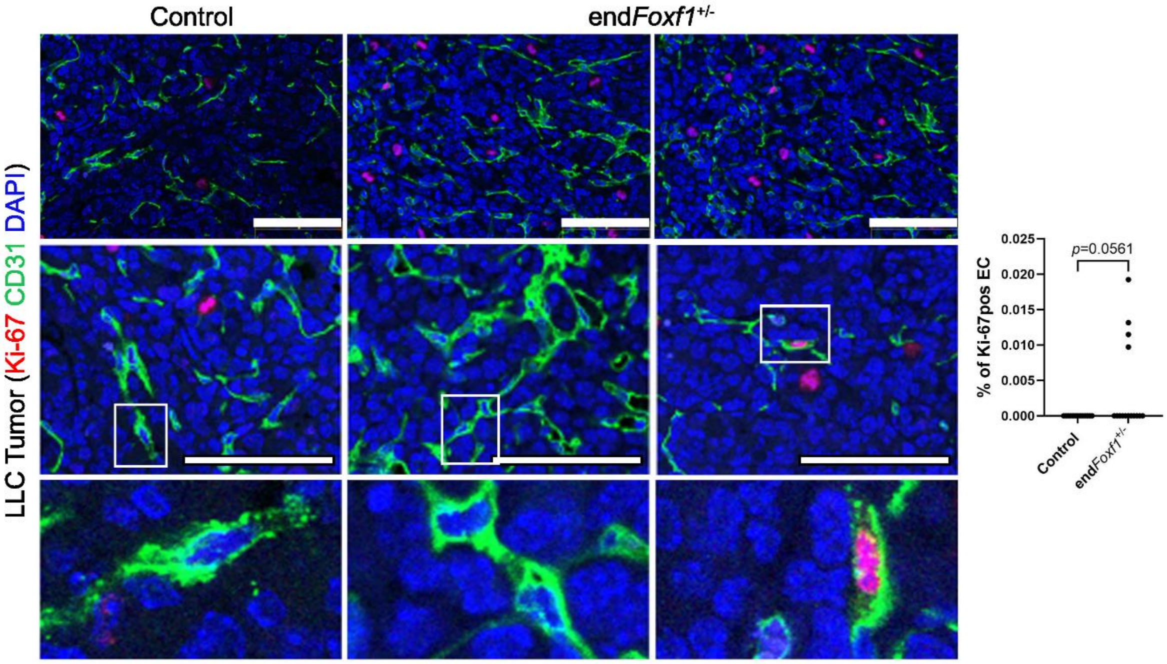

Appendix Figure S2. Proliferation of end*Foxf1*$^{+/-}$ in LLC lung tumors. Immunostaining shows no significant differences in the percentage of Ki-67-positive (red) endothelial cells (green) within LLC tumors of end*Foxf1*$^{+/-}$ lungs compared to controls. Nuclei were counterstained with DAPI. (N = 13-15 mice per group). Scale bar = 75μm. Data information: Data represent different numbers (N) of biological replicates.

Appendix Figure S3

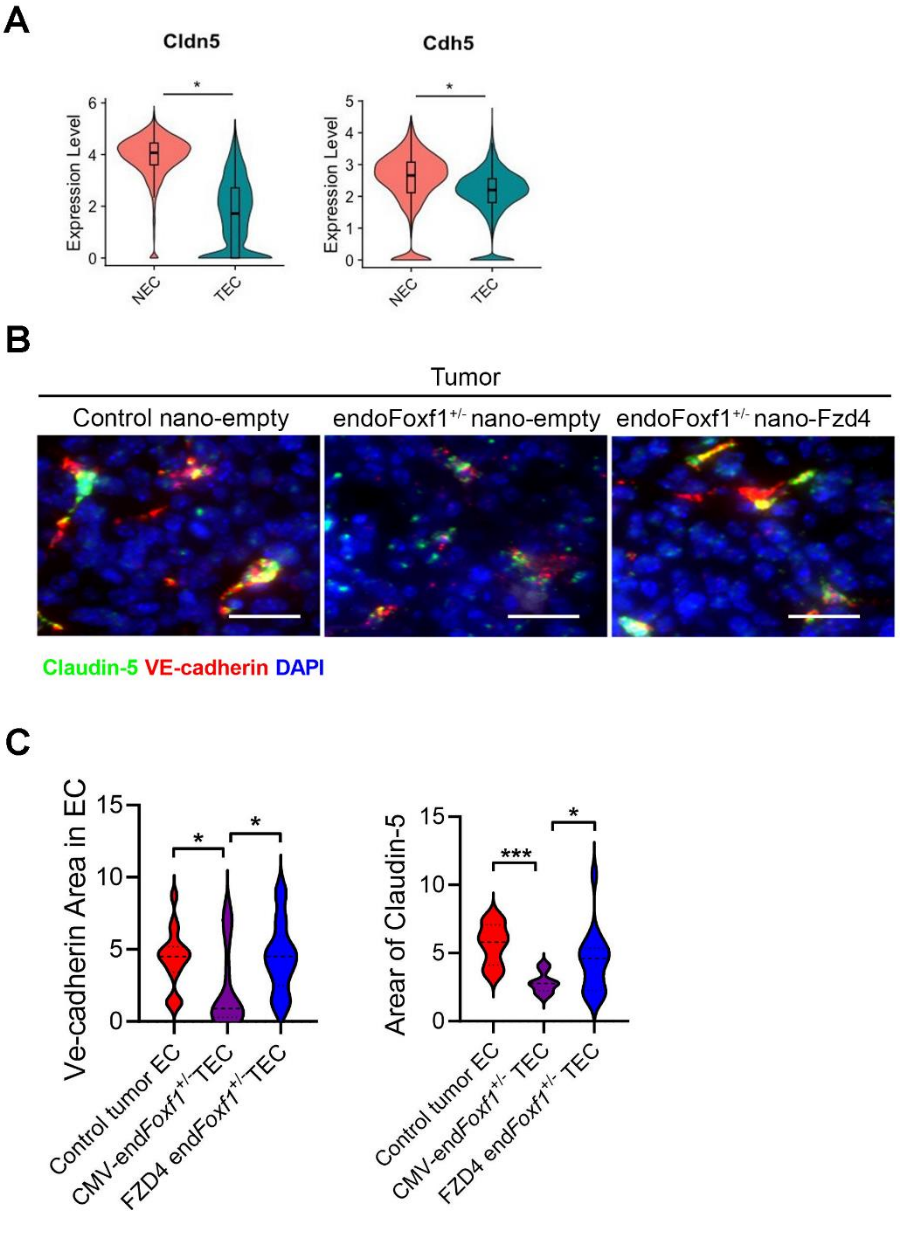

Appendix Figure S3. Nanoparticle delivery of *Fzd4* improved the expression of Claudin-5 and VE-cadherin in TEC. (A) Decreased *Claudin-5* and *Cdh5* mRNA levels were found in TECs compared to NECs. scRNAseq analysis was done using lung endothelial cells from scRNA-seq dataset (Goveia *et al.*, 2020). Boxplots show median,

Q1 and Q3 quartiles and whiskers up to 1.5× interquartile range. **(B)** RNAscope results showed decreased Claudin-5 (green) and VE-cadherin (red) transcripts in end*Foxf1*$^{+/-}$ TECs compared to control TECs (left and middle panels)**.** Treatment with nano-Fzd4 increased Claudin-5 and VE-cadherin in end*Foxf1*$^{+/-}$ TECs (right panel). Nuclei were counterstained with DAPI (blue). Bar = 20μm. **(C)** Claudin-5 and VE-cadherin positive cells were counted in 5-10 random fields per tumor and presented as mean ± SD (n= 4-5 samples per group). Relative *Claudin-5* and *VE-cadherin* intensity were quantified using Nikon NIE CIC Analysis Elements software. Data information: Data represent different numbers (N) of biological replicates. The data with error bars are shown as mean ± SEM. *$P < 0.05$, ***$P < 0.001$, as determined using the Wilcoxon rank-sum test (**A**), or One-way ANOVA followed by Tukey's post hoc test (**C**). Source data are available online for this figure.

Appendix Table S1. NSCLC patient samples

Tissue of origin	Case Diagnosis	Tumor grade	TNM
Lung	Adenocarcinoma	G2	pT2b N0 Mx
Lung	Adenocarcinoma	G2	pT1c N0 Mx
Lung	Adenocarcinoma	G2	pT2a N0 Mx
Lung	Adenocarcinoma	G3	pT1b N0 Mx
Lung	Adenocarcinoma	G2	pT2a N2 Mx
Lung	Adenocarcinoma	G2	pT1b N0 MX
Lung	Adenocarcinoma	G1	pT1b N0 MX
Lung	Adenocarcinoma	G2	pT2a N0 MX
Lung	Adenocarcinoma	G3	pT2b N0 MX
Lung	Adenocarcinoma	G1	pT1b N0 Mx
Lung	Adenocarcinoma	G2	pT1a Nx Mx
Lung	Squamous Cell Carcinoma	G3	pT3 N0 Mx
Lung	Squamous Cell Carcinoma	G2	pT2a N1 Mx
Lung	Squamous Cell Carcinoma	G2	pT1c N0 Mx
Lung	Squamous Cell Carcinoma	G2	pT1a N0 MX
Lung	Squamous Cell Carcinoma	G1	pT2a N0 MX
Lung	Squamous Cell Carcinoma	G3	pT2b N0 MX
Lung	Squamous Cell Carcinoma	G3	pT3 N1 MX

Appendix Table S2. Antibodies for immunofluorescence

Antibody	Supplier	Catalog #	Dilution
FOXF1	RnD Systems	AF4798	1:100
CD31	Abcam	AB28364	1:200
CD31	RnD Systems	AF3628	1:200
SOX17	Seven Hills	GP951	1:200
NG2	Millipore	AB5320	1:100
COL IV	Abcam	AB19808	1:100
HP-1	HPI	HP1-Mab1	1:50
CD8	Thermo	PA5-81344	1:200
β-CATENIN	Cell Signaling Technology	CST9582	1:100
FZD1	R&D	MAB11201	1:100
BrDU	DSHB	AB_2314035	1:50
pH3	Santa Cruz	SC374669	1:200
Ki-67	Abcam	AB16667	1:200

Appendix Table S3. Primers used in this study

**Mouse Taqman Primers for qRT-PCR**	
Actb	Mm00607939_s1
Foxf1	Mm00487497_m1
Axin2	Mm01265783_m1
Fzd4	Mm00433382_m1
Axin1	Mm01299062_g1
Ccnd1	Mm00432359_m1
Pecam1	Mm01242576_m1
Lef1	Mm00550265_m1
**Human Taqman Primers for qRT-PCR**	
FOXF1	Hs00230962_m1
ACTB	Hs99999903_m1
FZD1	Hs00268943_s1
AXIN1	Hs00394718_m1
AXIN2	Hs00610344_m1
CCND1	Hs00277039_m1
LEF1	Hs00212390_m1
**Genotyping primers for *TetO7-HA-mFoxf1*$^{tg/+}$**	
Forward	5'- TTG GCT GGC AAC GTG GAC G -3'
Reverse	5'- TCA CAT CAC ACA CGG CTT GAT G -3'

Appendix Table S4. Clinical chemistry of the nanoparticle treated and control mice

**Liver Function**	Unit	Control	Nanoparticle	*P* Value	Significance
Total Protein	g/dL	4.77 ± 0.26	5.03 ±0.09	0.24769	ns
Albumin	g/dL	2.37 ± 0.04	2.40 ±0.08	0.64333	ns
Globulins	g/dL	2.40 ± 0.22	2.63 ±0.05	0.20988	ns
ALP	U/L	87.00 ± 4.55	85.67 ±6.55	0.82464	ns
Total Bilirubin	mg/dL	0.23 ± 0.05	0.17 ±0.05	0.2302	ns
GGT	U/L	2.00 ± 0.812	2.00 ±0.00	1	ns
ALT	U/L	33.33 ± 6.94	27.00 ±2.16	0.28556	ns
**Kidney Function**					
Creatinine	mg/dL	0.10 ± 1.00	0.10 ± 1.00	1	ns
BUN	mg/dL	21.47 ±0.54	20.5 ± 1.03	0.32165	ns
**Other**					
Glucose	mg/dL	238.33 ± 5.25	239.33 ± 7.26	0.91414	ns
Phosphorus	mg/dL	7.63 ± 0.33	7.83 ± 0.34	0.58264	ns
Cholesterol	mg/dL	163.00 ± 14.76	201.67 ± 12.12	0.07593	ns
Calcium	mg/dL	10.10 ± 0.08	9.97 ± 0.09	0.20511	ns

Appendix Table S5. Hematology indices of the nanoparticle treated and control mice

**Leukocytes**	Unit	Control	Nanoparticle	*P* Value	Significance
WBC	K/µL	2.79 ± 0.39	2.37 ± 0.41	0.53527993	ns
Neutrophil	K/µL	0.52 ± 0.24	0.45 ± 0.03	0.79500241	ns
Lymphocytes	K/µL	2.01 ± 0.02	1.66 ± 0.31	0.37688478	ns
Monocytes	K/µL	0.17 ± 0.06	0.23 ± 0.06	0.5527864	ns
Eosinophils	K/µL	0.08 ± 0.06	0.03 ± 0.01	0.49748109	ns
Basophils	K/µL	0.015 ± 0.015	0.005 ± 0.005	0.59175171	ns
Neutrophil	%	17.72 ± 6.02	19.19 ± 2.36	0.84064552	ns
Lymphocytes	%	73.40 ± 9.47	69.91 ± 0.95	0.74900977	ns
Monocytes	%	5.76 ± 1.38	9.58 ± 1.02	0.15494377	ns
Eosinophils	%	2.65 ± 1.71	1.16 ± 0.30	0.48119513	ns
Basophils	%	0.48 ± 0.38	0.16 ± 0.09	0.49985822	ns
**Erythrocytes**					
RBC	M/µL	8.70 ± 0.48	8.71± 0.36	0.98807664	ns
Hb	g/dL	13.30 ± 0.80	12.55 ± 0.75	0.56462258	ns
HCT	%	64.50 ± 3.40	62.35 ± 4.35	0.73452238	ns
MCV	fL	74.15 ± 0.15	71.55 ± 2.05	0.33333333	ns
MCH	pg	15.30 ± 0.10	14.40 ± 0.30	0.10446653	ns
MCHC	g/dL	20.65 ± 0.15	20.10 ± 0.20	0.15880898	ns
RDW	%	17.60 ± 0.20	18.55 ± 0.15	0.06279912	ns
**Thrombocytes**					
PLT	K/µL	608.50 ± 52.50	999.50 ± 314.50	0.34487939	ns
MPV	fL	6.05 ± 0.45	6.60 ± 0.20	0.3802202	ns

1
