# Supplementary figures and images for: FOXF1 promotes tumor vessel normalization and prevents lung cancer progression through FZD4
Source: EMBO Mol Med. 2024 Apr 8;16(5):3. doi: 10.1038/s44321-024-00064-8 (PMC11099127; doi:10.1038/s44321-024-00064-8)

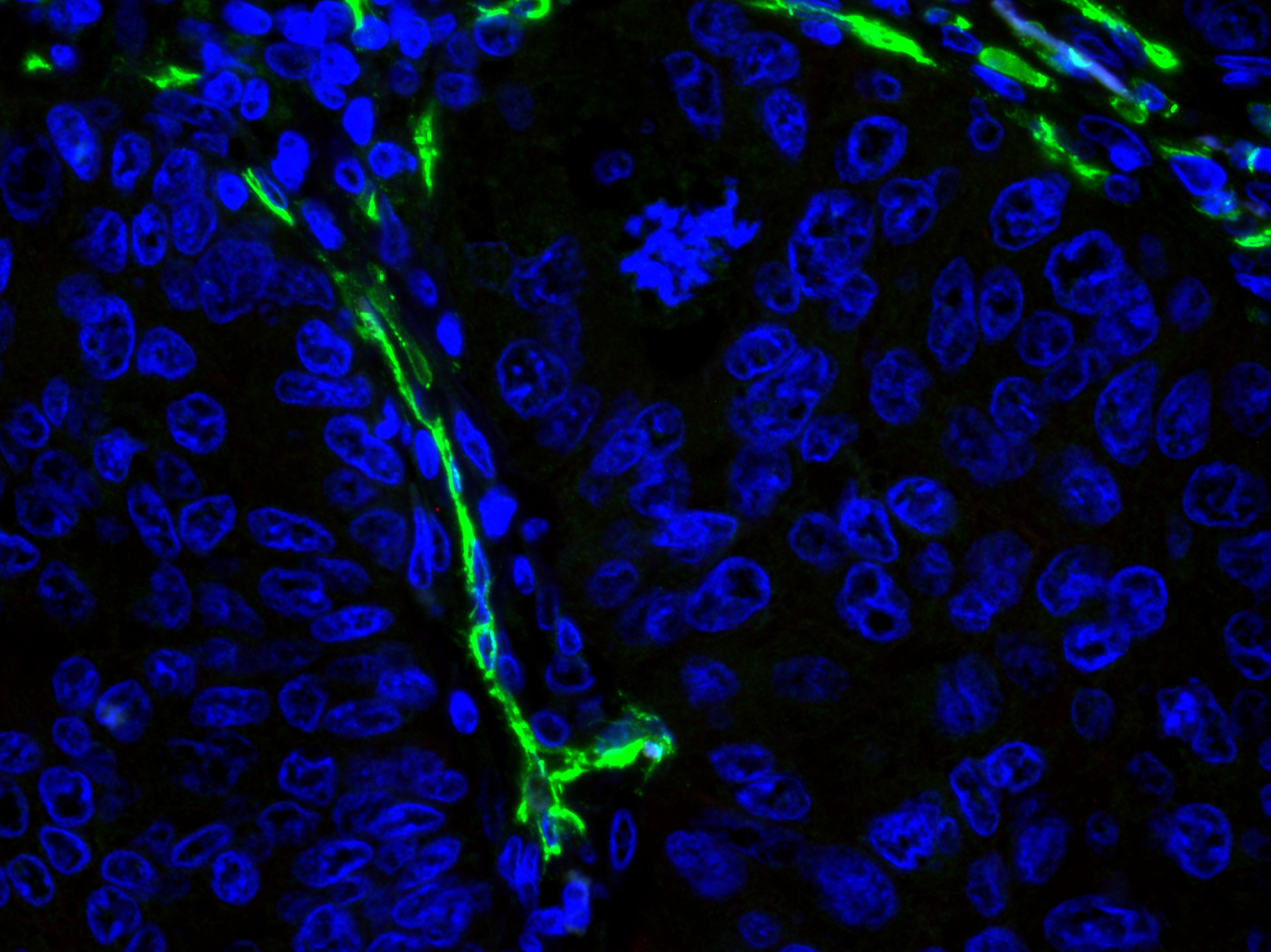

Supplement: Supplementary file 2 — Source data Fig. 1 [file 44321_2024_64_MOESM2_ESM.zip › Figure 1/Figure 1A/Adenocarcinoma/MC08-1wH ad 6b 40X-1_(c2+c3+c4).TIF]

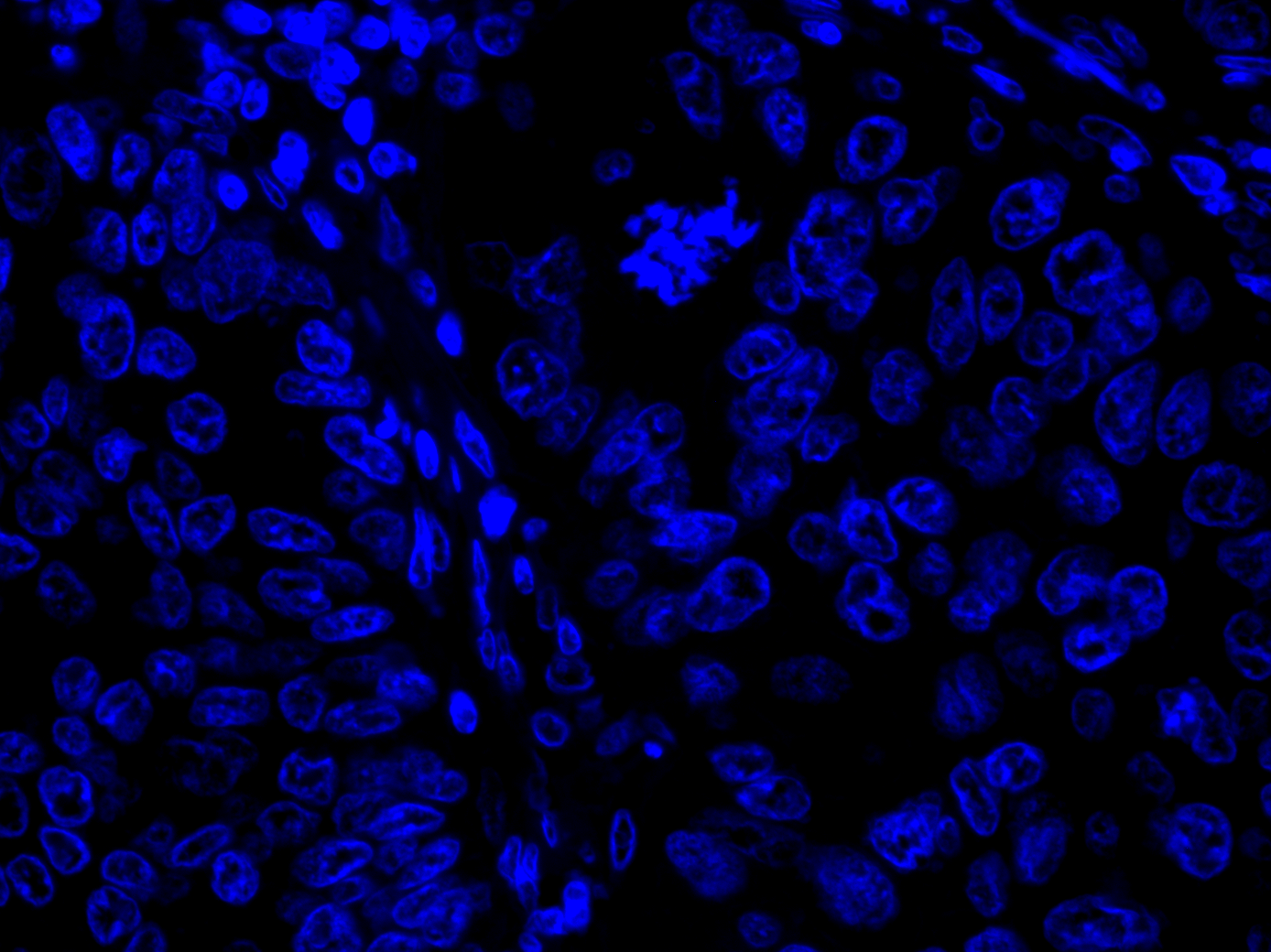

Supplement: Supplementary file 2 — Source data Fig. 1 [file 44321_2024_64_MOESM2_ESM.zip › Figure 1/Figure 1A/Adenocarcinoma/MC08-1wH ad 6b 40X-1_c2.TIF]

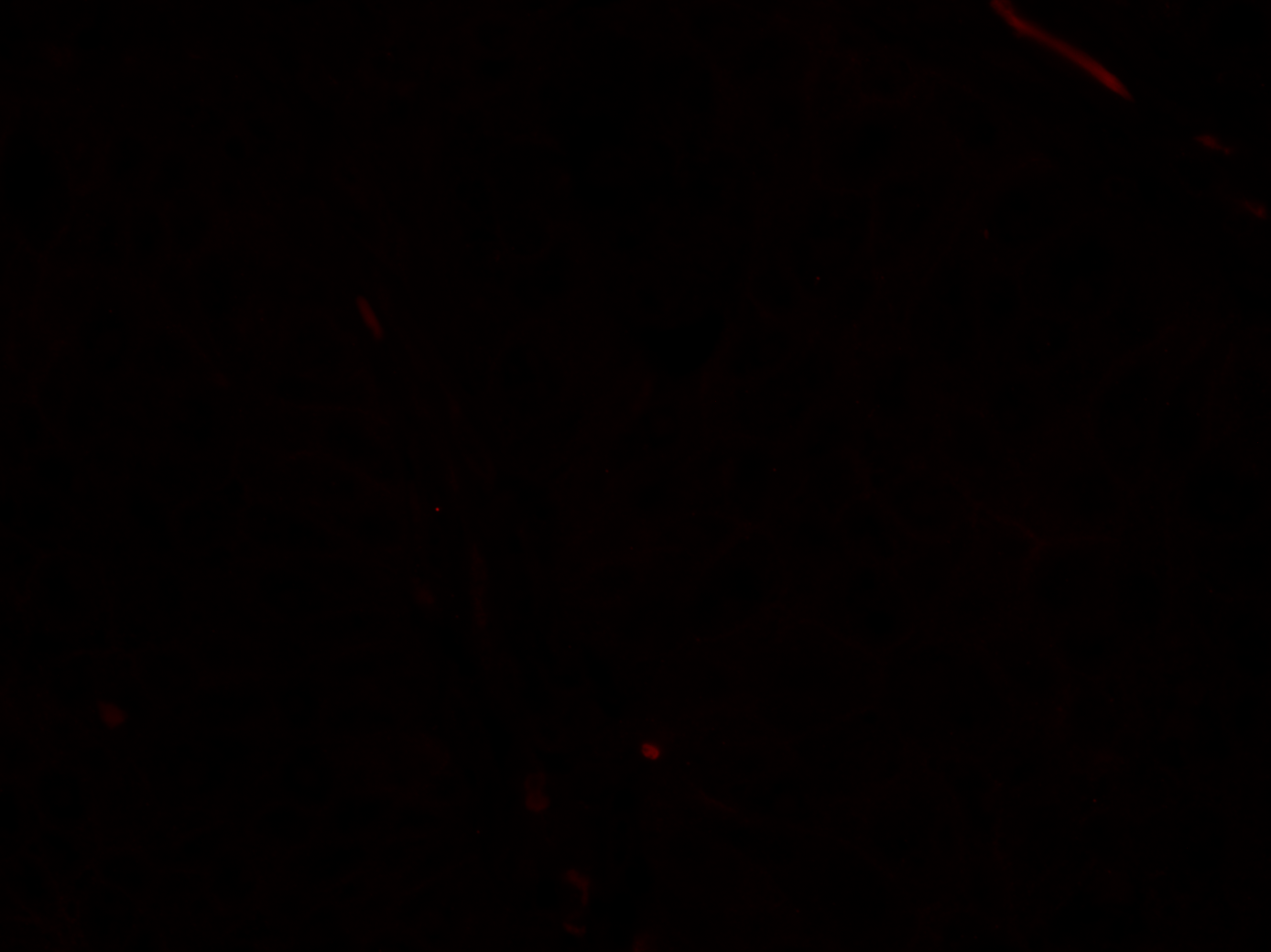

Supplement: Supplementary file 2 — Source data Fig. 1 [file 44321_2024_64_MOESM2_ESM.zip › Figure 1/Figure 1A/Adenocarcinoma/MC08-1wH ad 6b 40X-1_c3.TIF]

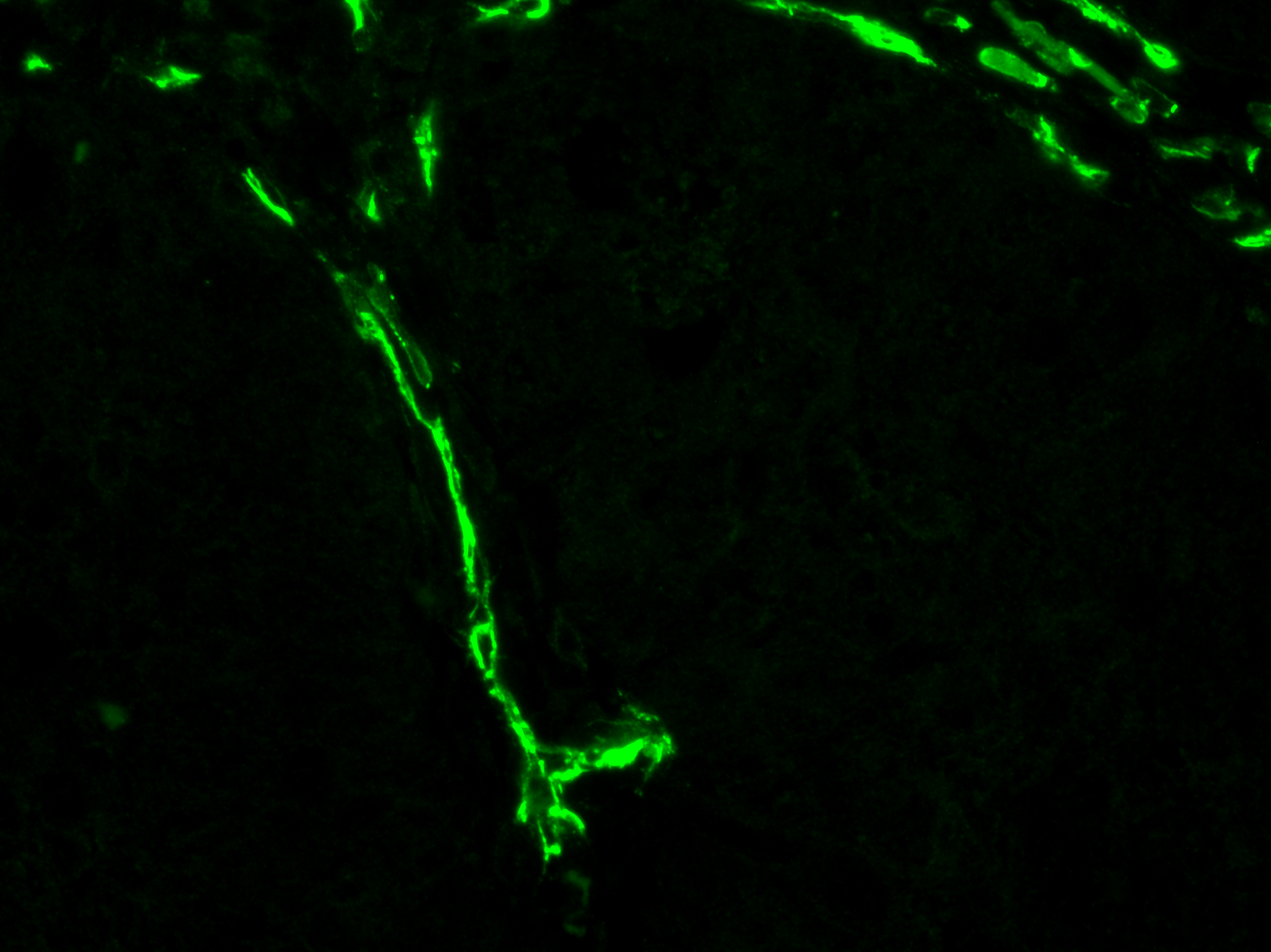

Supplement: Supplementary file 2 — Source data Fig. 1 [file 44321_2024_64_MOESM2_ESM.zip › Figure 1/Figure 1A/Adenocarcinoma/MC08-1wH ad 6b 40X-1_c4.TIF]

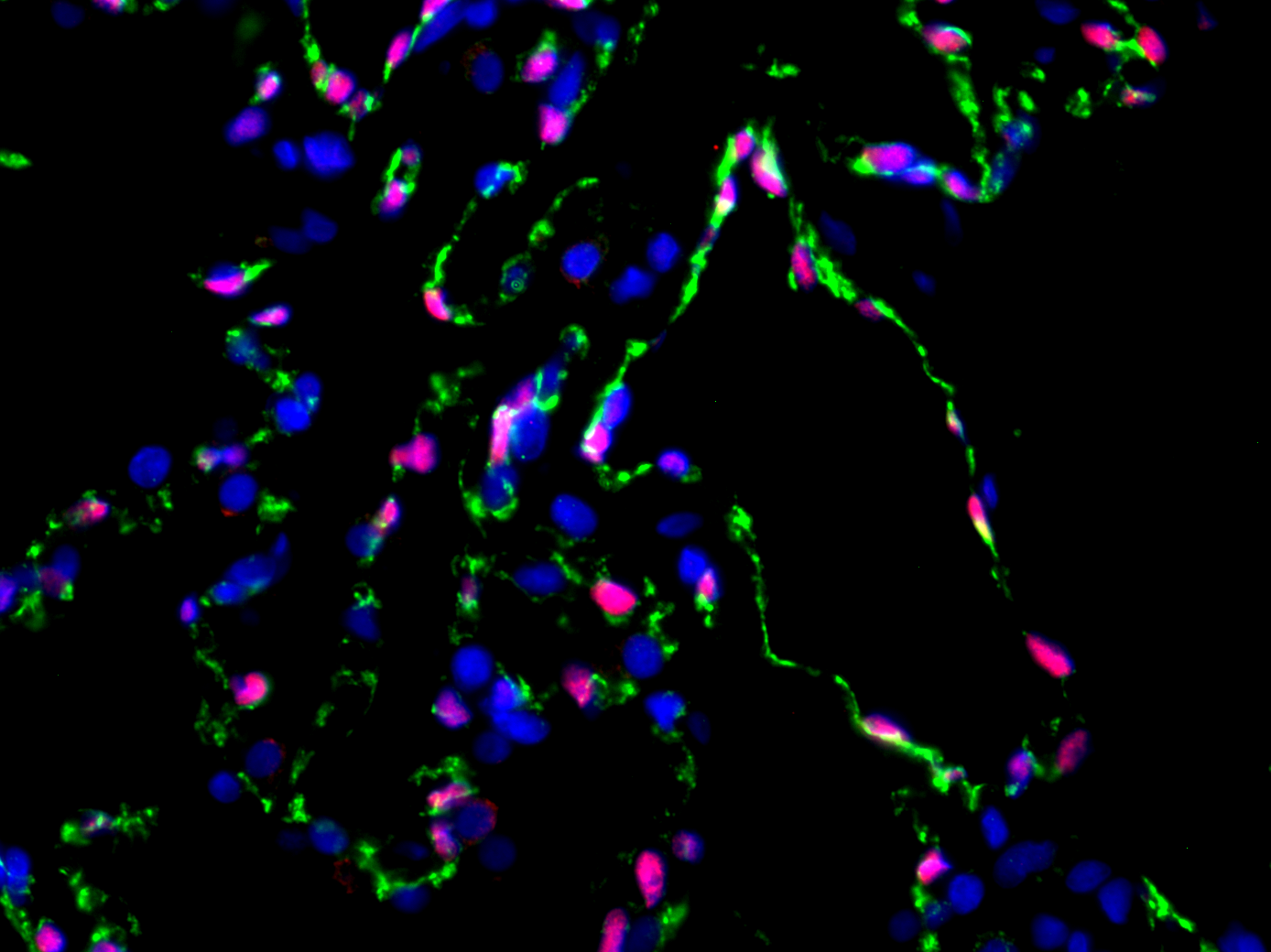

Supplement: Supplementary file 2 — Source data Fig. 1 [file 44321_2024_64_MOESM2_ESM.zip › Figure 1/Figure 1A/Donor Lung bottom panel/MC15-3H 40X-14_(c2+c4+c5).TIF]

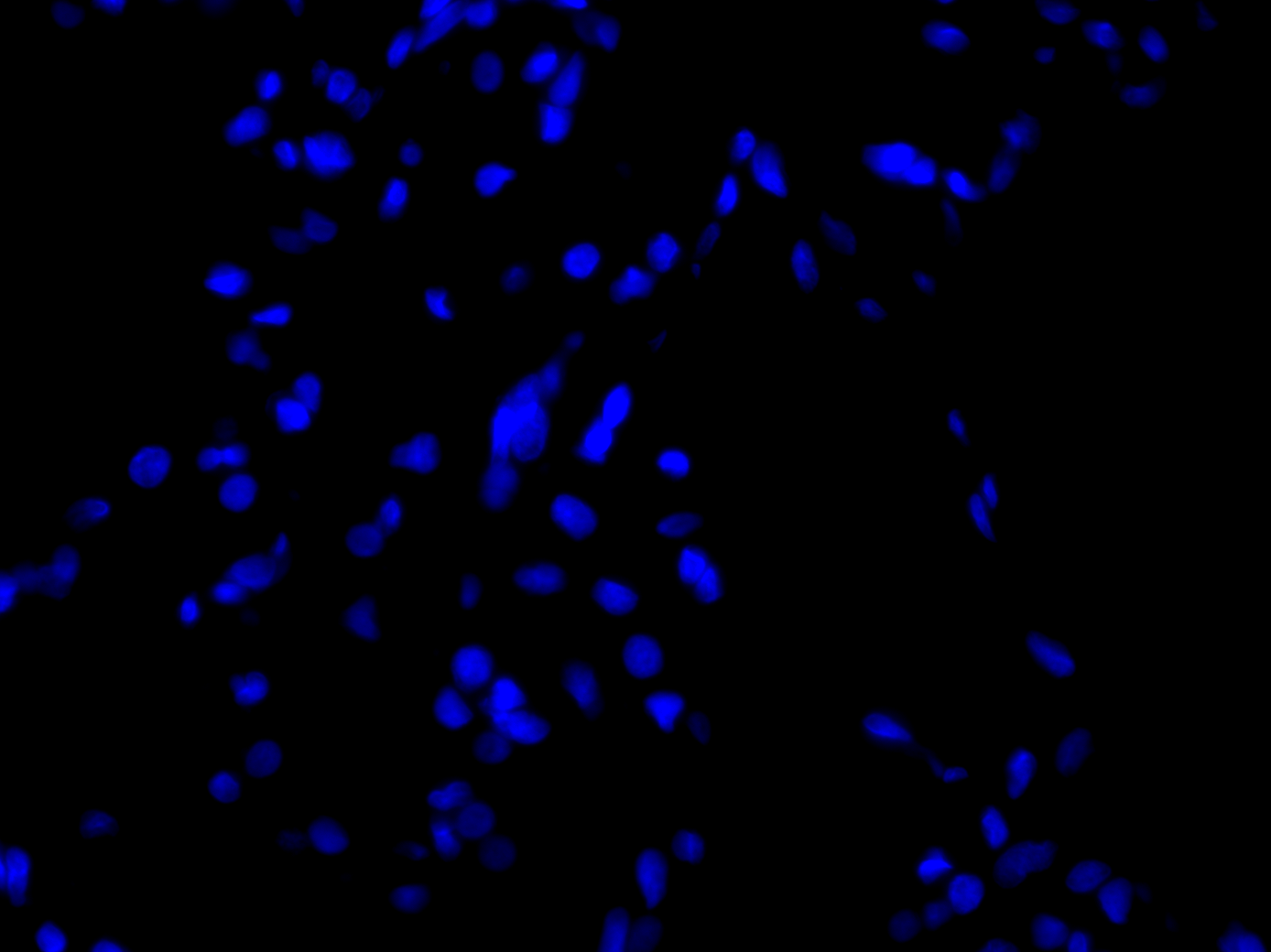

Supplement: Supplementary file 2 — Source data Fig. 1 [file 44321_2024_64_MOESM2_ESM.zip › Figure 1/Figure 1A/Donor Lung bottom panel/MC15-3H 40X-14_c2.TIF]

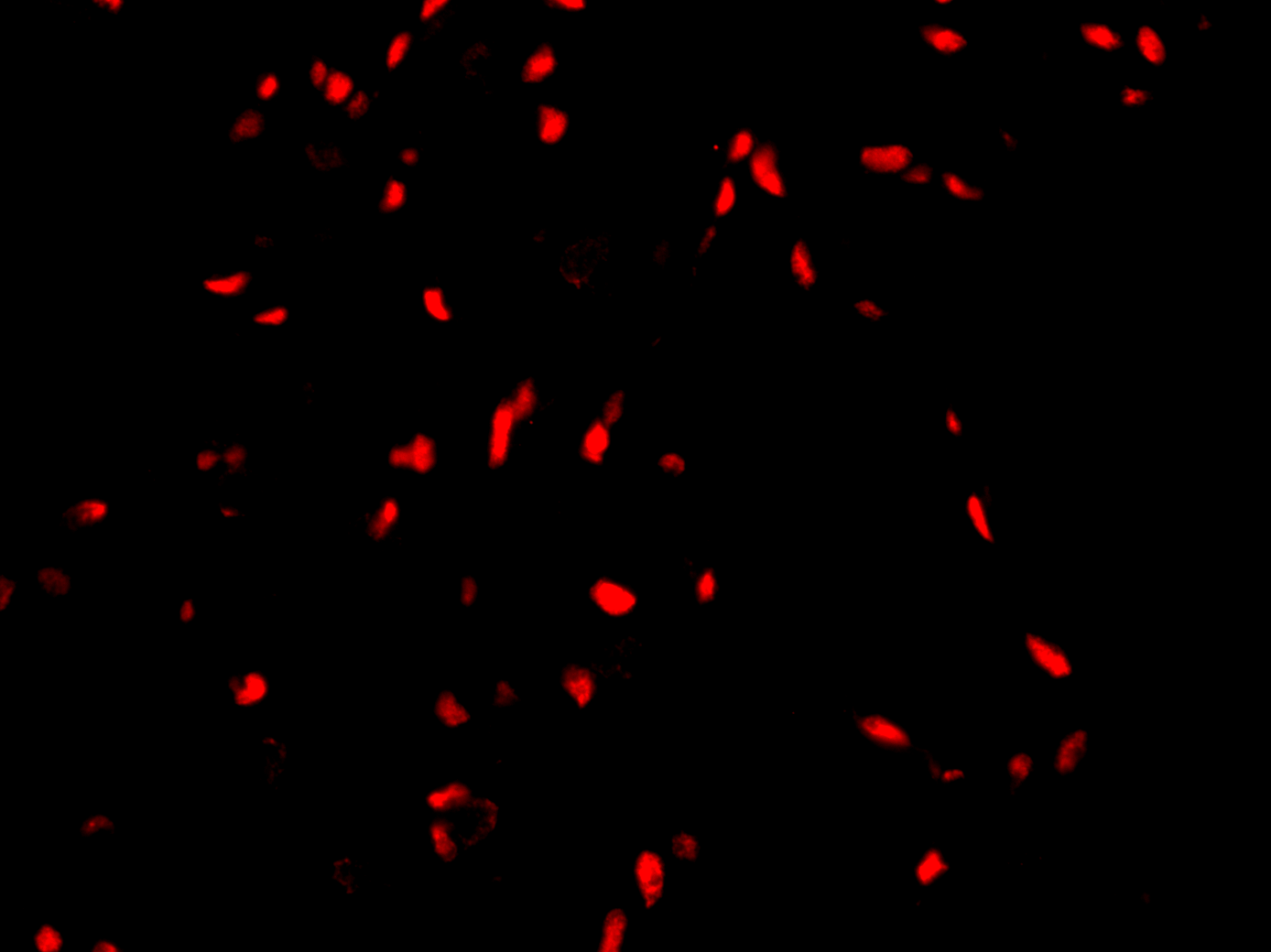

Supplement: Supplementary file 2 — Source data Fig. 1 [file 44321_2024_64_MOESM2_ESM.zip › Figure 1/Figure 1A/Donor Lung bottom panel/MC15-3H 40X-14_c4.TIF]

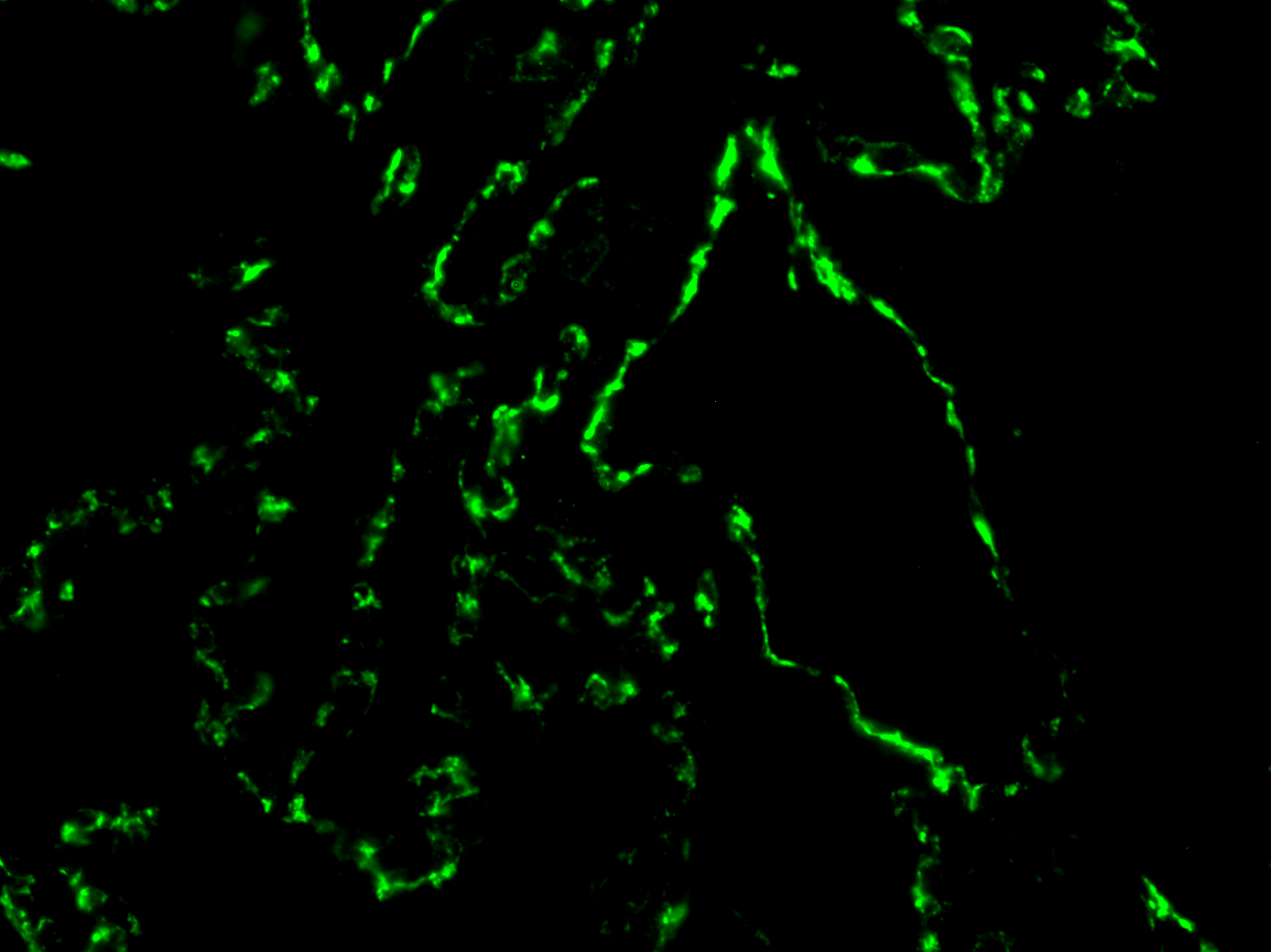

Supplement: Supplementary file 2 — Source data Fig. 1 [file 44321_2024_64_MOESM2_ESM.zip › Figure 1/Figure 1A/Donor Lung bottom panel/MC15-3H 40X-14_c5.TIF]

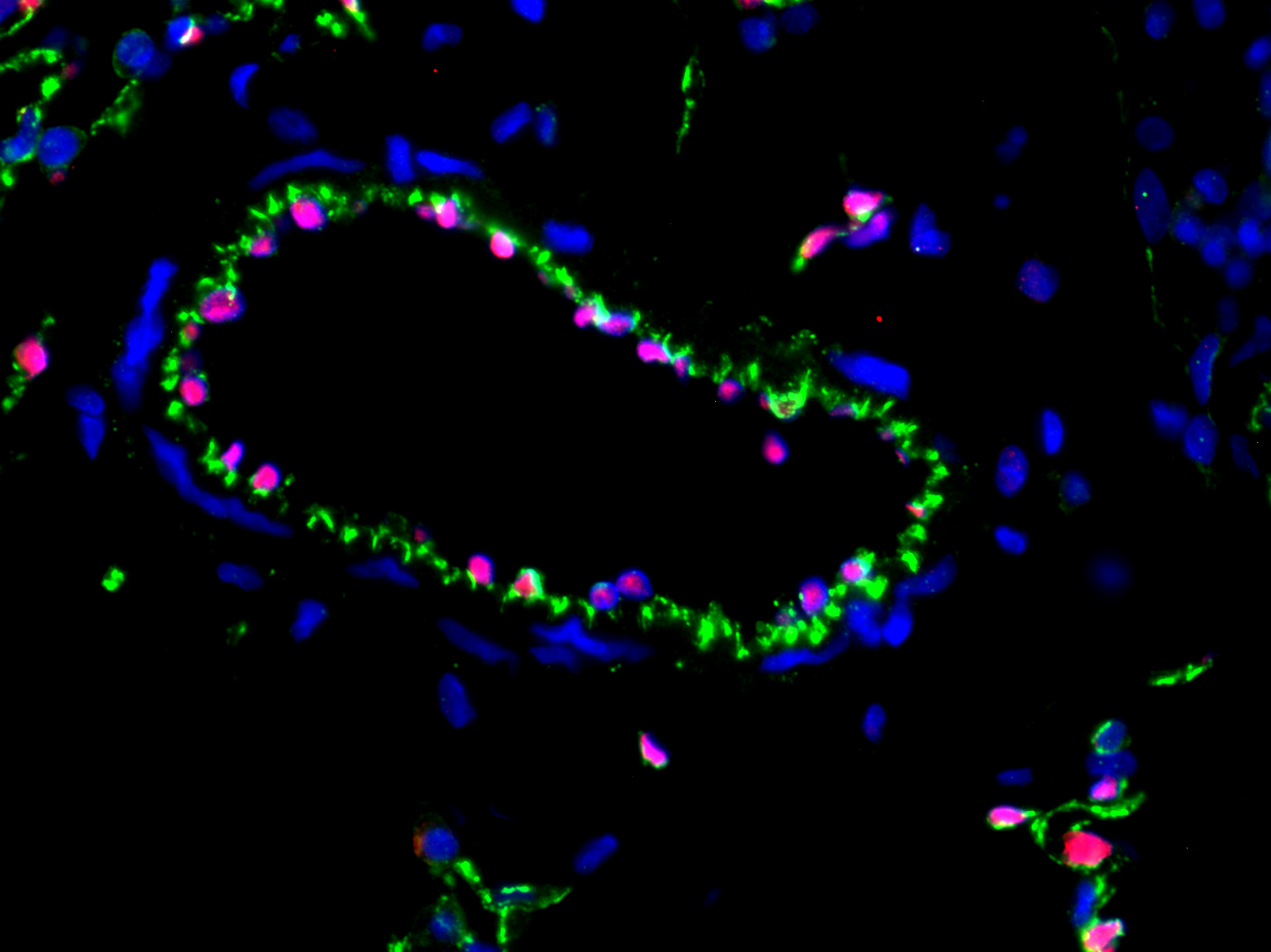

Supplement: Supplementary file 2 — Source data Fig. 1 [file 44321_2024_64_MOESM2_ESM.zip › Figure 1/Figure 1A/Donor lung top panel/MC15-3H 40X-15_(c2+c4+c5).TIF]

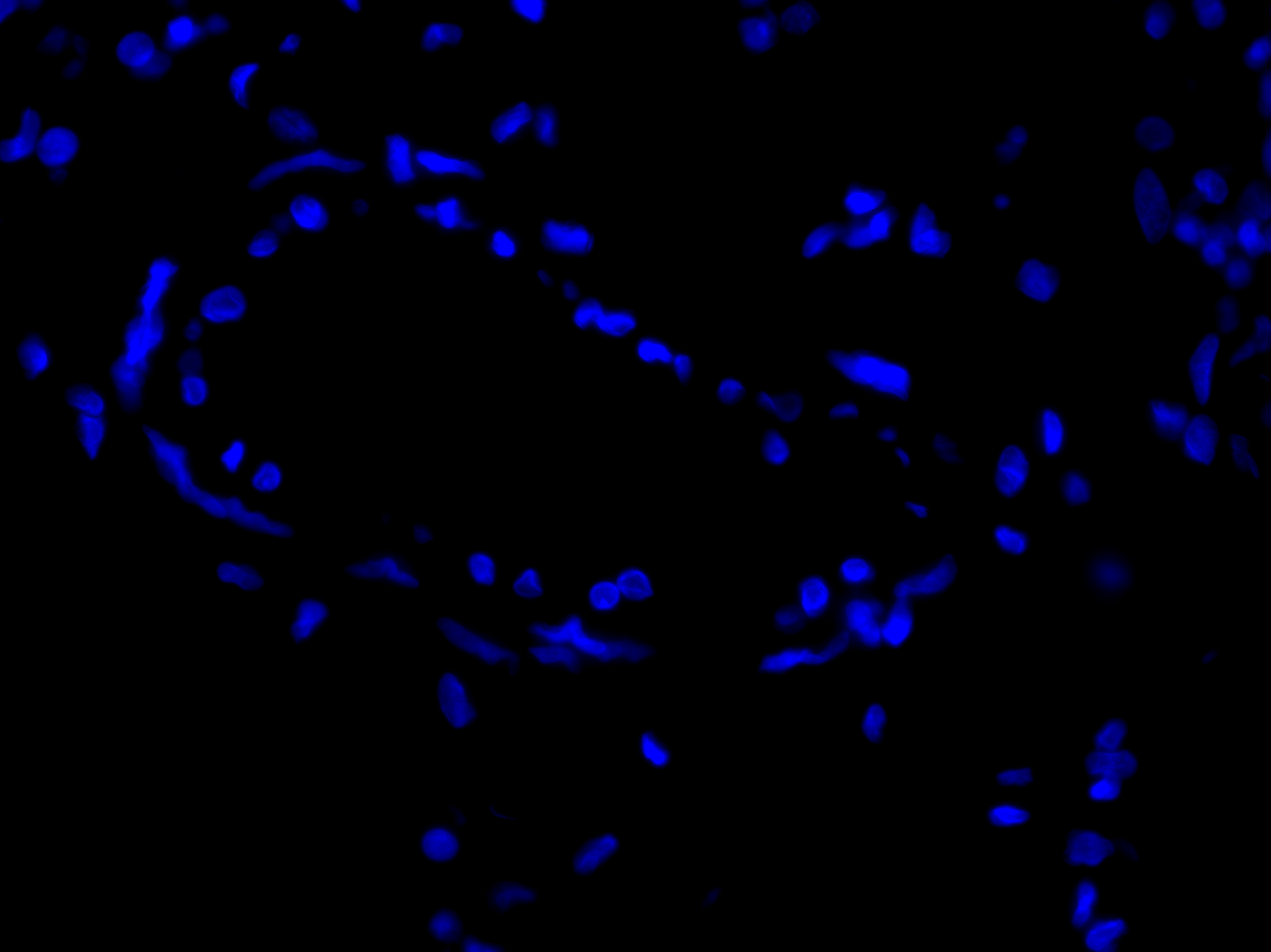

Supplement: Supplementary file 2 — Source data Fig. 1 [file 44321_2024_64_MOESM2_ESM.zip › Figure 1/Figure 1A/Donor lung top panel/MC15-3H 40X-15_c2.TIF]

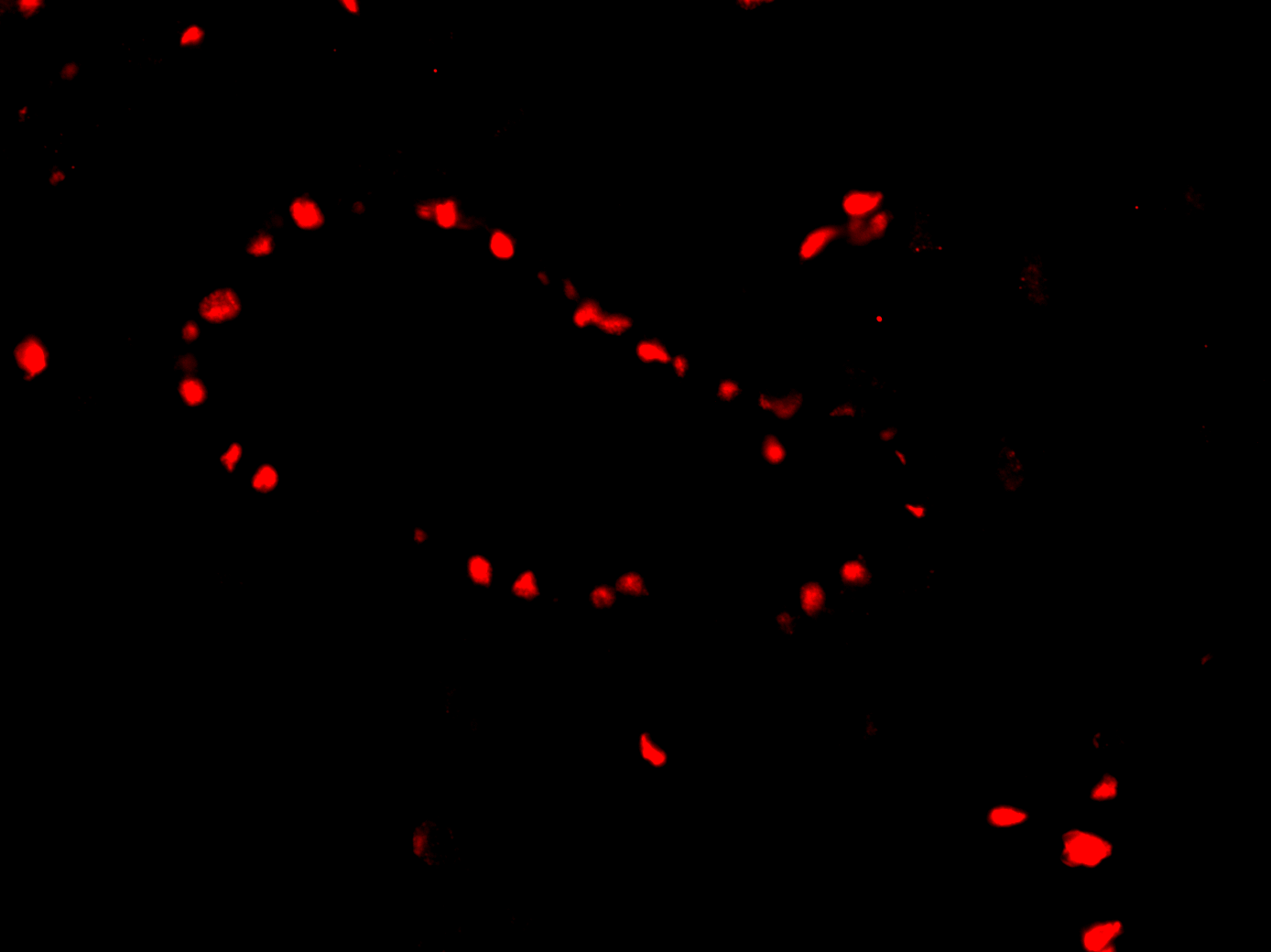

Supplement: Supplementary file 2 — Source data Fig. 1 [file 44321_2024_64_MOESM2_ESM.zip › Figure 1/Figure 1A/Donor lung top panel/MC15-3H 40X-15_c4.TIF]

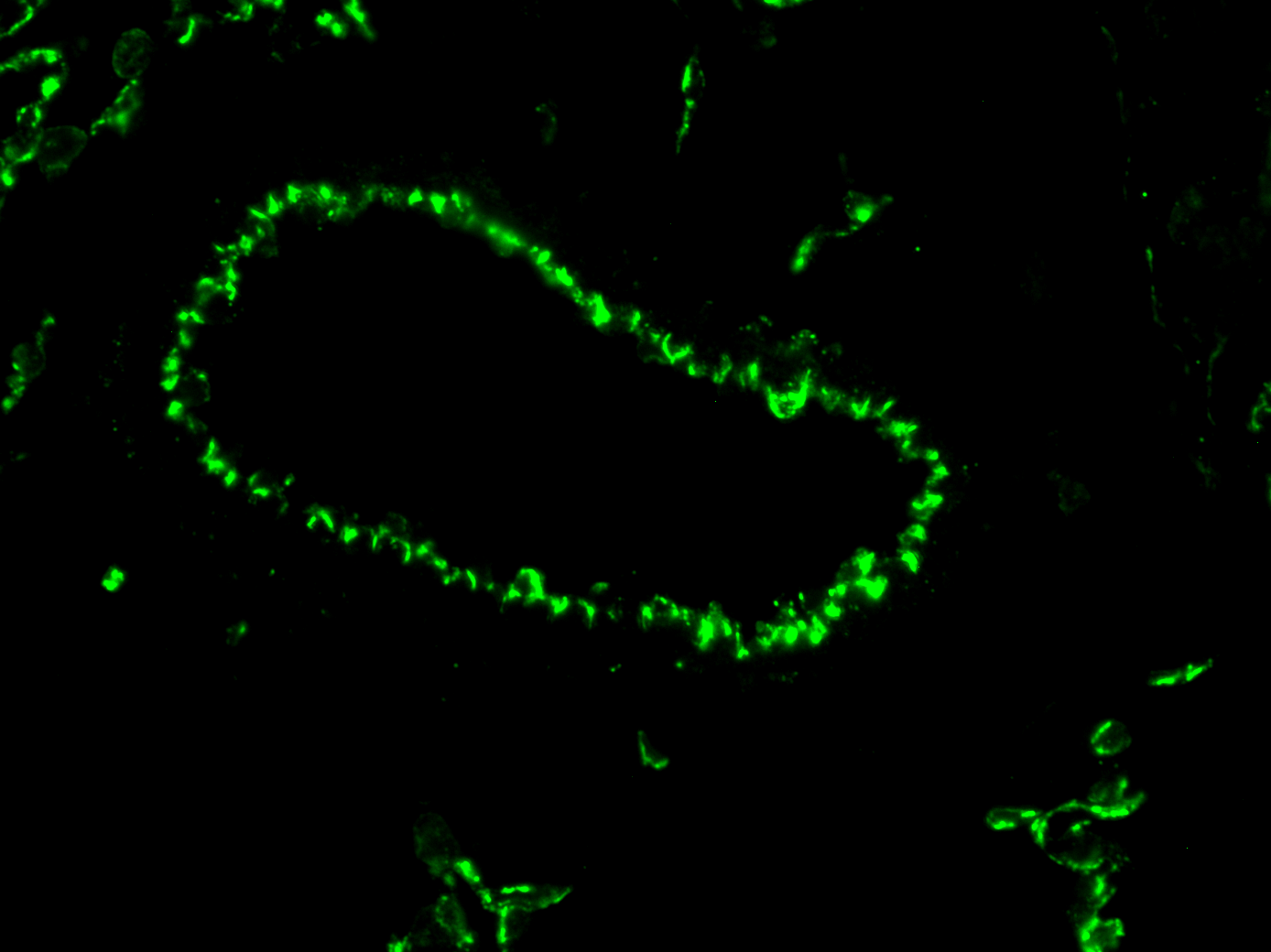

Supplement: Supplementary file 2 — Source data Fig. 1 [file 44321_2024_64_MOESM2_ESM.zip › Figure 1/Figure 1A/Donor lung top panel/MC15-3H 40X-15_c5.TIF]

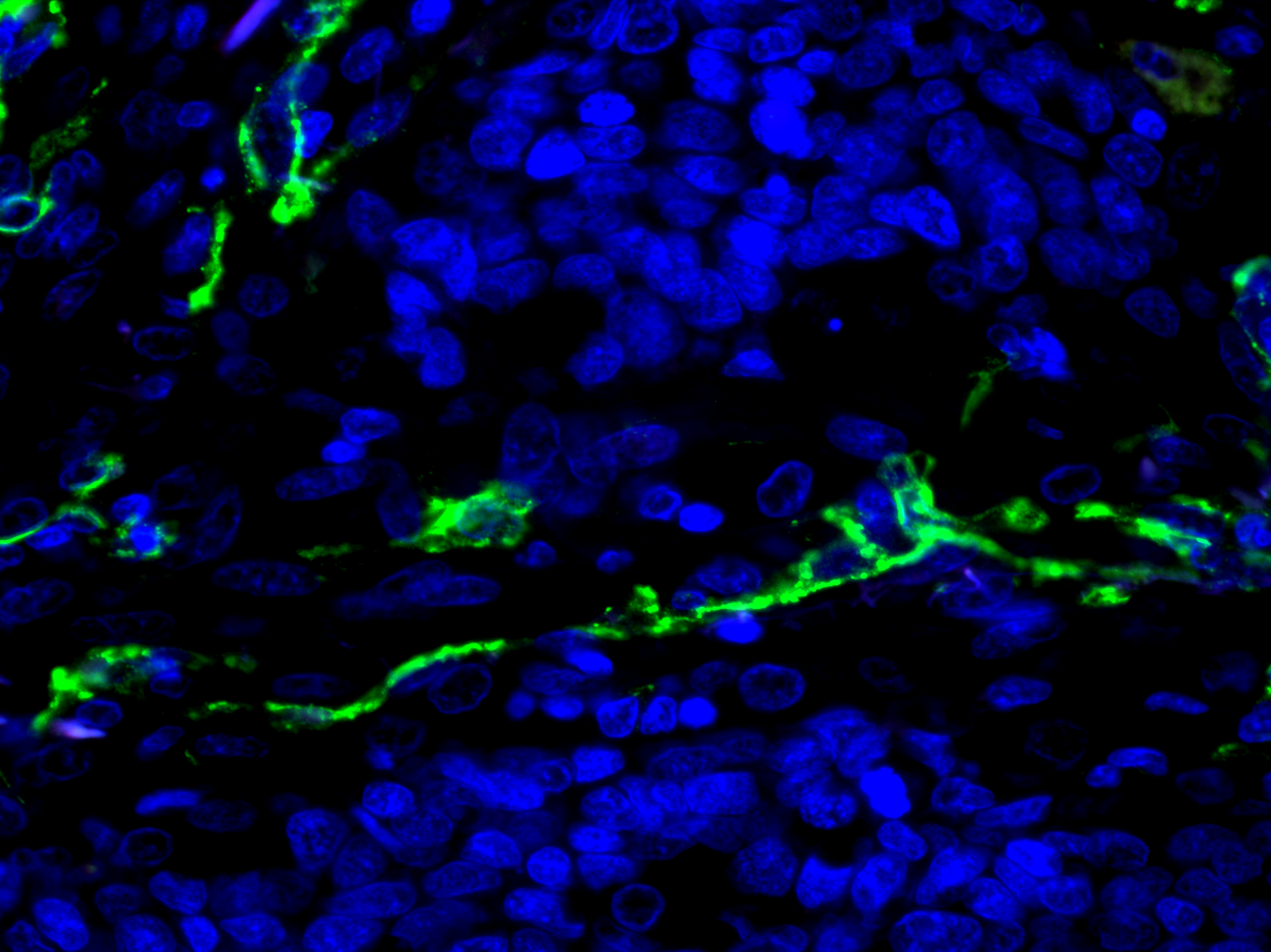

Supplement: Supplementary file 2 — Source data Fig. 1 [file 44321_2024_64_MOESM2_ESM.zip › Figure 1/Figure 1A/SCC/MC08-2wH sq 12b 40X-1_(c2+c3+c4).TIF]

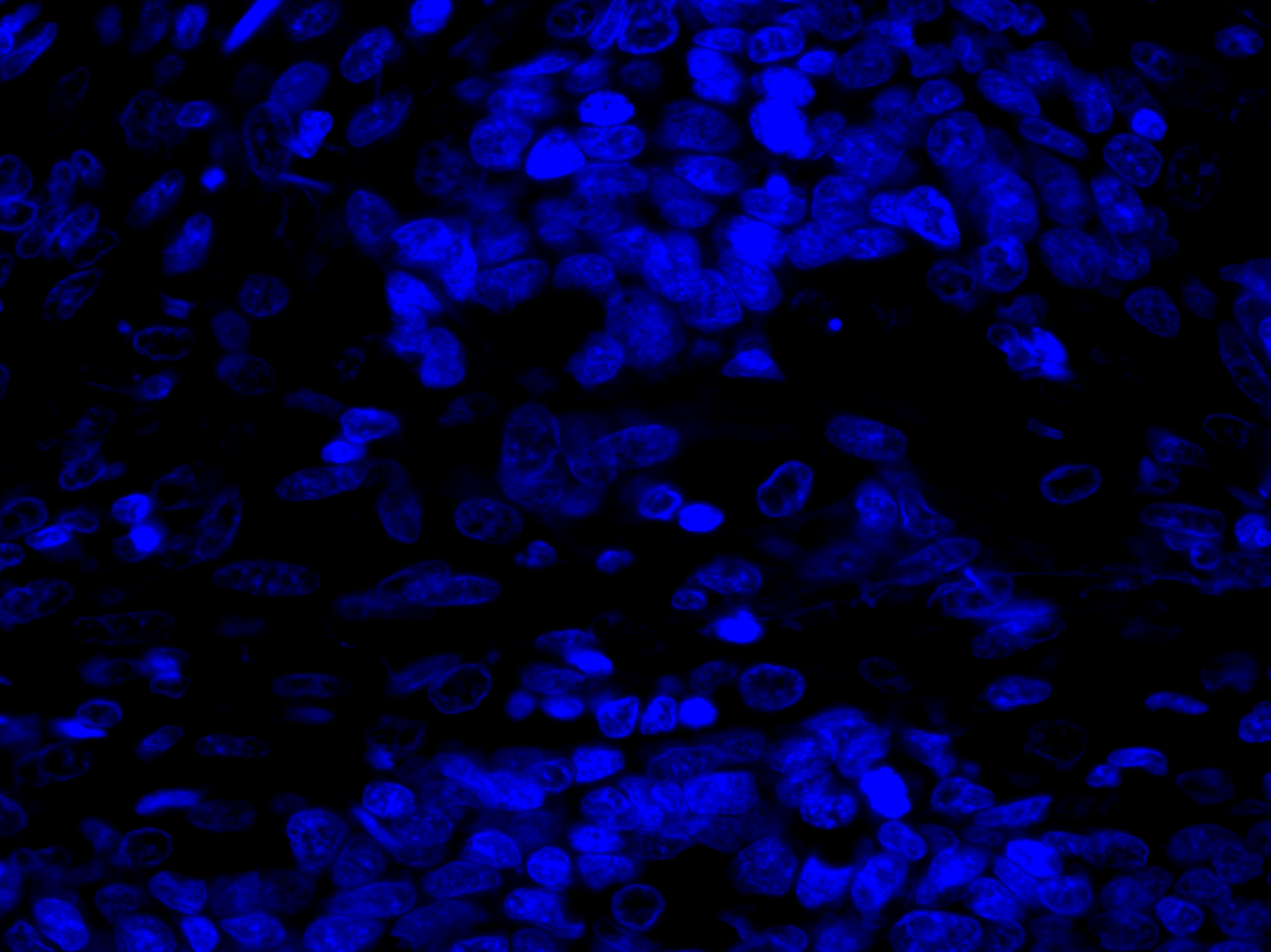

Supplement: Supplementary file 2 — Source data Fig. 1 [file 44321_2024_64_MOESM2_ESM.zip › Figure 1/Figure 1A/SCC/MC08-2wH sq 12b 40X-1_c2.TIF]

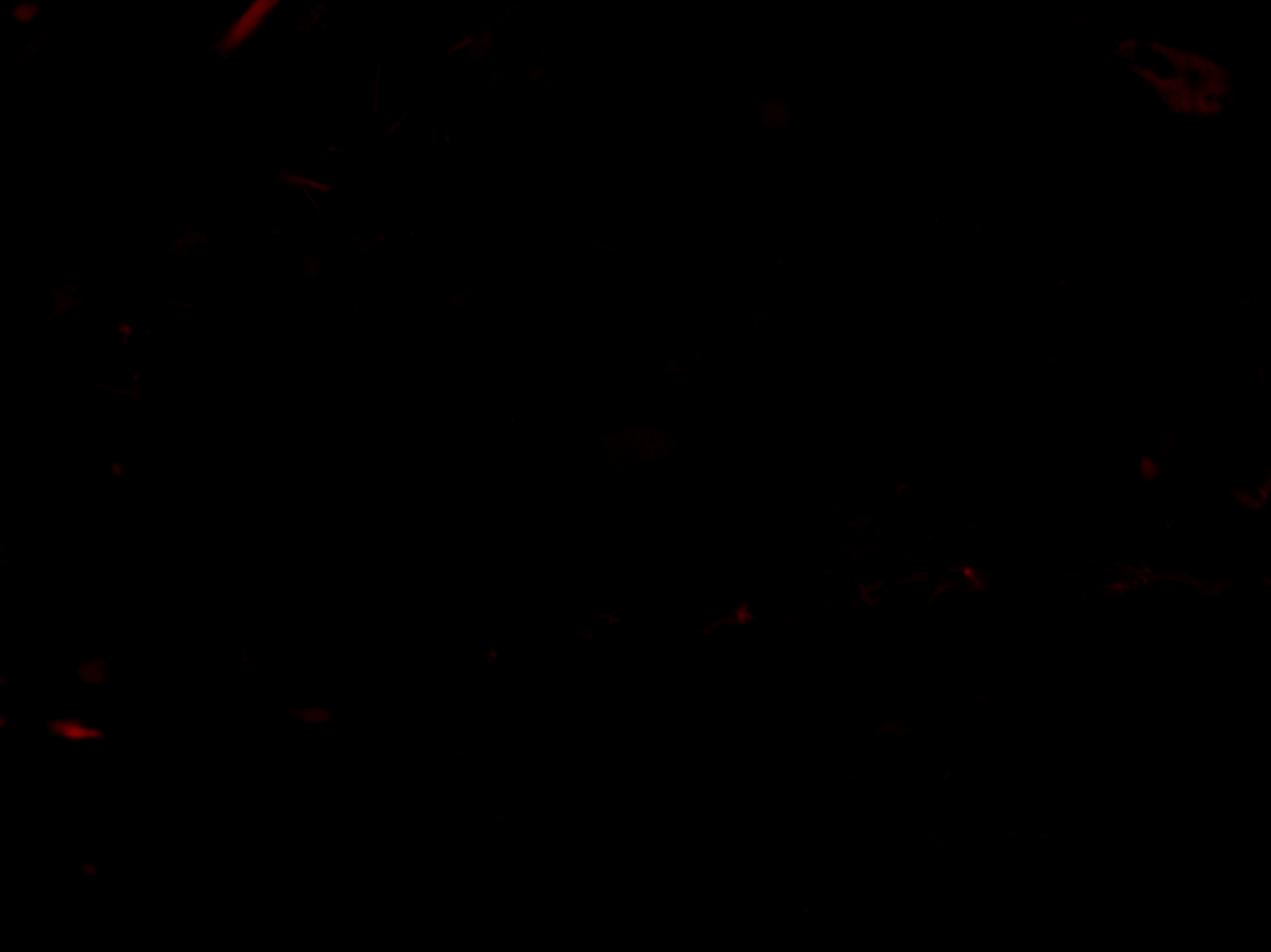

Supplement: Supplementary file 2 — Source data Fig. 1 [file 44321_2024_64_MOESM2_ESM.zip › Figure 1/Figure 1A/SCC/MC08-2wH sq 12b 40X-1_c3.TIF]

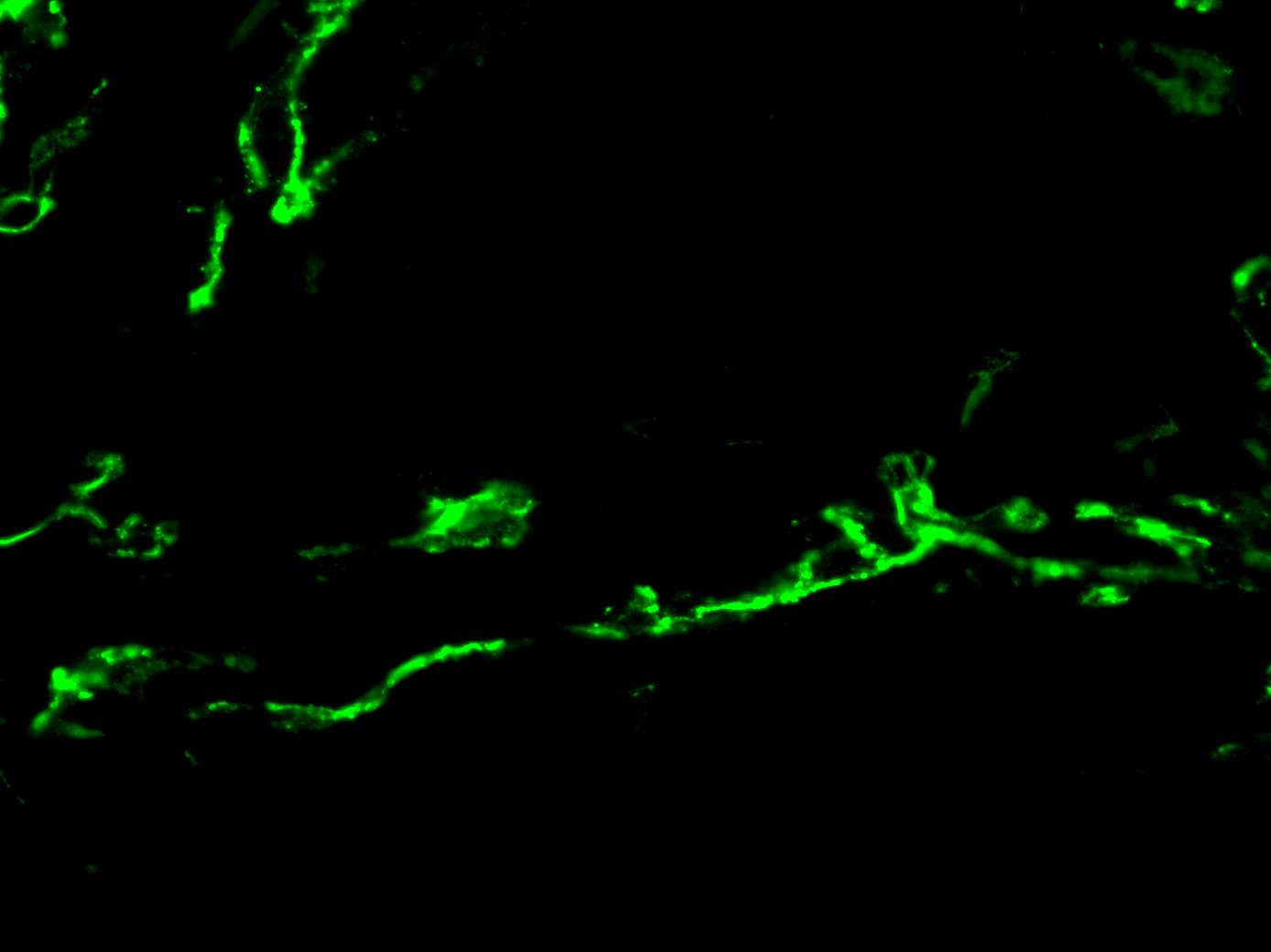

Supplement: Supplementary file 2 — Source data Fig. 1 [file 44321_2024_64_MOESM2_ESM.zip › Figure 1/Figure 1A/SCC/MC08-2wH sq 12b 40X-1_c4.TIF]

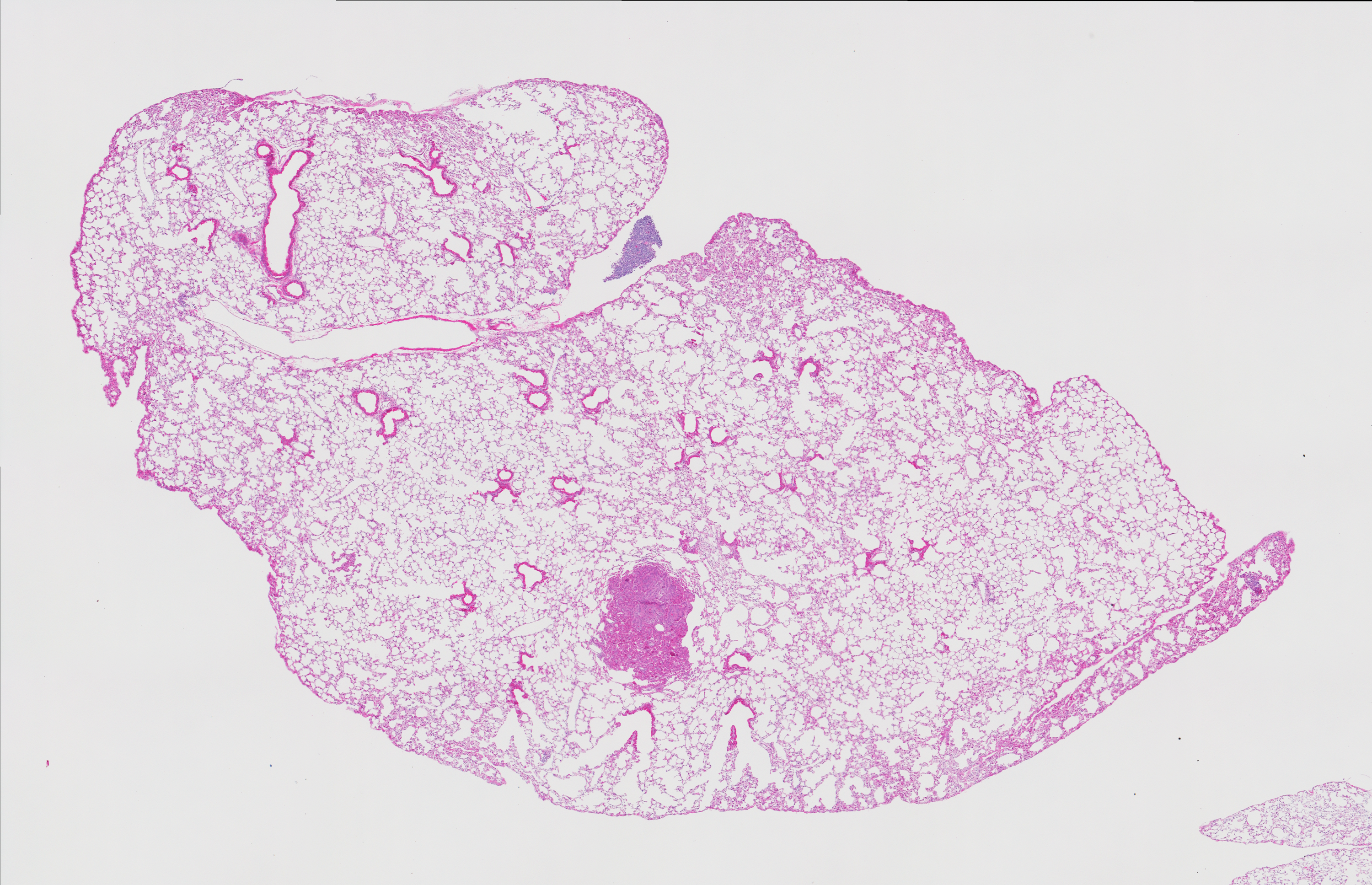

Supplement: Supplementary file 3 — Source data Fig. 2 [file 44321_2024_64_MOESM3_ESM.zip › Figure 2/Figure 2H/Control.tif]

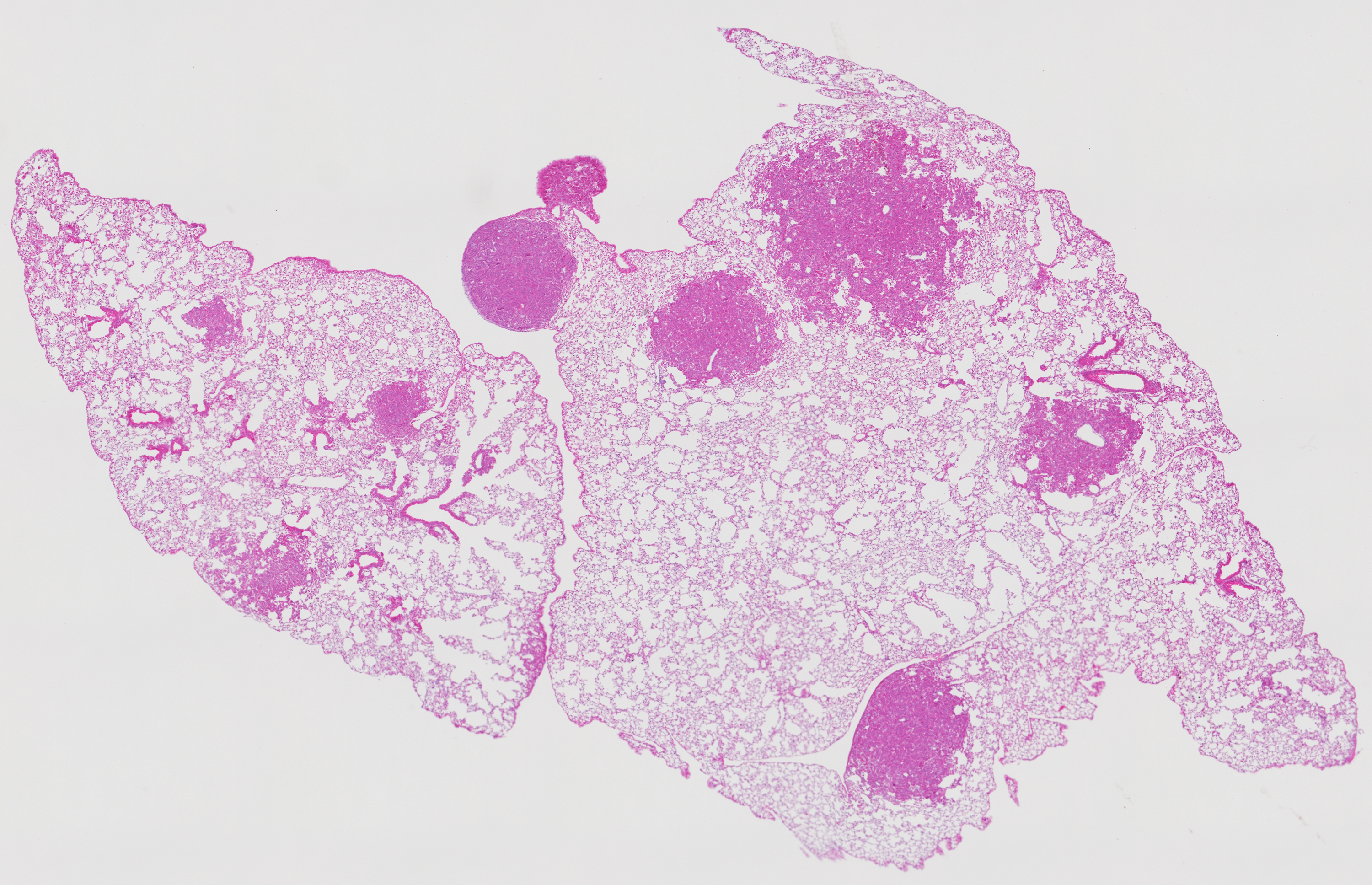

Supplement: Supplementary file 3 — Source data Fig. 2 [file 44321_2024_64_MOESM3_ESM.zip › Figure 2/Figure 2H/endFoxf1+-.tif]

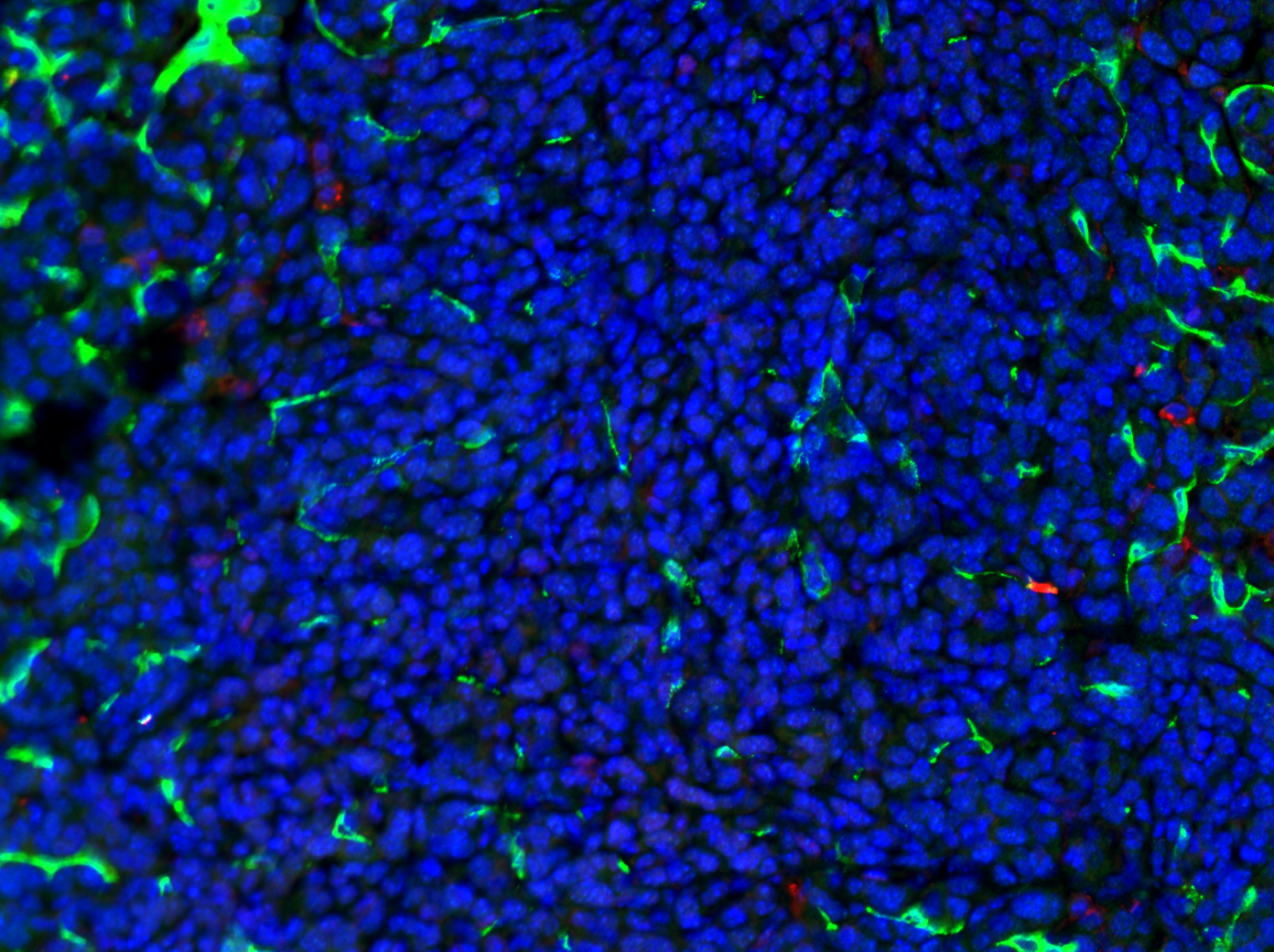

Supplement: Supplementary file 4 — Source data Fig. 3 [file 44321_2024_64_MOESM4_ESM.zip › Figure 3/Figure 3G Left panel/Control/Con#3 20X-1_RGB.tif]

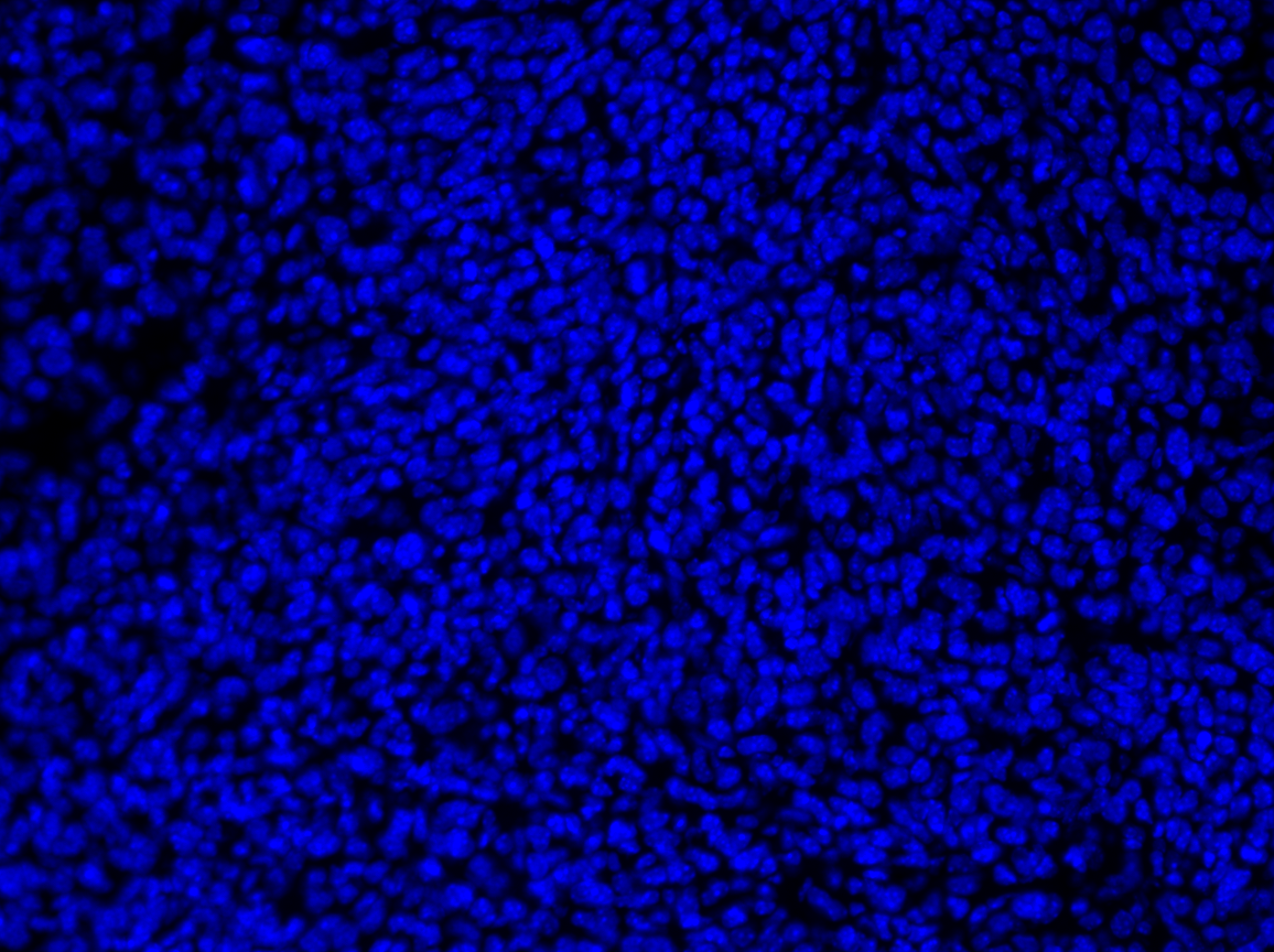

Supplement: Supplementary file 4 — Source data Fig. 3 [file 44321_2024_64_MOESM4_ESM.zip › Figure 3/Figure 3G Left panel/Control/Con#3 20X-1_RGB_DAPI MONO.tif]

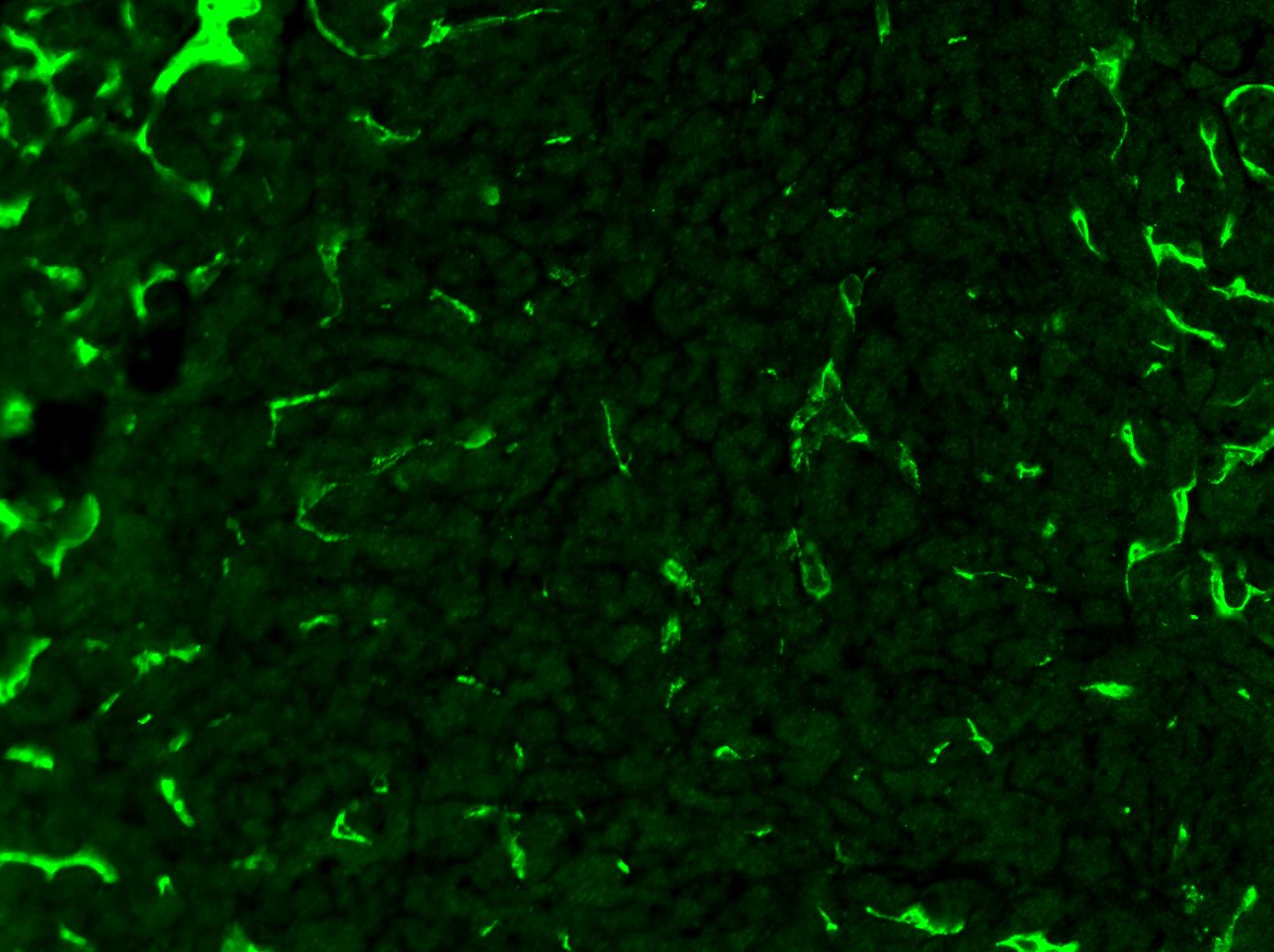

Supplement: Supplementary file 4 — Source data Fig. 3 [file 44321_2024_64_MOESM4_ESM.zip › Figure 3/Figure 3G Left panel/Control/Con#3 20X-1_RGB_FITC MONO.tif]

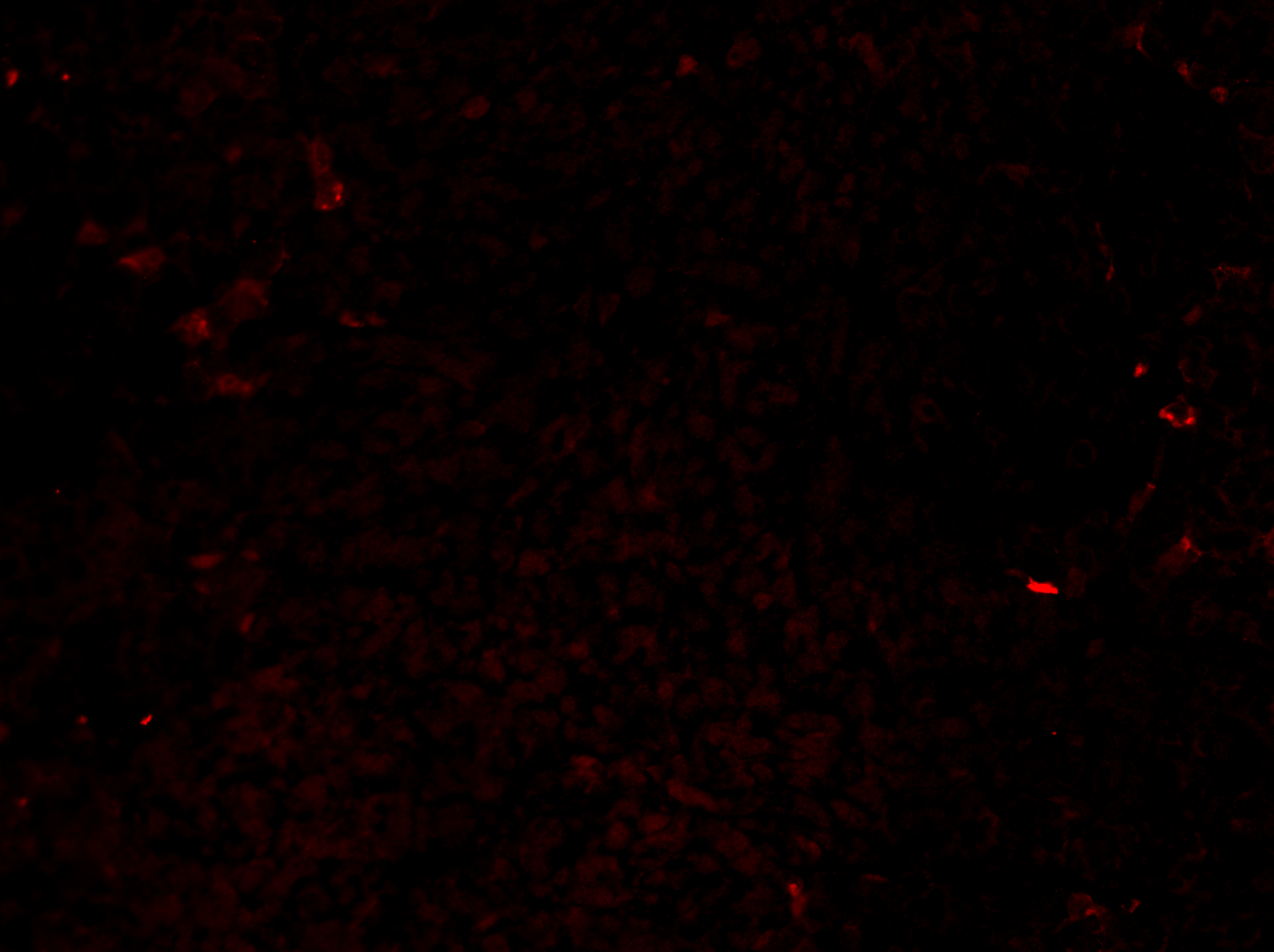

Supplement: Supplementary file 4 — Source data Fig. 3 [file 44321_2024_64_MOESM4_ESM.zip › Figure 3/Figure 3G Left panel/Control/Con#3 20X-1_RGB_TRITC MONO.tif]

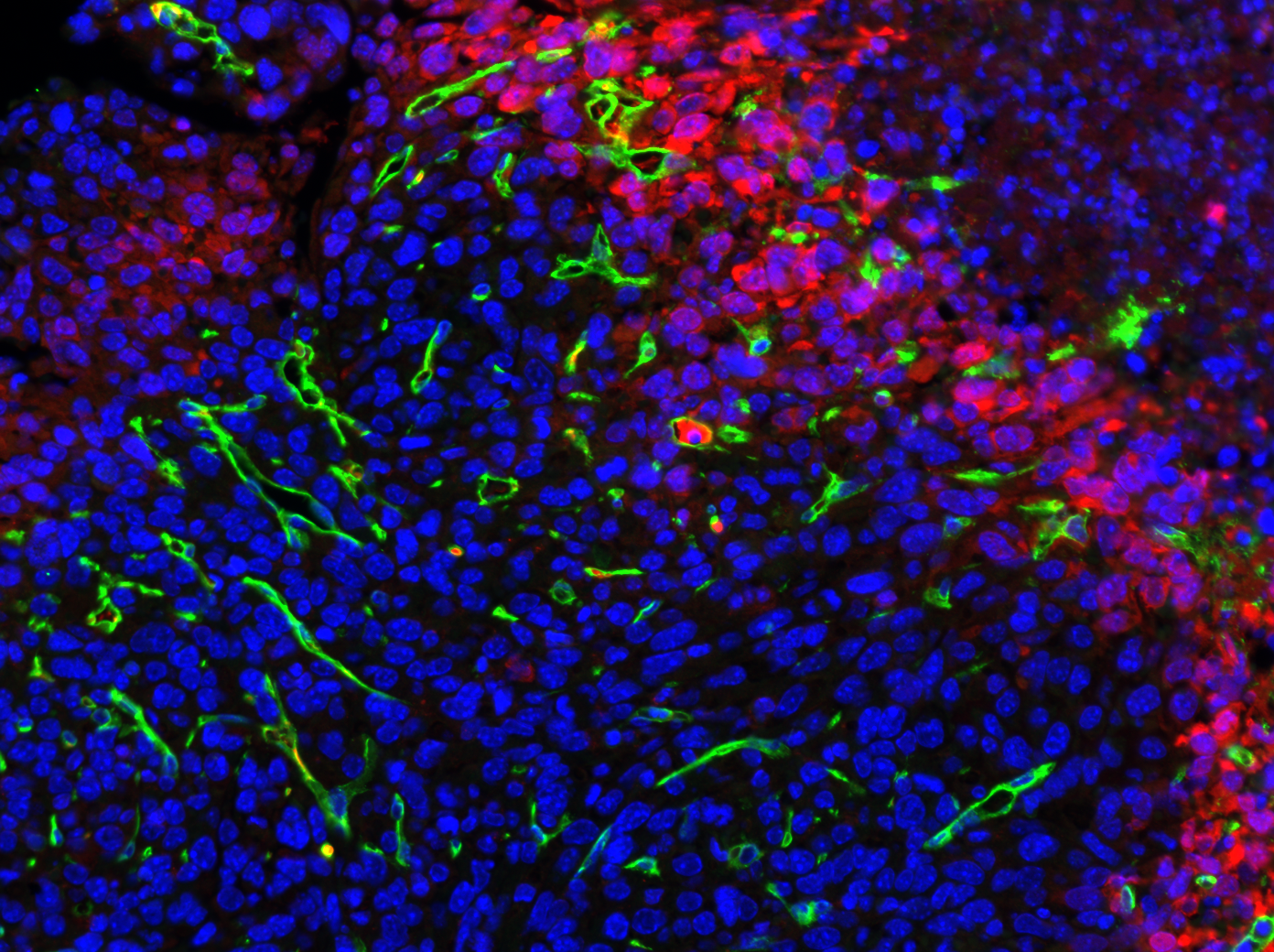

Supplement: Supplementary file 4 — Source data Fig. 3 [file 44321_2024_64_MOESM4_ESM.zip › Figure 3/Figure 3G Left panel/Het/Het#7 20X-2_RGB.tif]

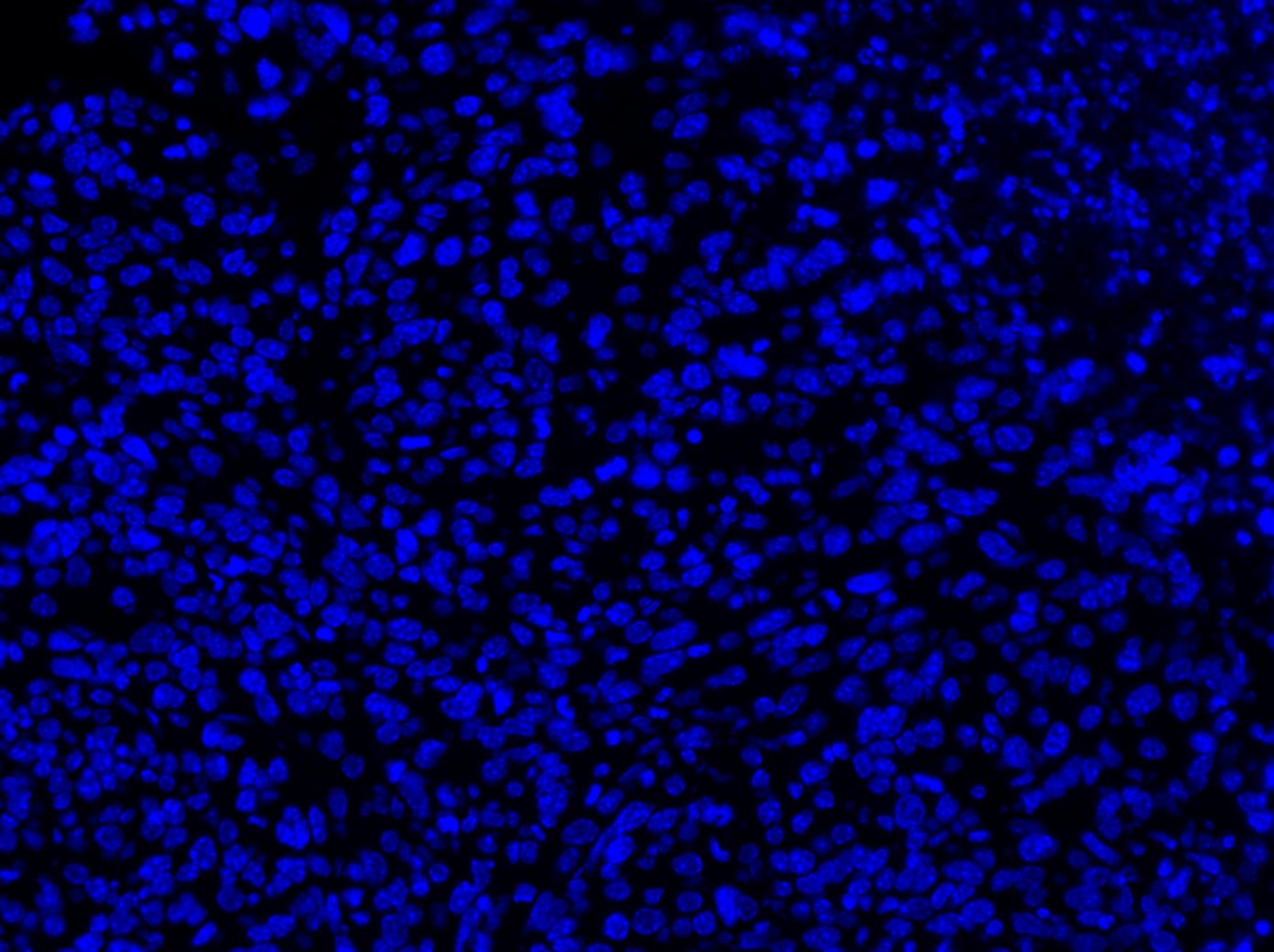

Supplement: Supplementary file 4 — Source data Fig. 3 [file 44321_2024_64_MOESM4_ESM.zip › Figure 3/Figure 3G Left panel/Het/Het#7 20X-2_RGB_DAPI MONO.tif]

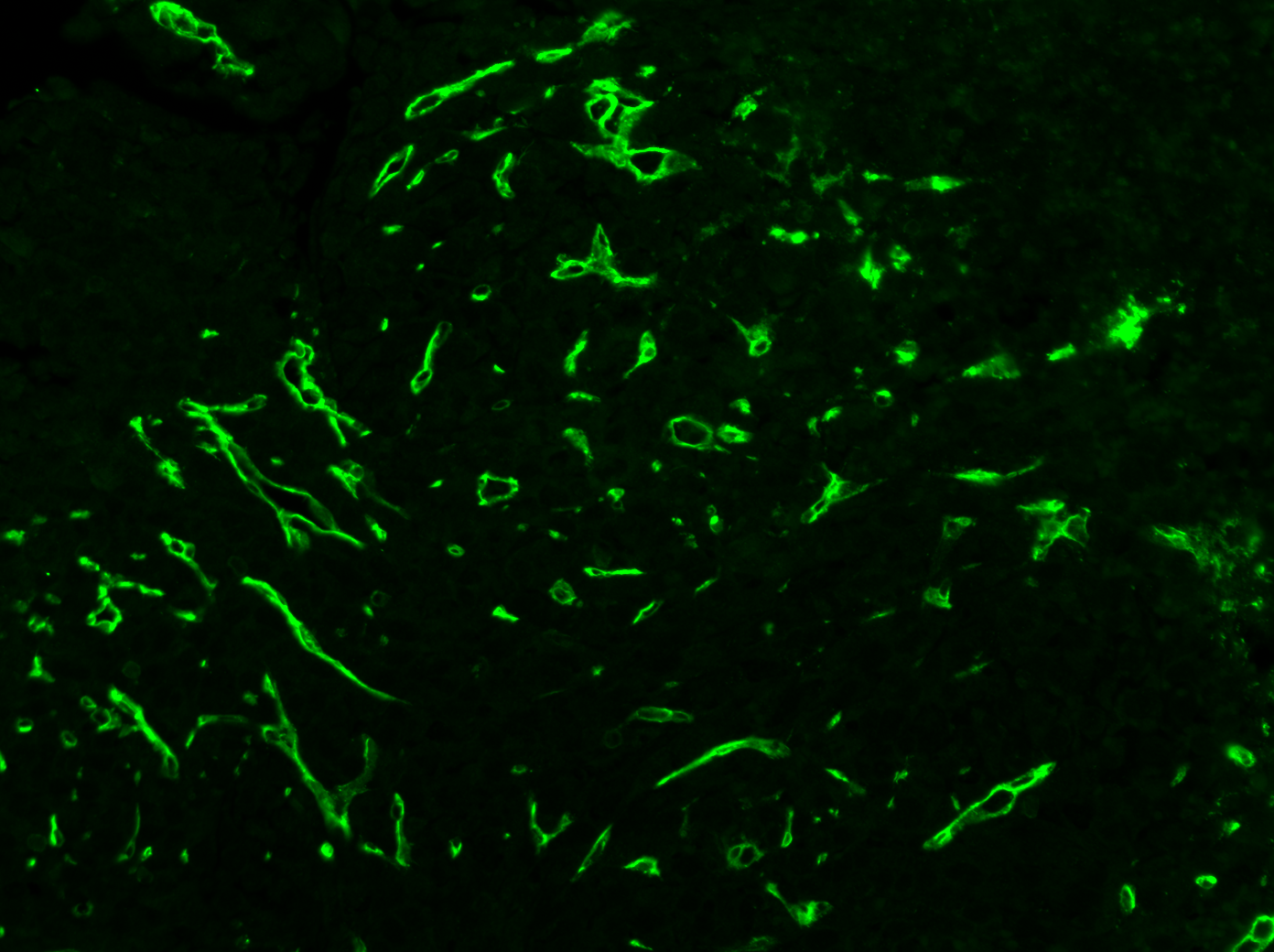

Supplement: Supplementary file 4 — Source data Fig. 3 [file 44321_2024_64_MOESM4_ESM.zip › Figure 3/Figure 3G Left panel/Het/Het#7 20X-2_RGB_FITC MONO.tif]

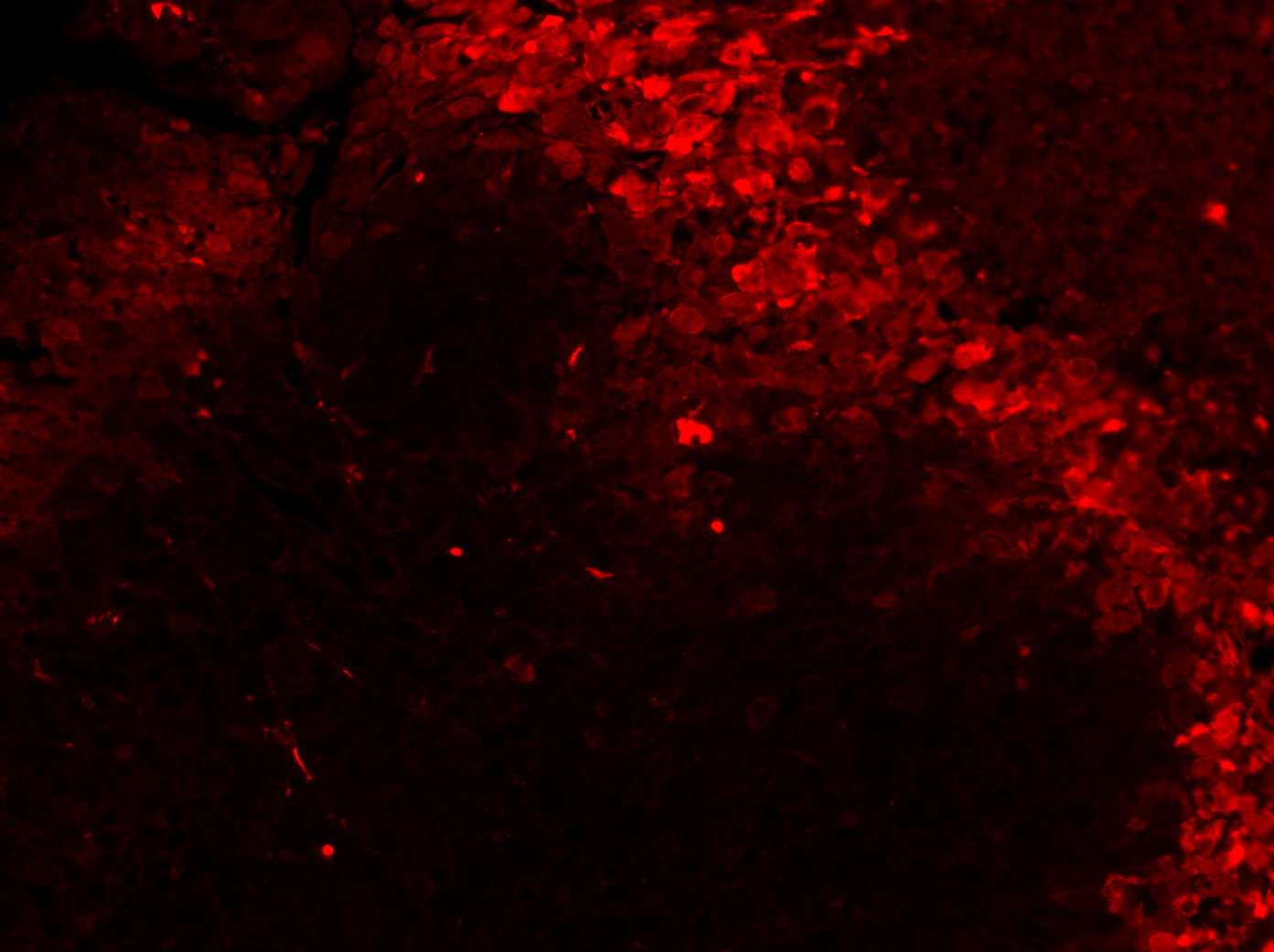

Supplement: Supplementary file 4 — Source data Fig. 3 [file 44321_2024_64_MOESM4_ESM.zip › Figure 3/Figure 3G Left panel/Het/Het#7 20X-2_RGB_TRITC MONO.tif]

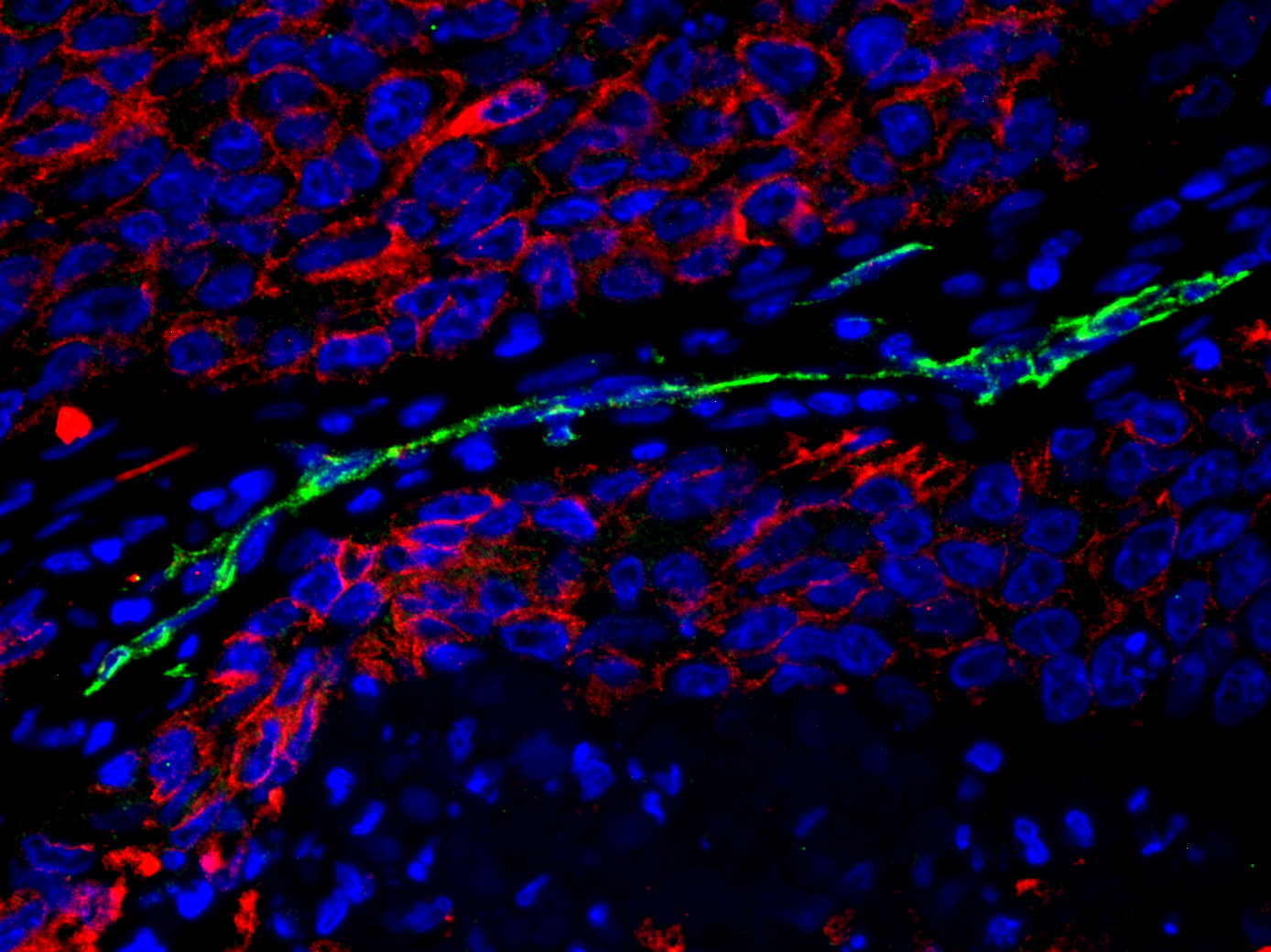

Supplement: Supplementary file 5 — Source data Fig. 4 [file 44321_2024_64_MOESM5_ESM.zip › Figure 4/Figure 4G/Adenocarcinoma/MC-08-1wH ad 6B 40X-5_(c1+c3+c4).TIF]

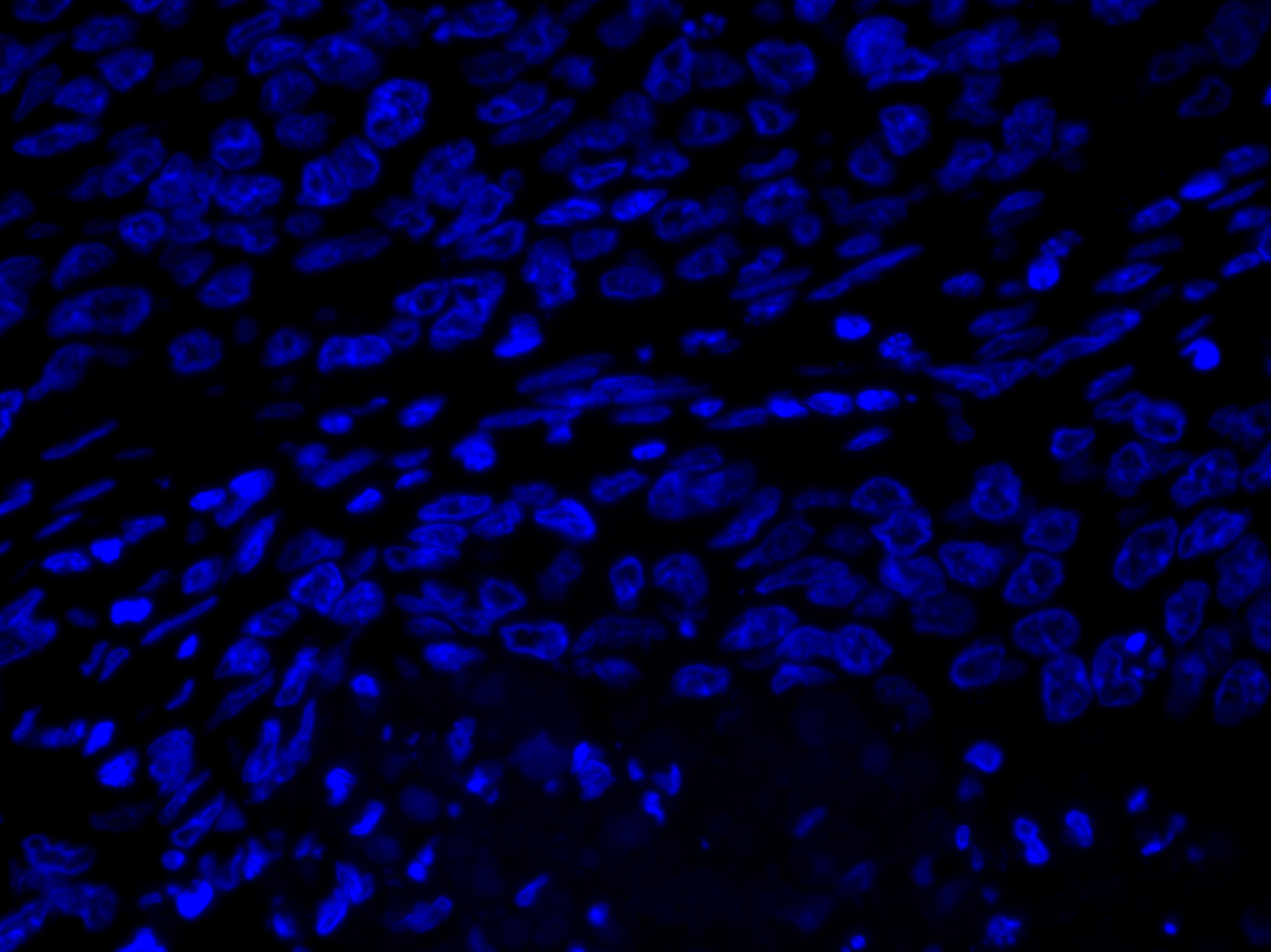

Supplement: Supplementary file 5 — Source data Fig. 4 [file 44321_2024_64_MOESM5_ESM.zip › Figure 4/Figure 4G/Adenocarcinoma/MC-08-1wH ad 6B 40X-5_c1.TIF]

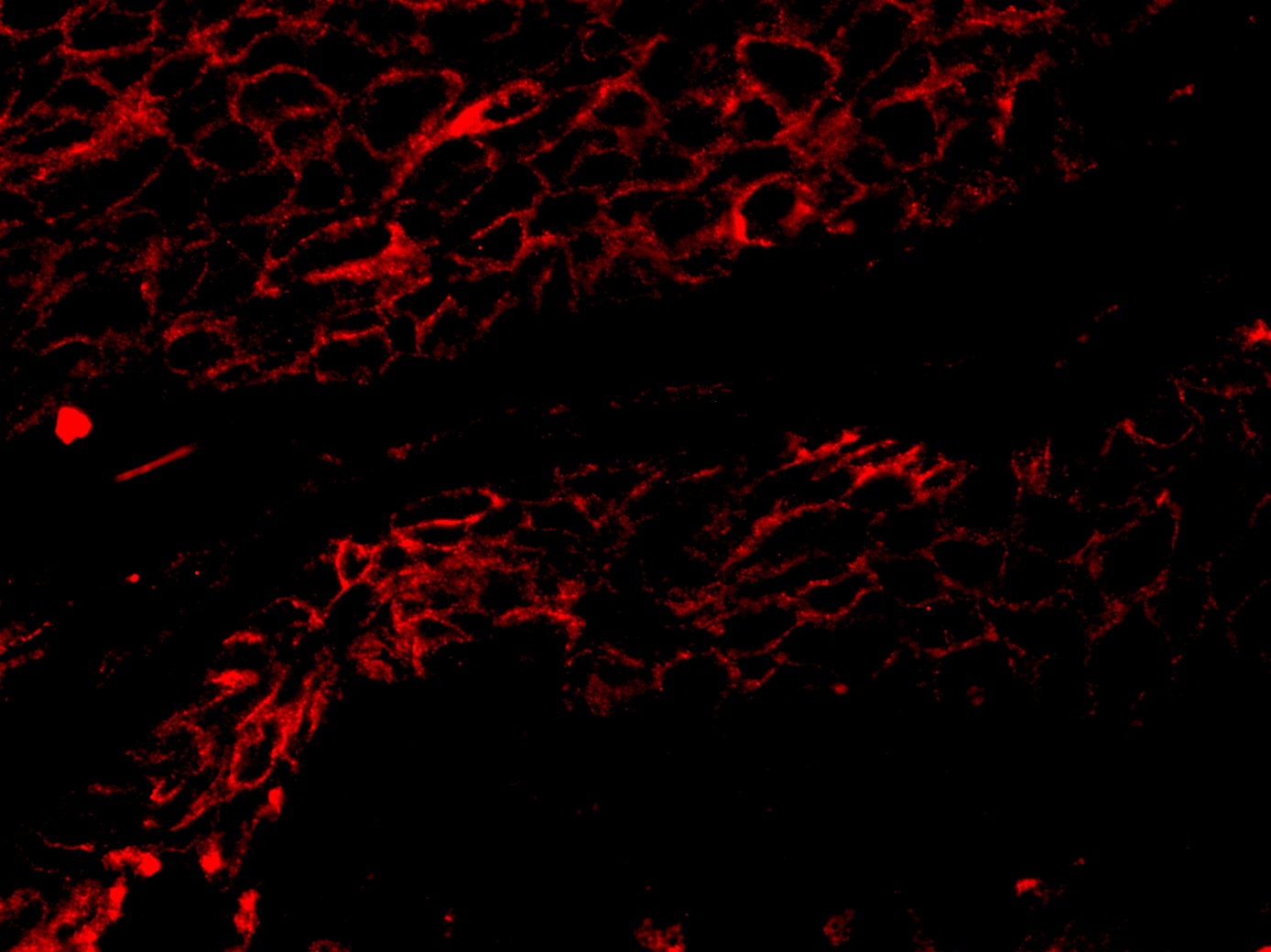

Supplement: Supplementary file 5 — Source data Fig. 4 [file 44321_2024_64_MOESM5_ESM.zip › Figure 4/Figure 4G/Adenocarcinoma/MC-08-1wH ad 6B 40X-5_c3.TIF]

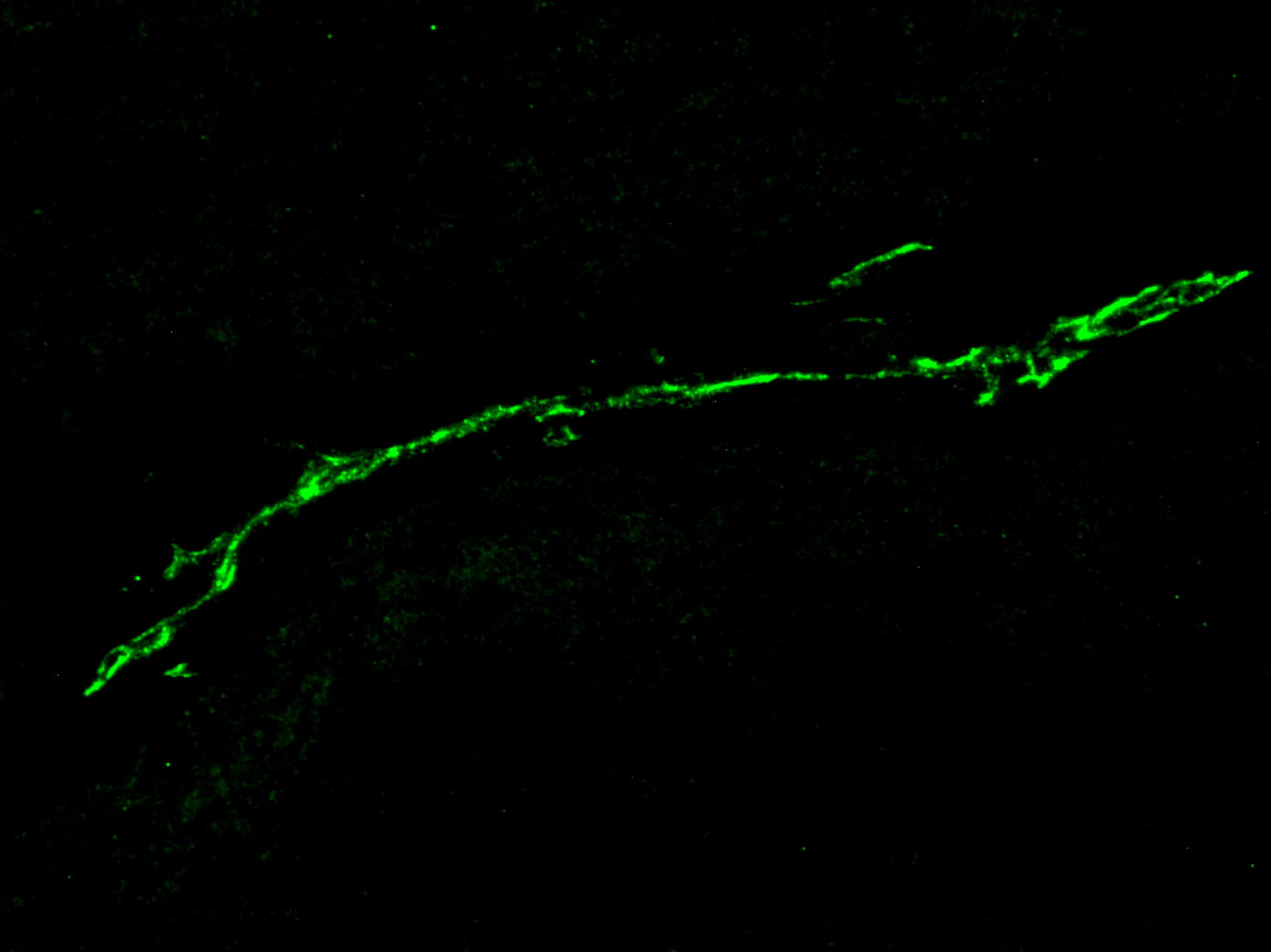

Supplement: Supplementary file 5 — Source data Fig. 4 [file 44321_2024_64_MOESM5_ESM.zip › Figure 4/Figure 4G/Adenocarcinoma/MC-08-1wH ad 6B 40X-5_c4.TIF]

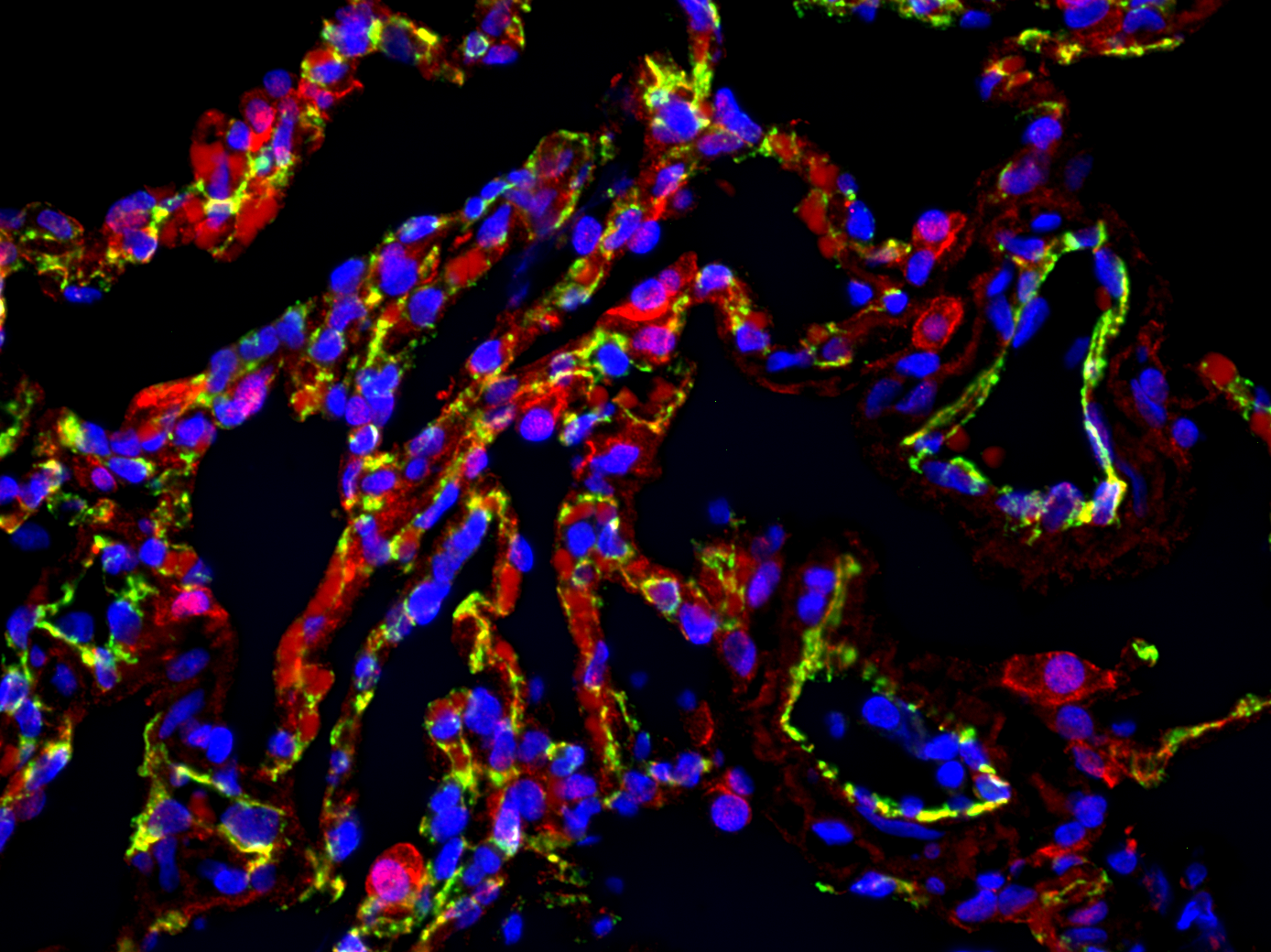

Supplement: Supplementary file 5 — Source data Fig. 4 [file 44321_2024_64_MOESM5_ESM.zip › Figure 4/Figure 4G/Donor Lung bottome panel/MC15-3H 40X-6_(c1+c3+c4).TIF]

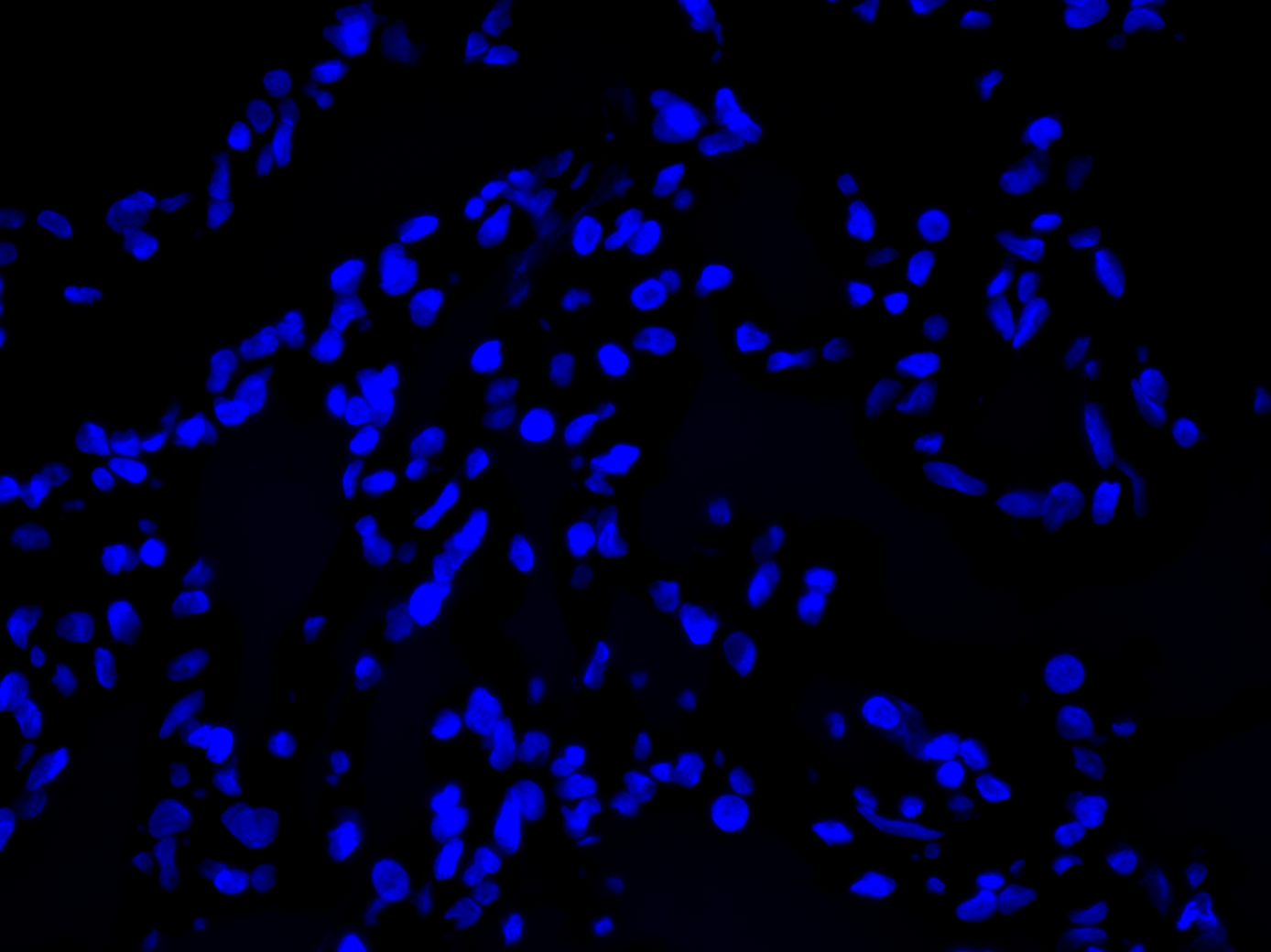

Supplement: Supplementary file 5 — Source data Fig. 4 [file 44321_2024_64_MOESM5_ESM.zip › Figure 4/Figure 4G/Donor Lung bottome panel/MC15-3H 40X-6_c1.TIF]

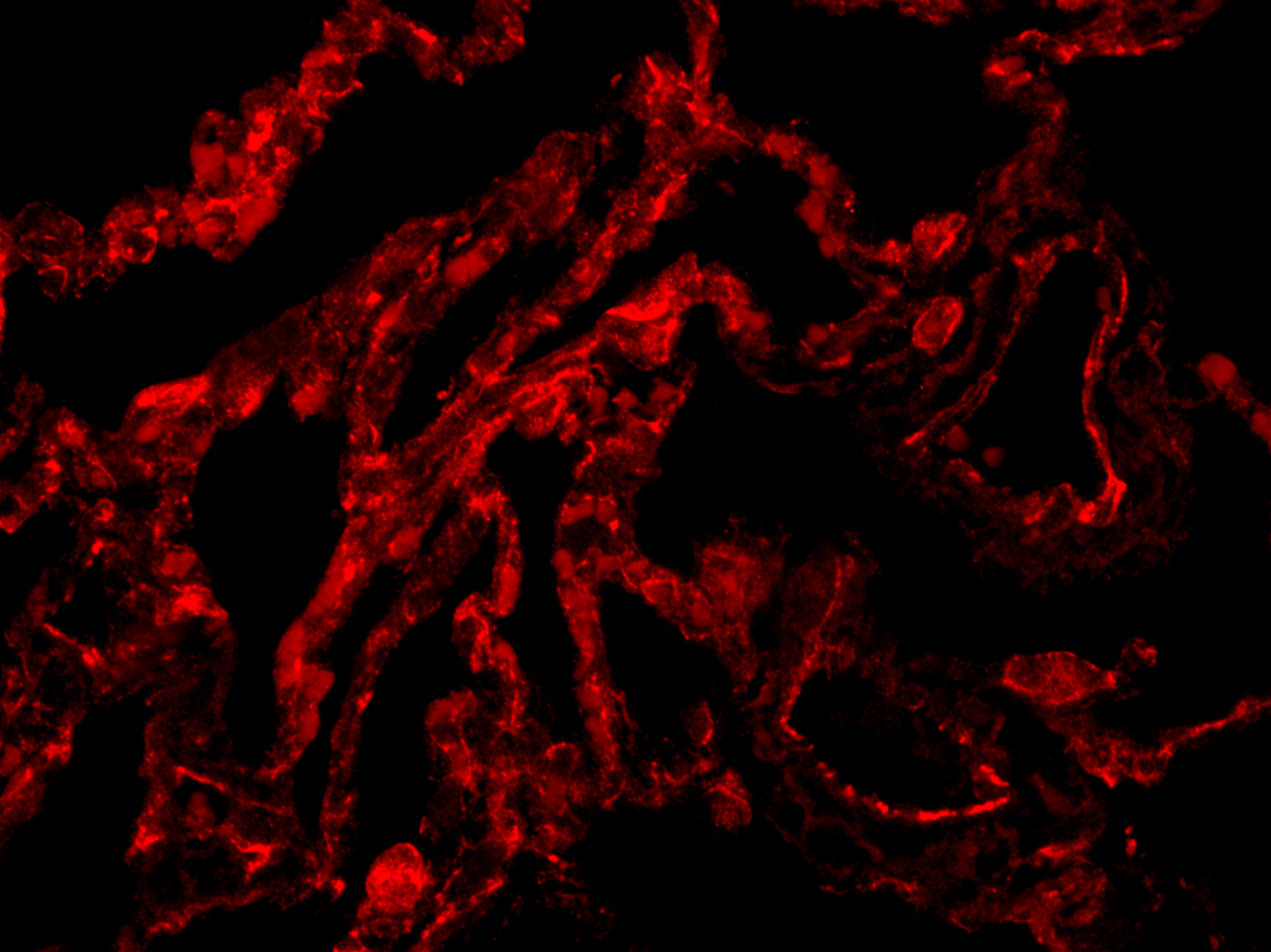

Supplement: Supplementary file 5 — Source data Fig. 4 [file 44321_2024_64_MOESM5_ESM.zip › Figure 4/Figure 4G/Donor Lung bottome panel/MC15-3H 40X-6_c3.TIF]

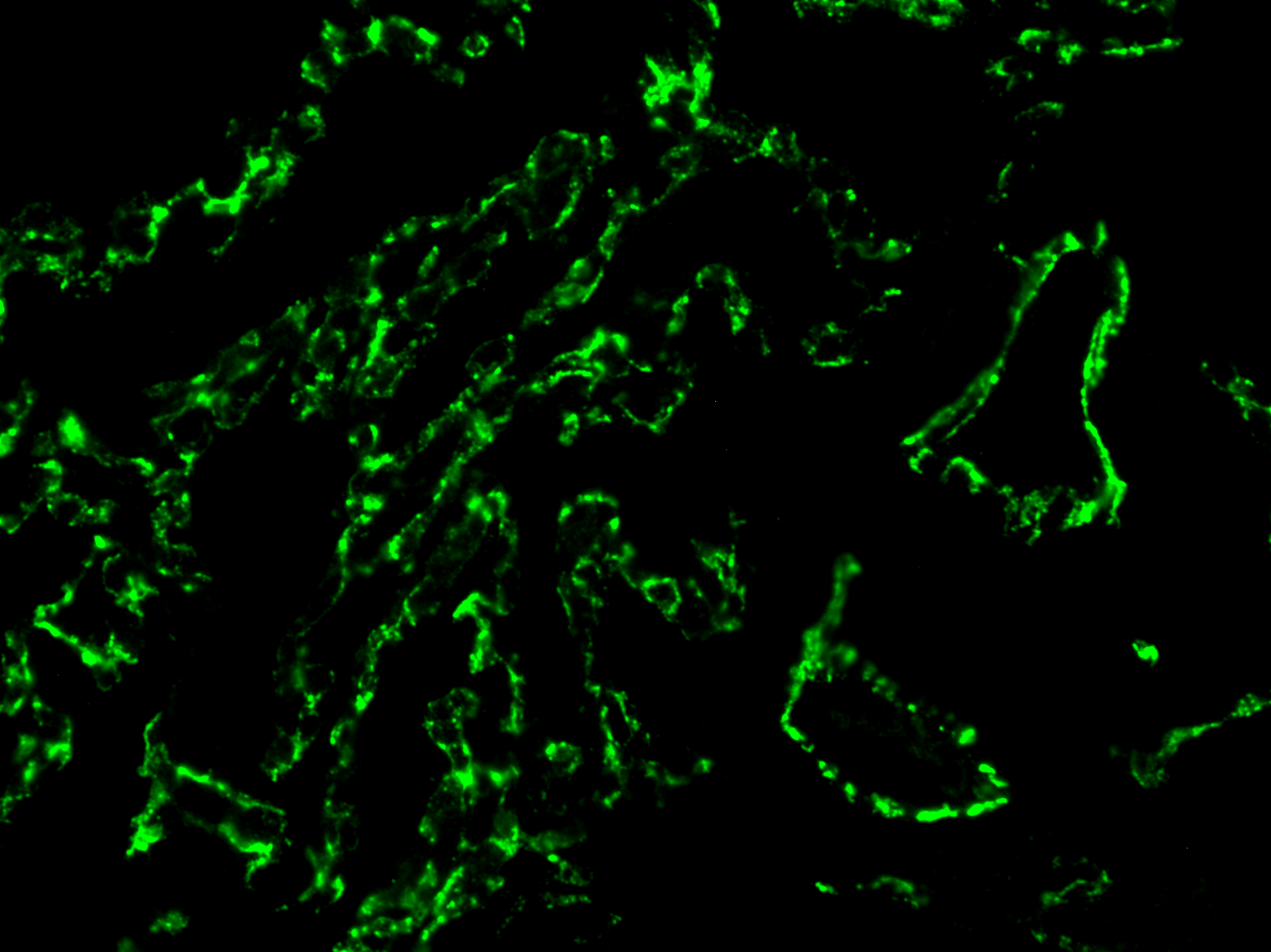

Supplement: Supplementary file 5 — Source data Fig. 4 [file 44321_2024_64_MOESM5_ESM.zip › Figure 4/Figure 4G/Donor Lung bottome panel/MC15-3H 40X-6_c4.TIF]

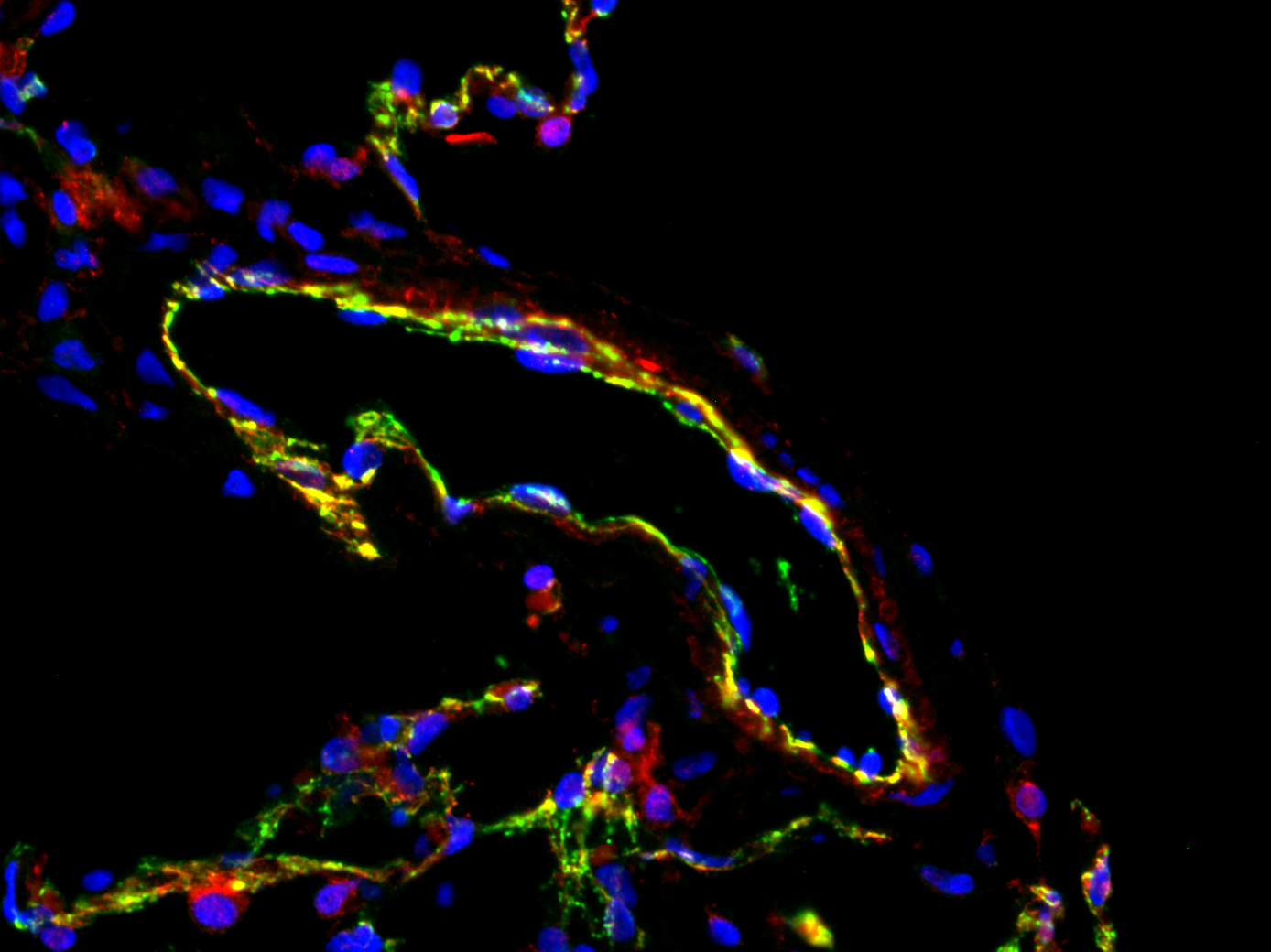

Supplement: Supplementary file 5 — Source data Fig. 4 [file 44321_2024_64_MOESM5_ESM.zip › Figure 4/Figure 4G/Donor Lung top panel/MC15-3H 40X-7_(c1+c3+c4).TIF]

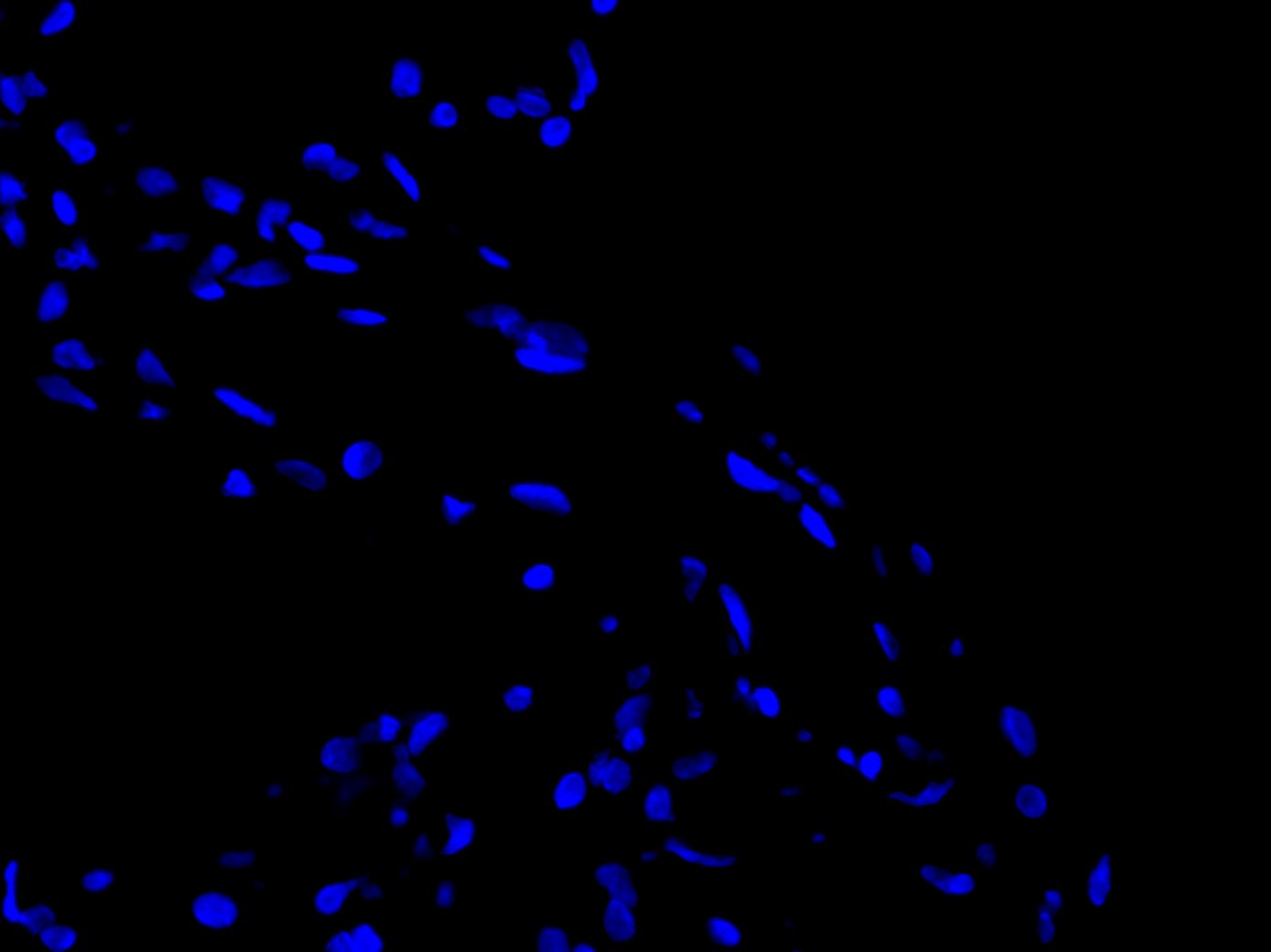

Supplement: Supplementary file 5 — Source data Fig. 4 [file 44321_2024_64_MOESM5_ESM.zip › Figure 4/Figure 4G/Donor Lung top panel/MC15-3H 40X-7_c1.TIF]

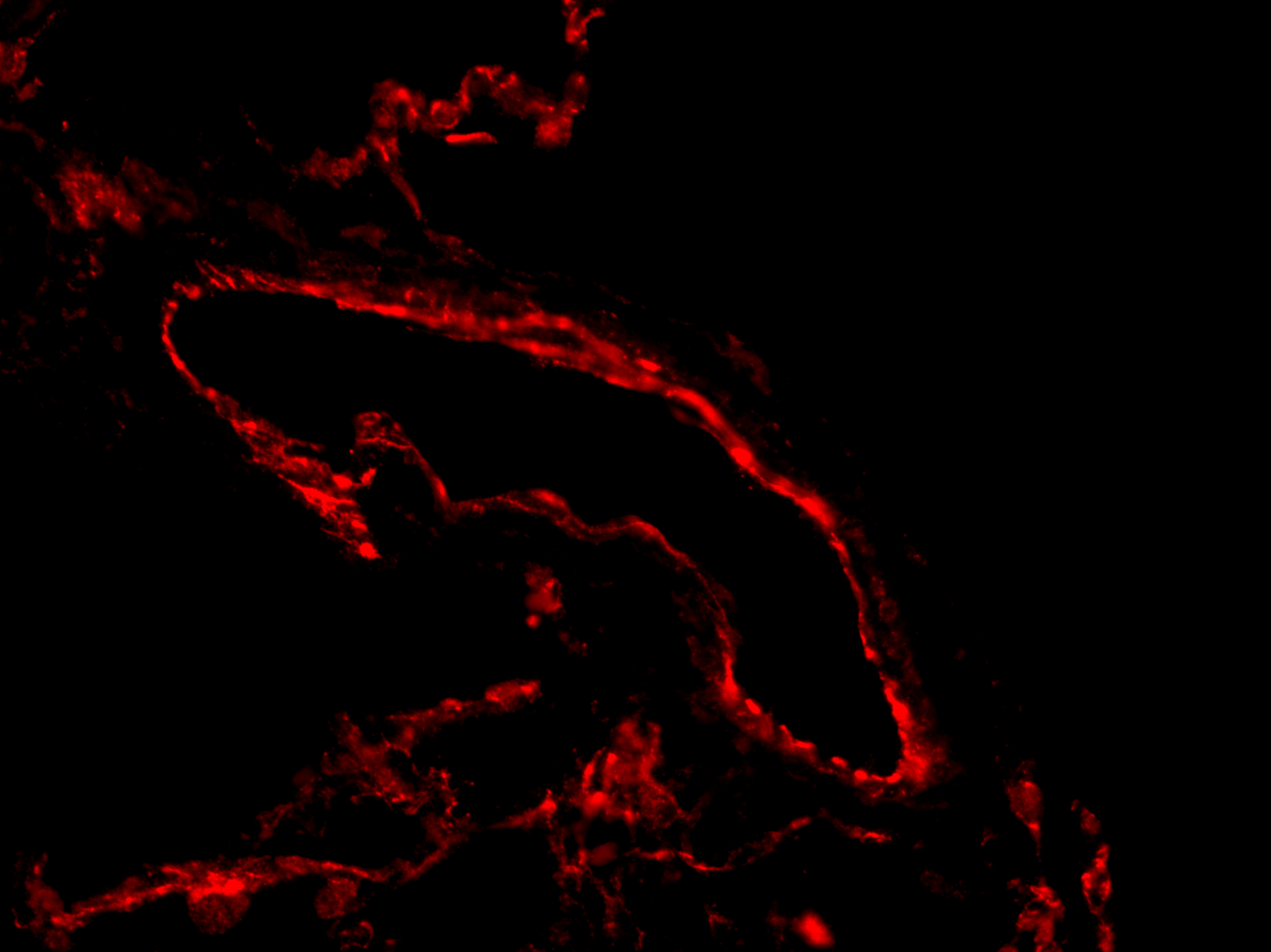

Supplement: Supplementary file 5 — Source data Fig. 4 [file 44321_2024_64_MOESM5_ESM.zip › Figure 4/Figure 4G/Donor Lung top panel/MC15-3H 40X-7_c3.TIF]

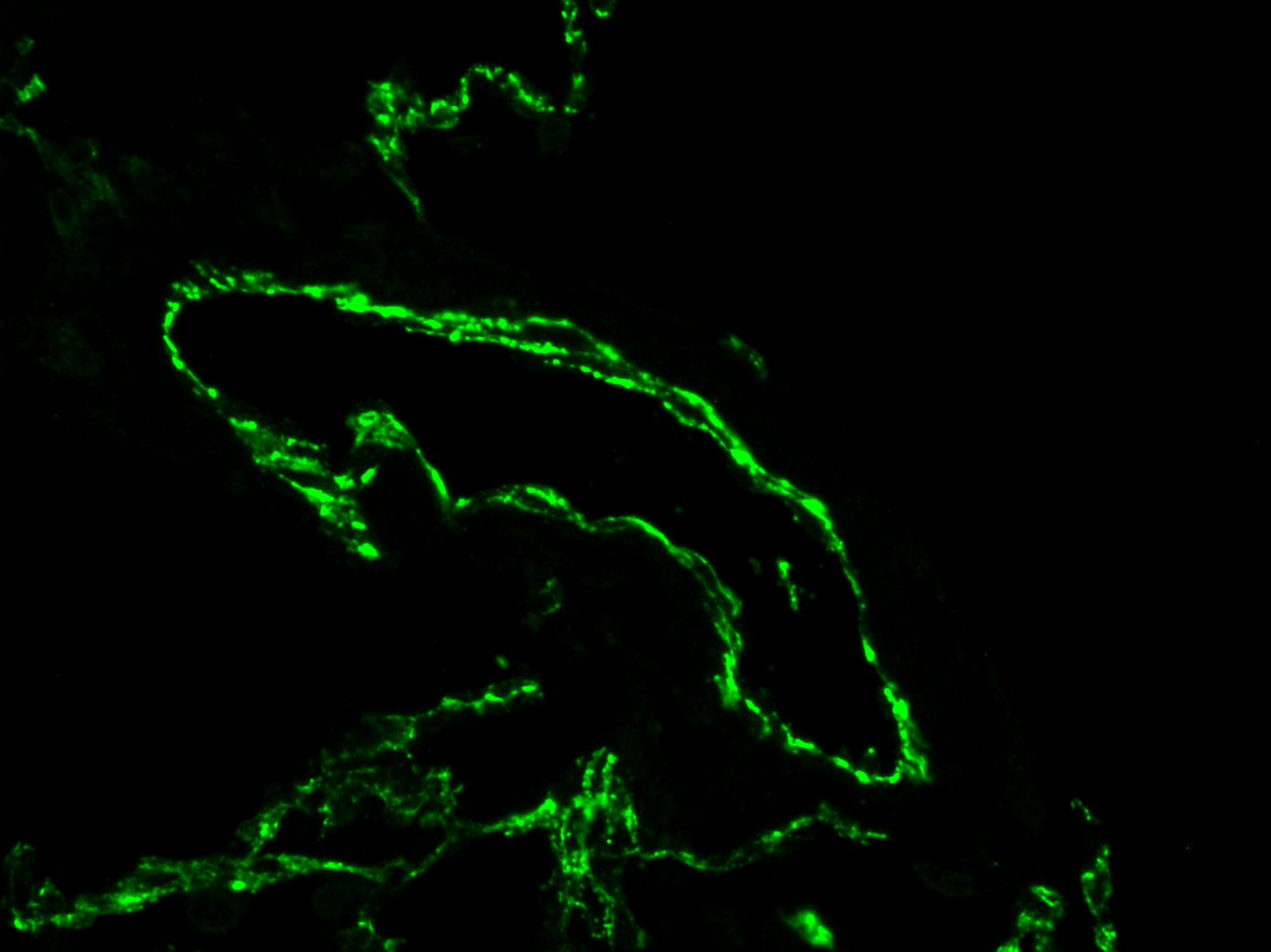

Supplement: Supplementary file 5 — Source data Fig. 4 [file 44321_2024_64_MOESM5_ESM.zip › Figure 4/Figure 4G/Donor Lung top panel/MC15-3H 40X-7_c4.TIF]

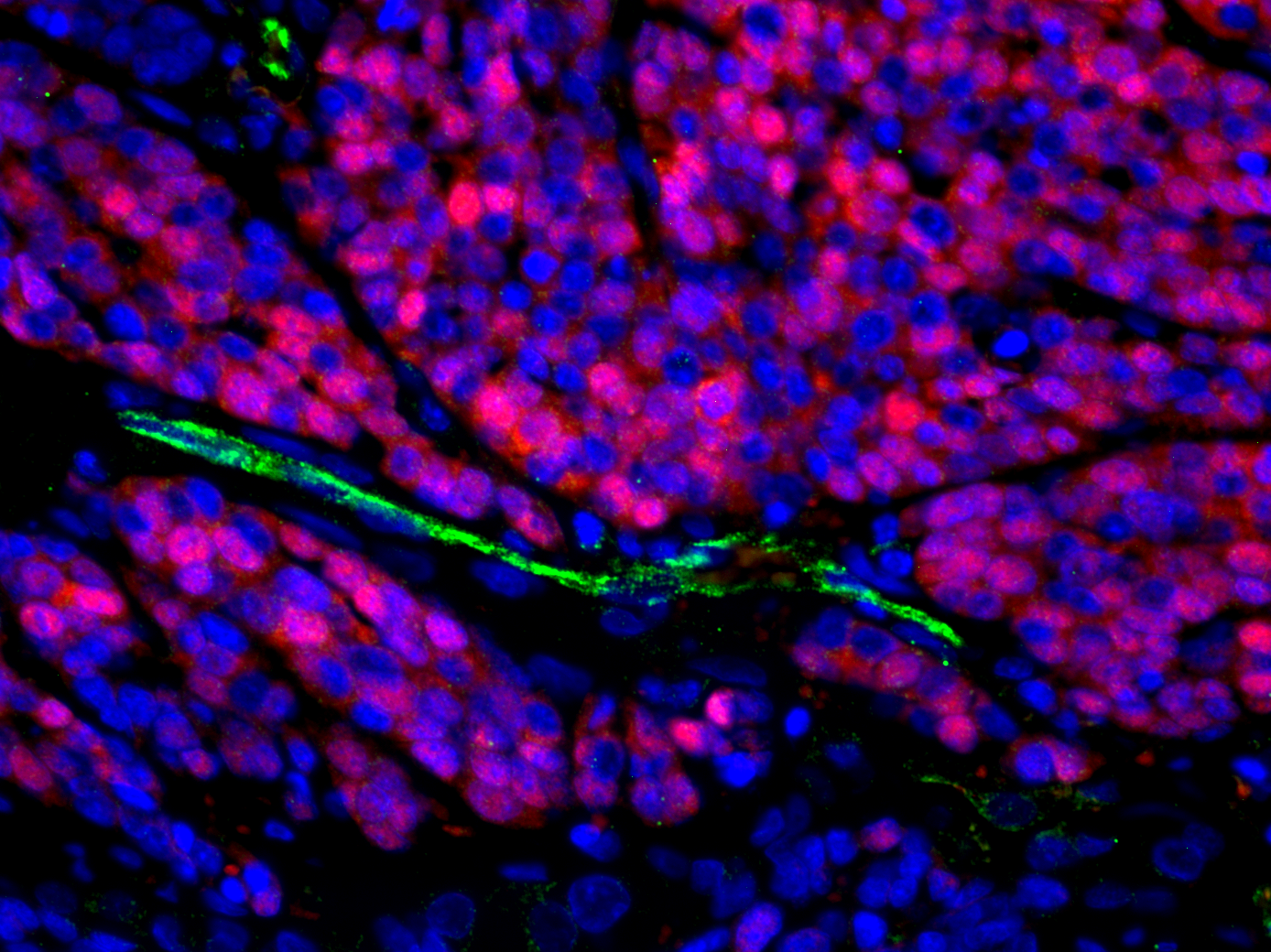

Supplement: Supplementary file 5 — Source data Fig. 4 [file 44321_2024_64_MOESM5_ESM.zip › Figure 4/Figure 4G/SCC/MC08-2wH sq 12B 40X-6_(c1+c3+c4).TIF]

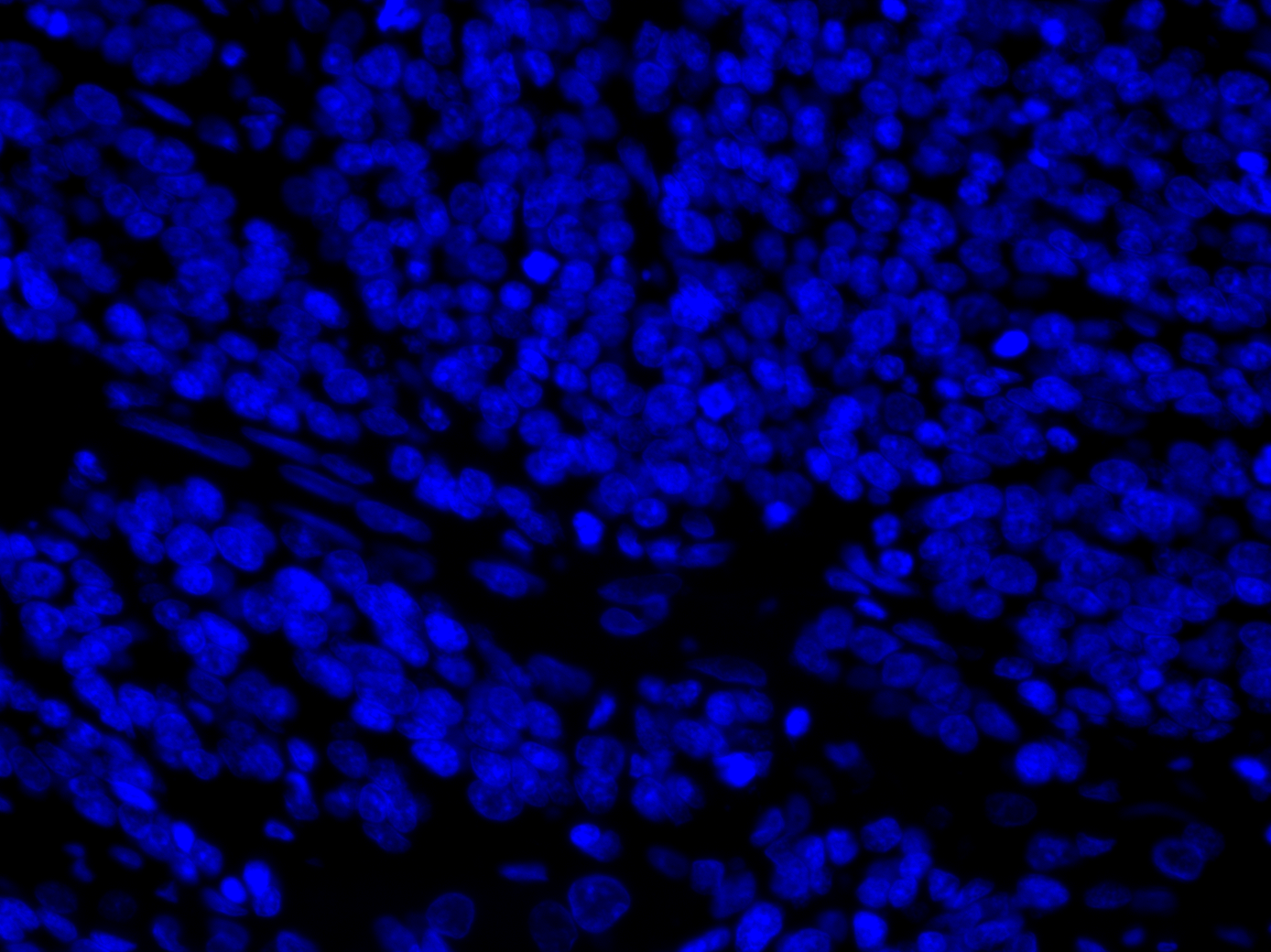

Supplement: Supplementary file 5 — Source data Fig. 4 [file 44321_2024_64_MOESM5_ESM.zip › Figure 4/Figure 4G/SCC/MC08-2wH sq 12B 40X-6_c1.TIF]

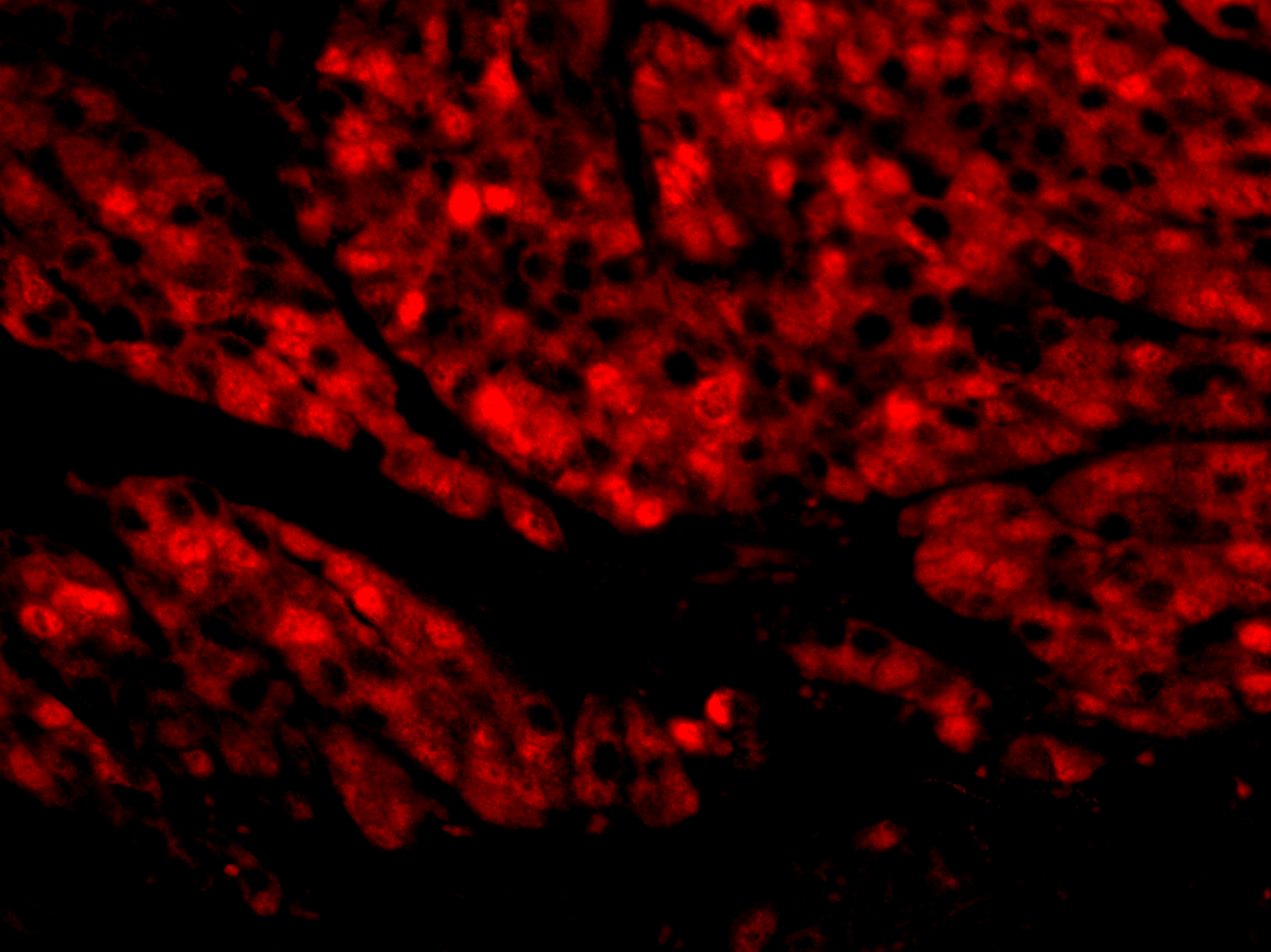

Supplement: Supplementary file 5 — Source data Fig. 4 [file 44321_2024_64_MOESM5_ESM.zip › Figure 4/Figure 4G/SCC/MC08-2wH sq 12B 40X-6_c3.TIF]

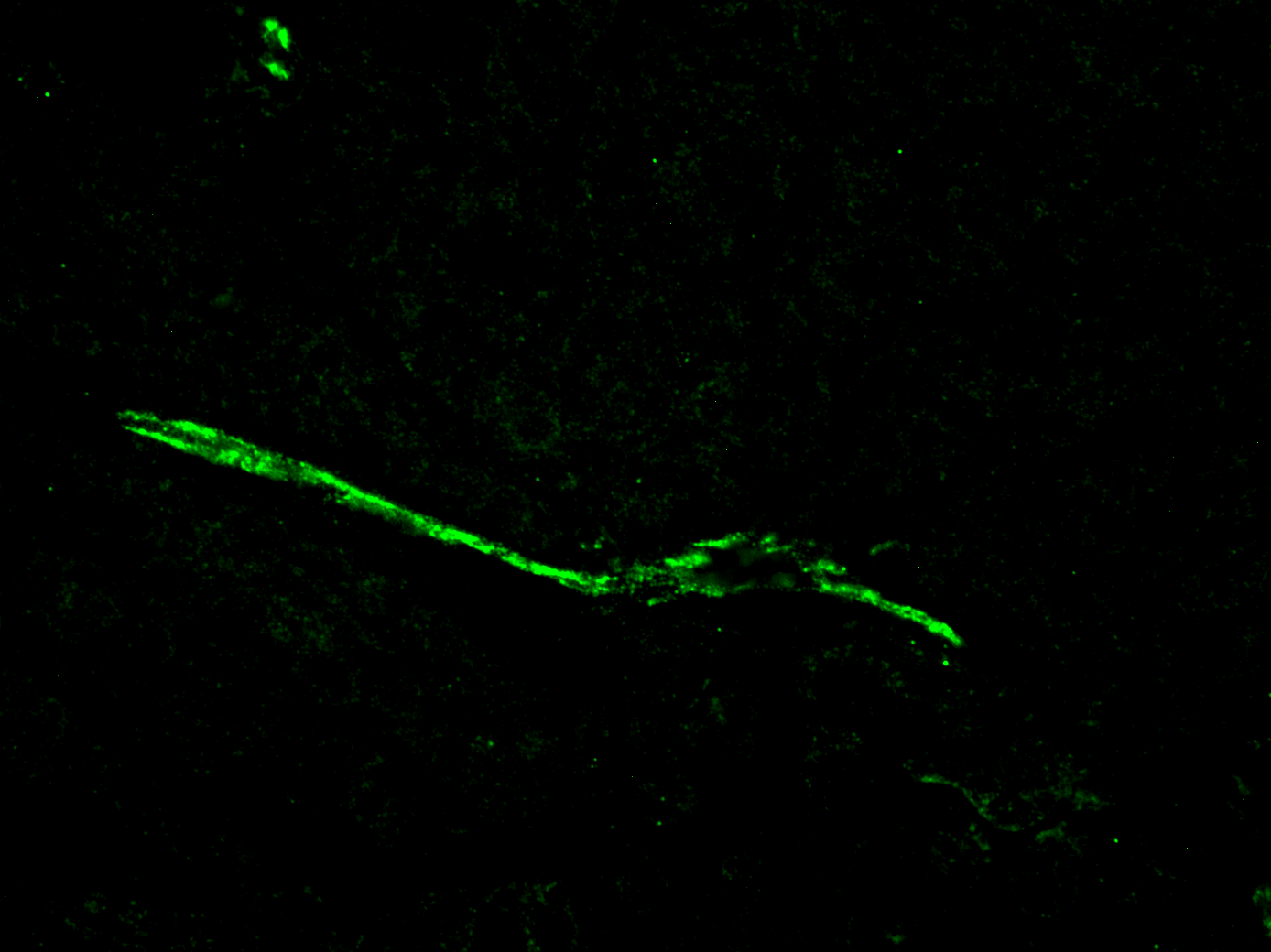

Supplement: Supplementary file 5 — Source data Fig. 4 [file 44321_2024_64_MOESM5_ESM.zip › Figure 4/Figure 4G/SCC/MC08-2wH sq 12B 40X-6_c4.TIF]

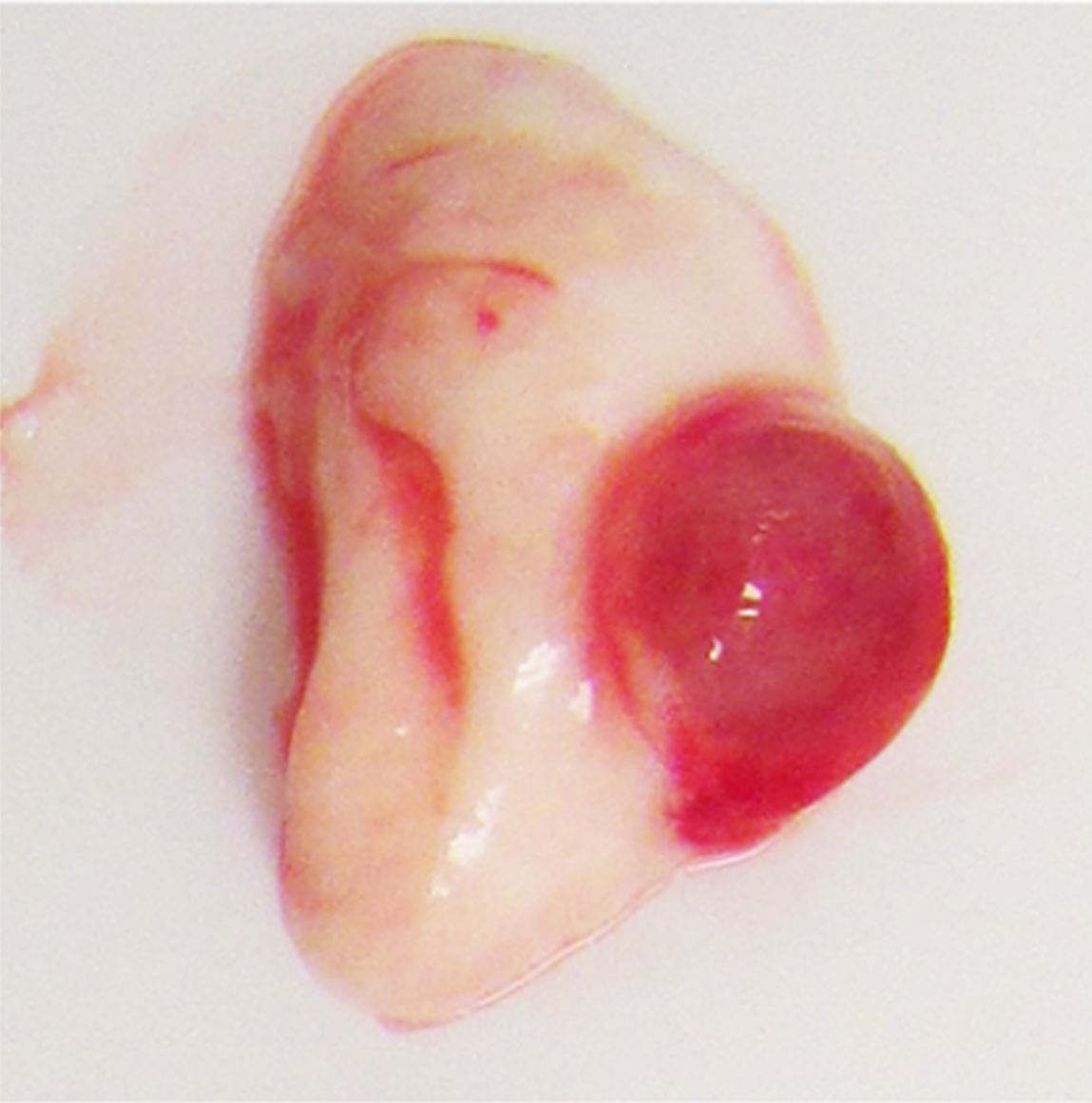

Supplement: Supplementary file 6 — Source data Fig. 5 [file 44321_2024_64_MOESM6_ESM.zip › Figure 5/Figure 5A/Control left.tif]

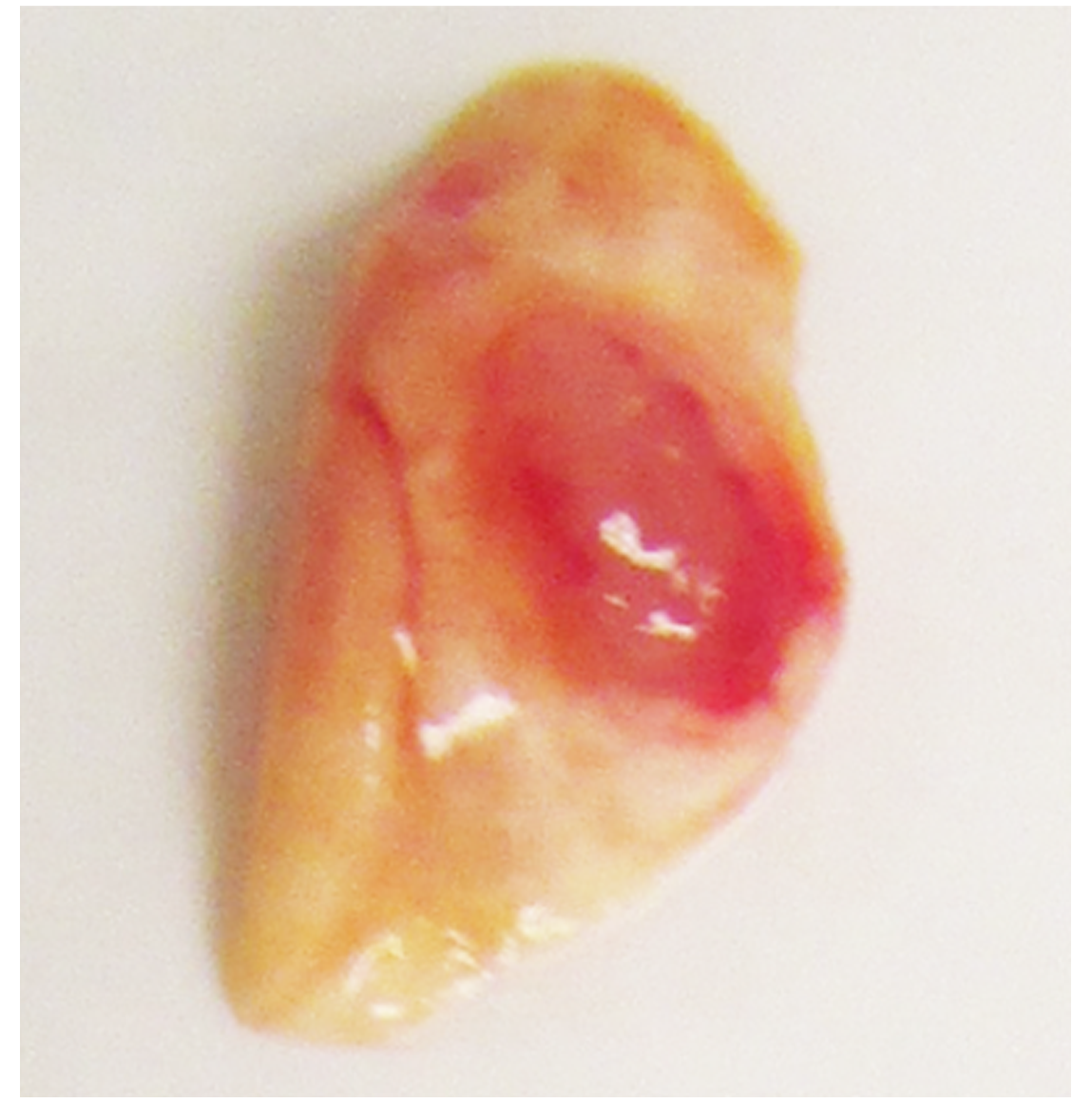

Supplement: Supplementary file 6 — Source data Fig. 5 [file 44321_2024_64_MOESM6_ESM.zip › Figure 5/Figure 5A/Control right.tif]

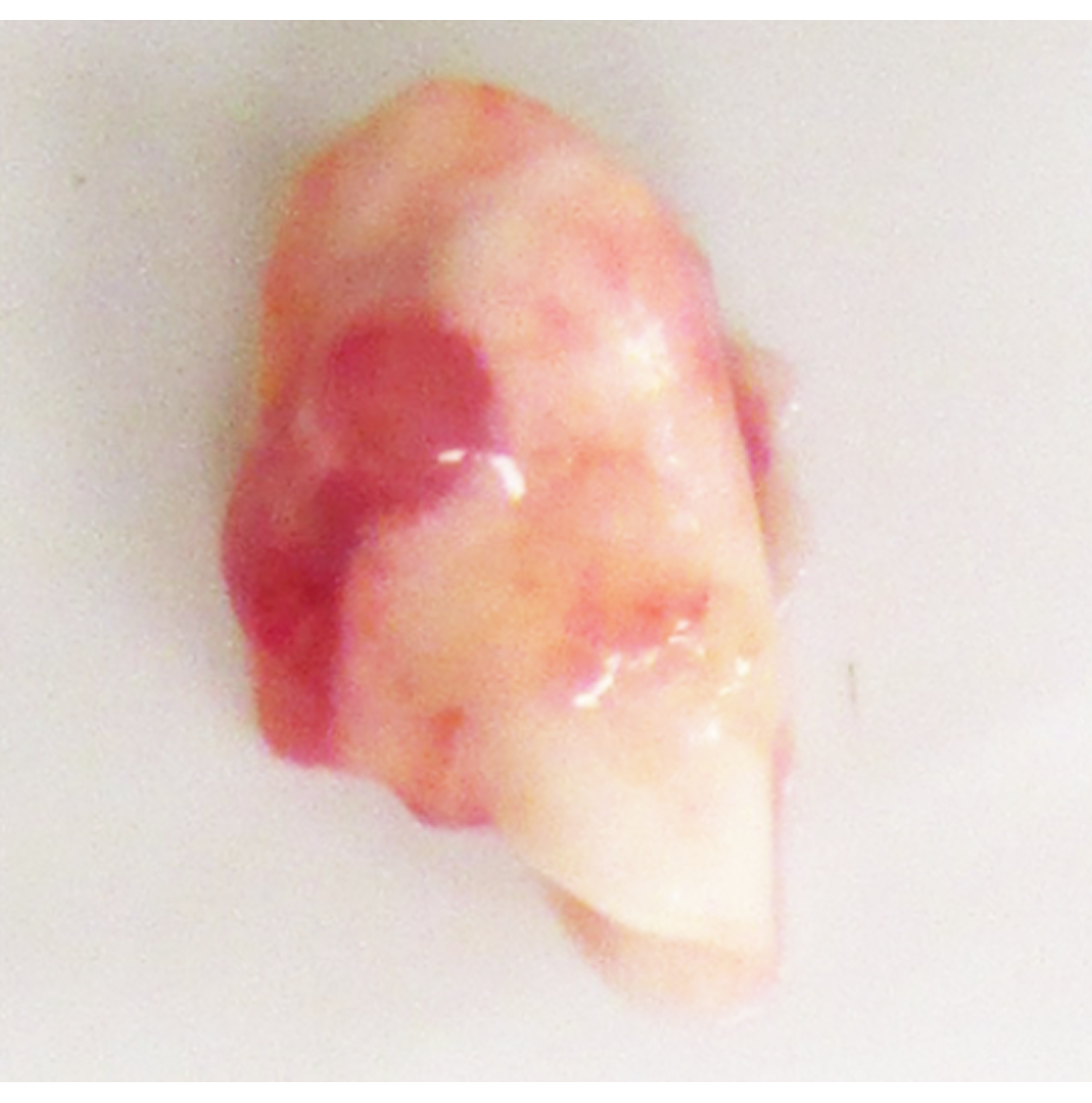

Supplement: Supplementary file 6 — Source data Fig. 5 [file 44321_2024_64_MOESM6_ESM.zip › Figure 5/Figure 5A/endFoxf1OE left.tif]

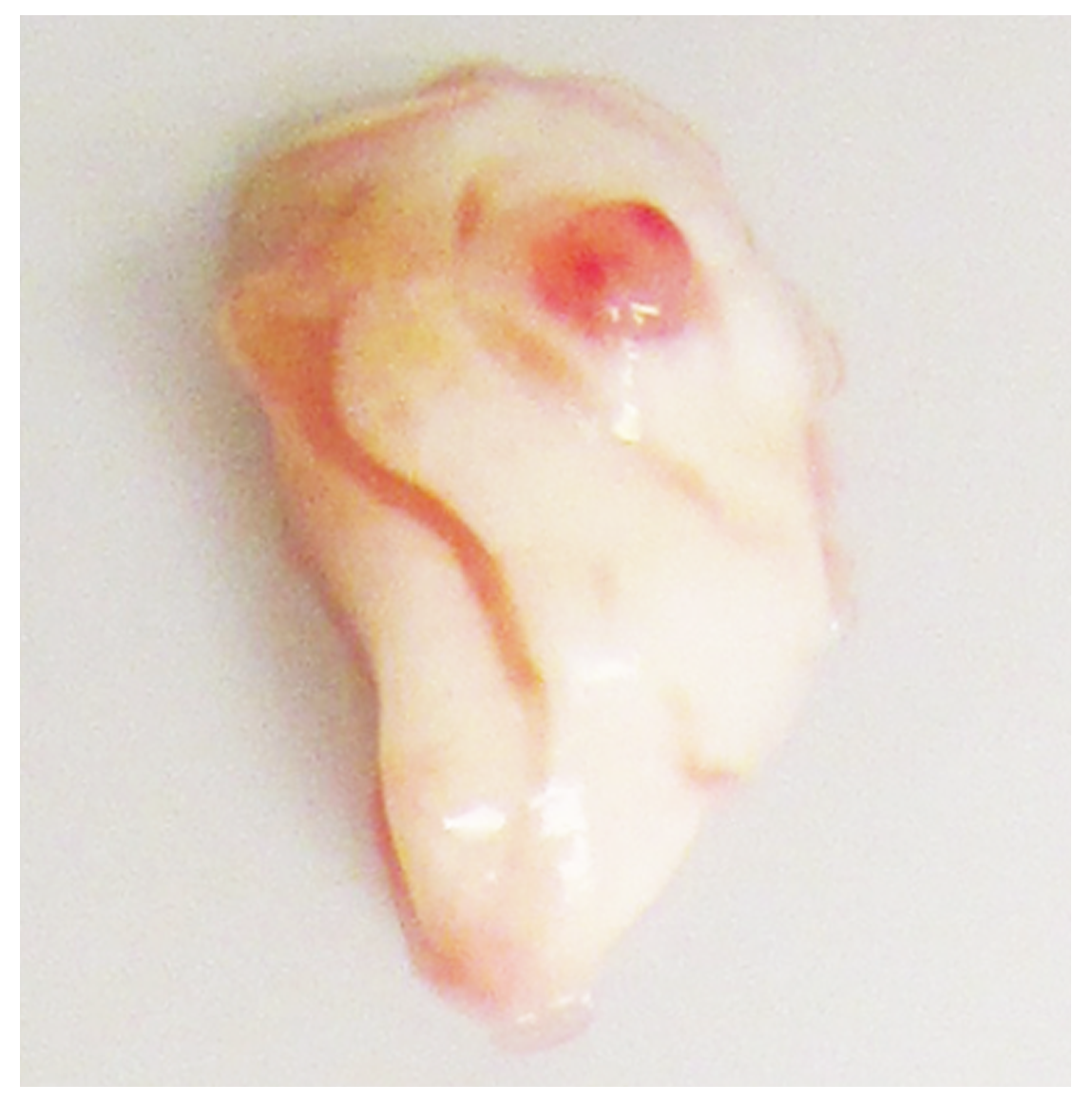

Supplement: Supplementary file 6 — Source data Fig. 5 [file 44321_2024_64_MOESM6_ESM.zip › Figure 5/Figure 5A/endFoxf1OE right.tif]

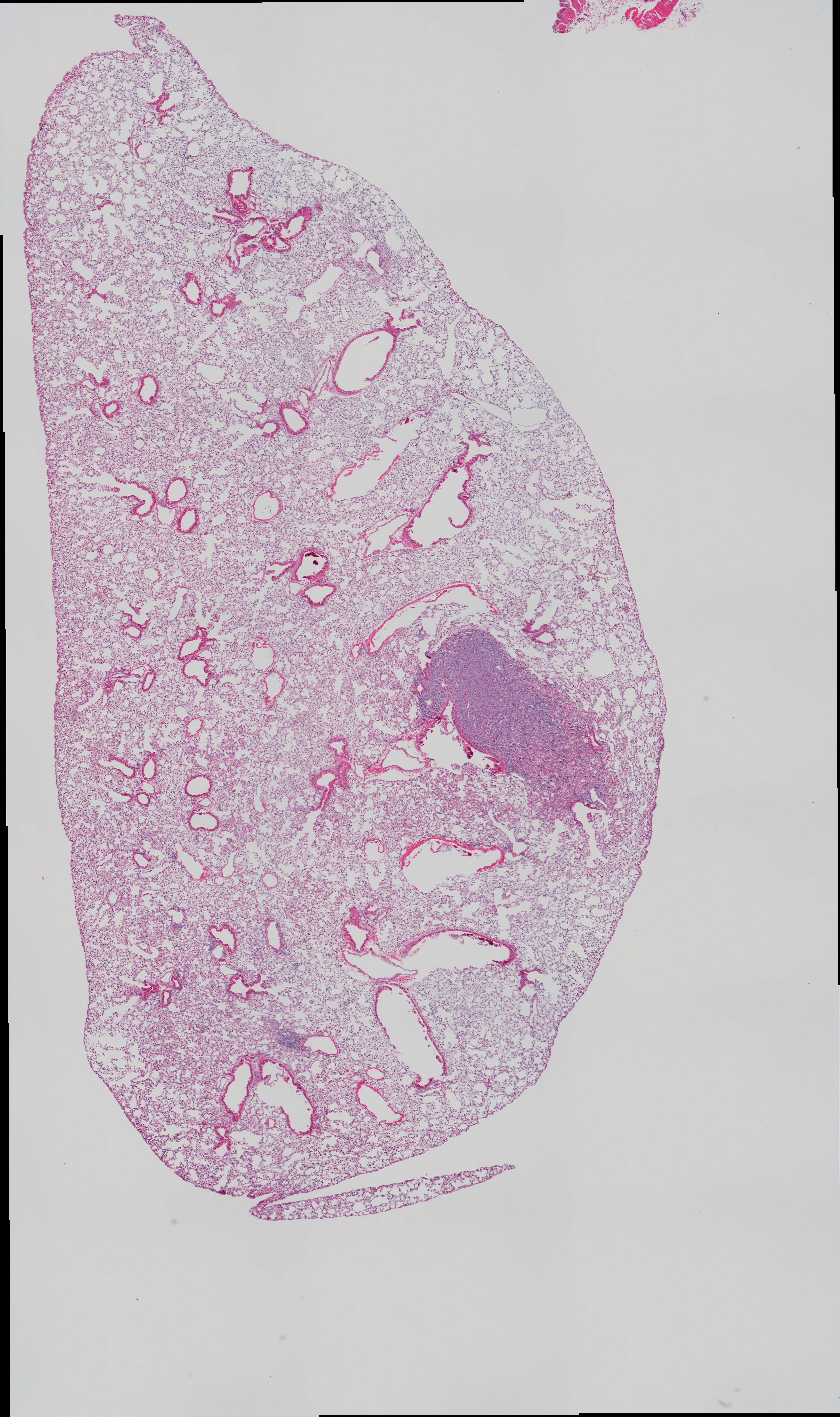

Supplement: Supplementary file 6 — Source data Fig. 5 [file 44321_2024_64_MOESM6_ESM.zip › Figure 5/Figure 5B/endoFoxf1OE.tif]

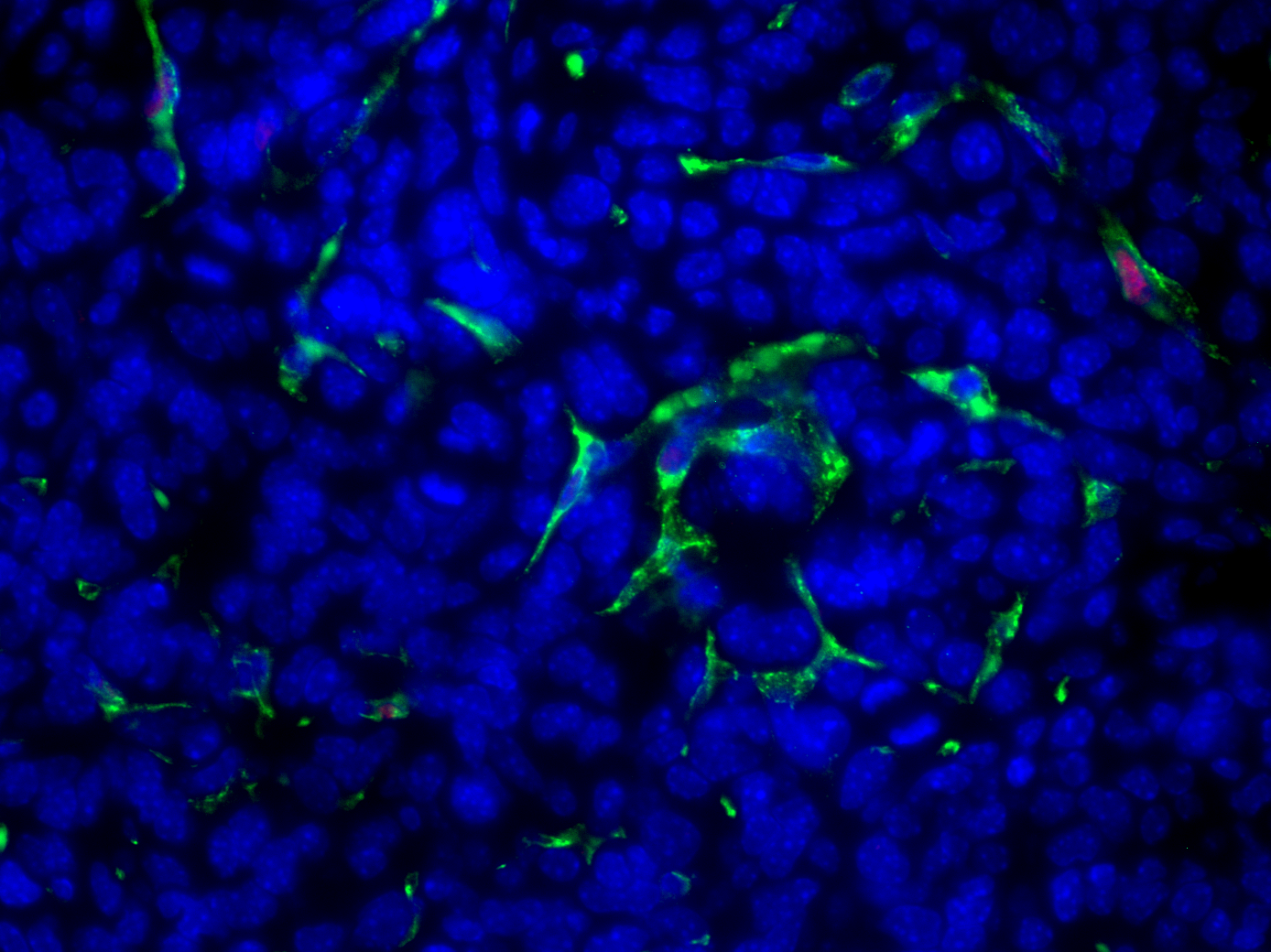

Supplement: Supplementary file 6 — Source data Fig. 5 [file 44321_2024_64_MOESM6_ESM.zip › Figure 5/Figure 5E/Control/#6 con 40X-1_(DAPI+Alexa 488+Alexa 594).TIF]

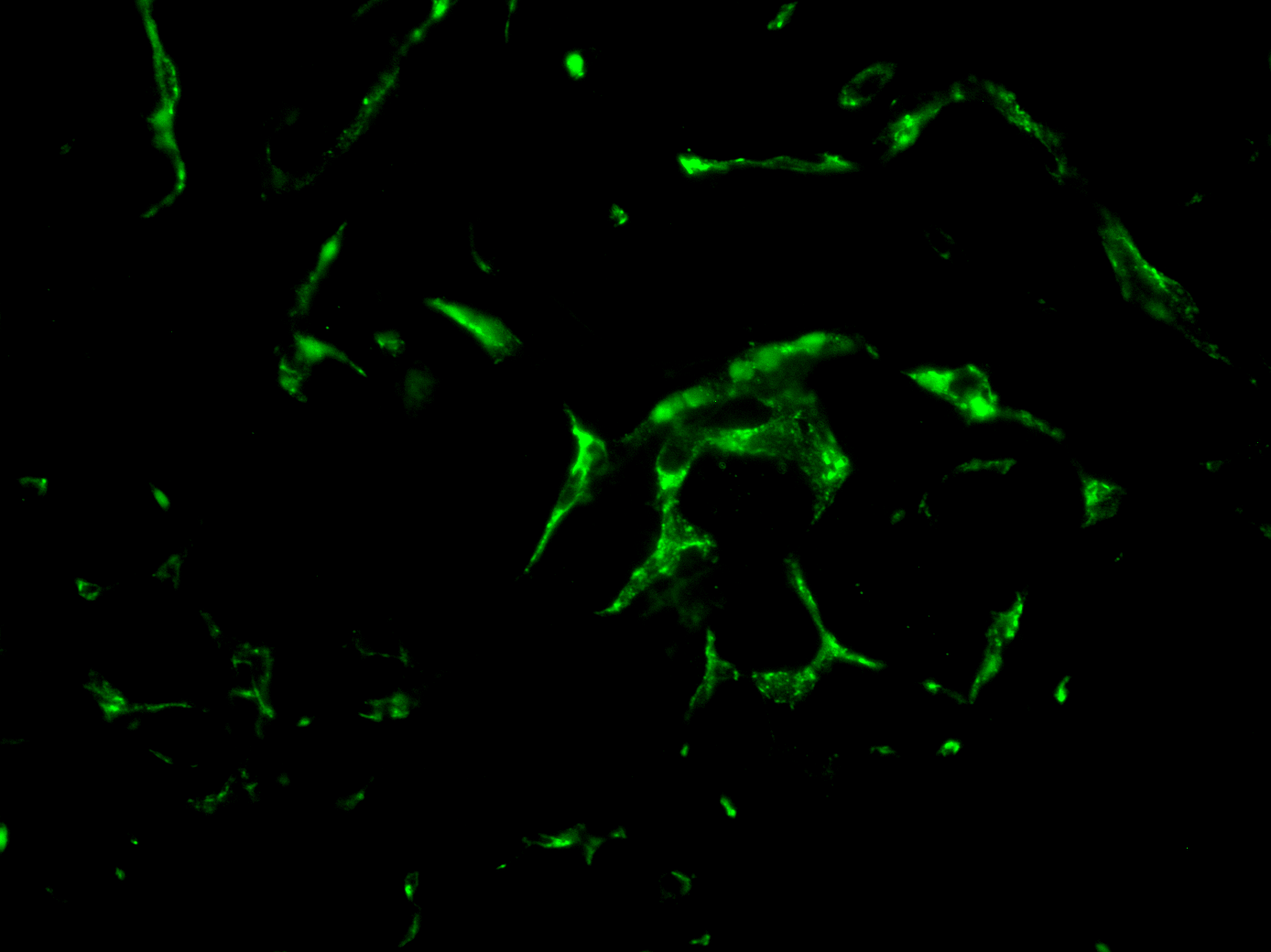

Supplement: Supplementary file 6 — Source data Fig. 5 [file 44321_2024_64_MOESM6_ESM.zip › Figure 5/Figure 5E/Control/#6 con 40X-1_Alexa 488.TIF]

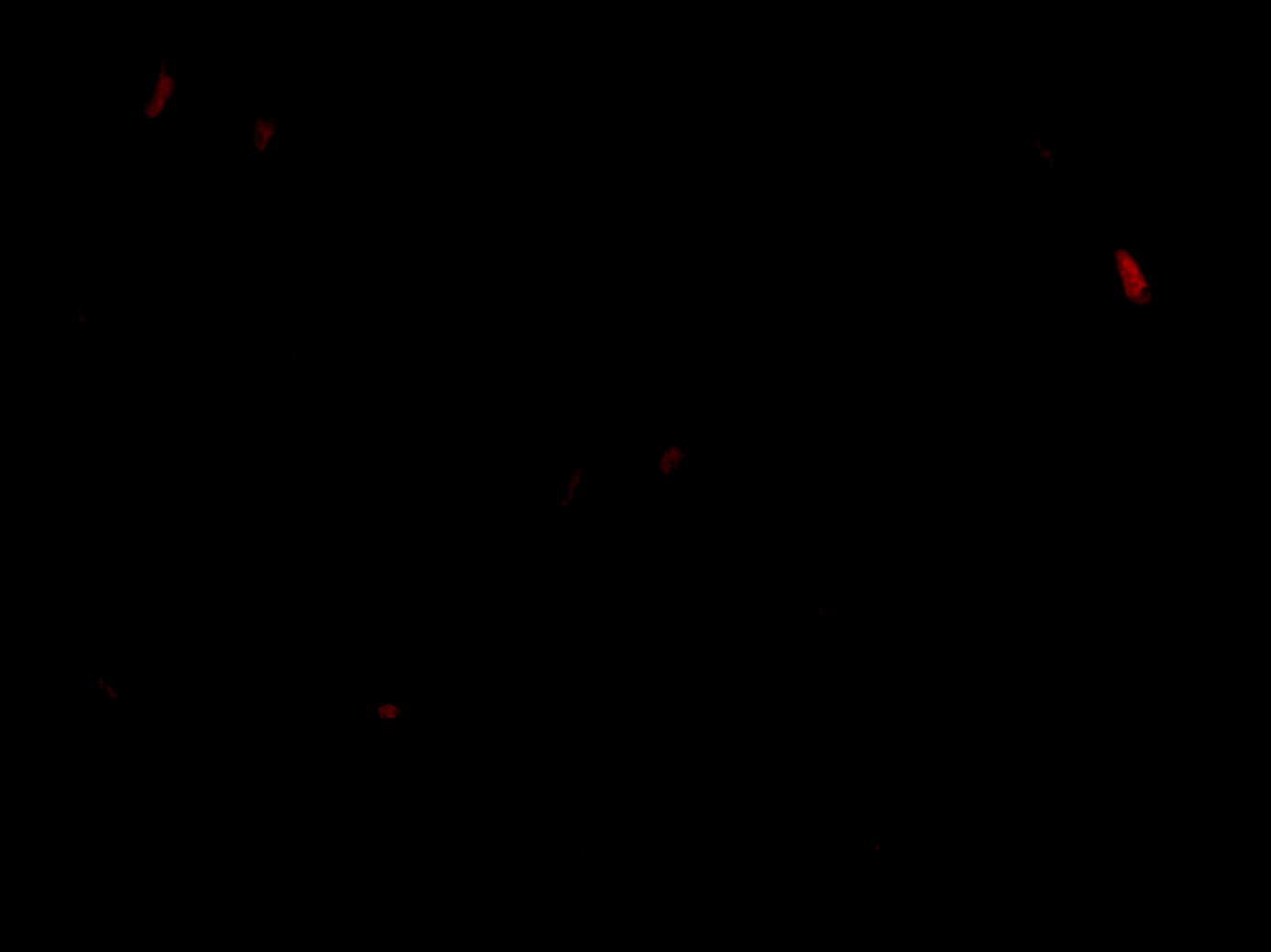

Supplement: Supplementary file 6 — Source data Fig. 5 [file 44321_2024_64_MOESM6_ESM.zip › Figure 5/Figure 5E/Control/#6 con 40X-1_Alexa 594.TIF]

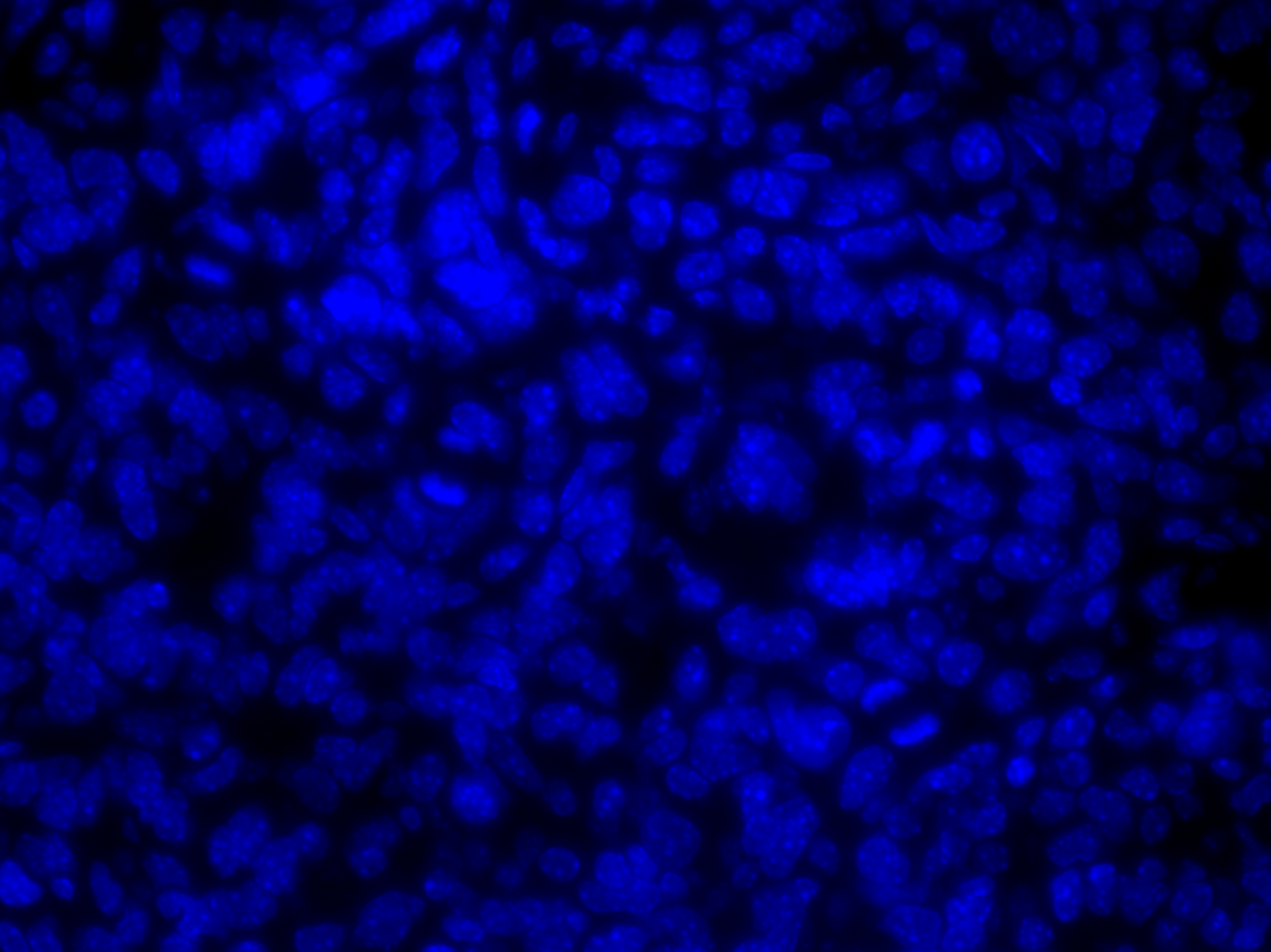

Supplement: Supplementary file 6 — Source data Fig. 5 [file 44321_2024_64_MOESM6_ESM.zip › Figure 5/Figure 5E/Control/#6 con 40X-1_DAPI.TIF]

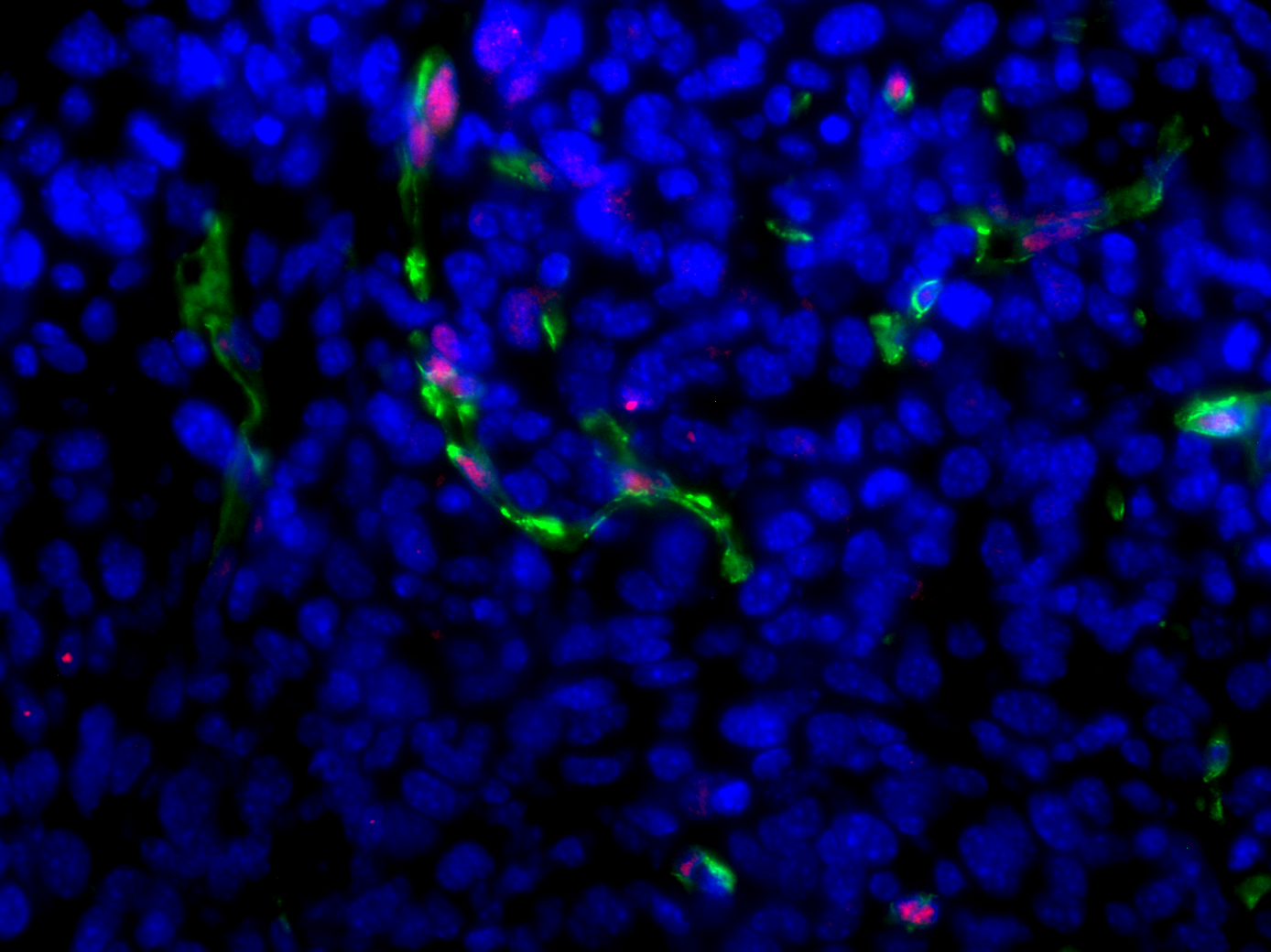

Supplement: Supplementary file 6 — Source data Fig. 5 [file 44321_2024_64_MOESM6_ESM.zip › Figure 5/Figure 5E/endoFoxf1OE/#12 OE 40X-1_(DAPI+Alexa 488+Alexa 594).TIF]

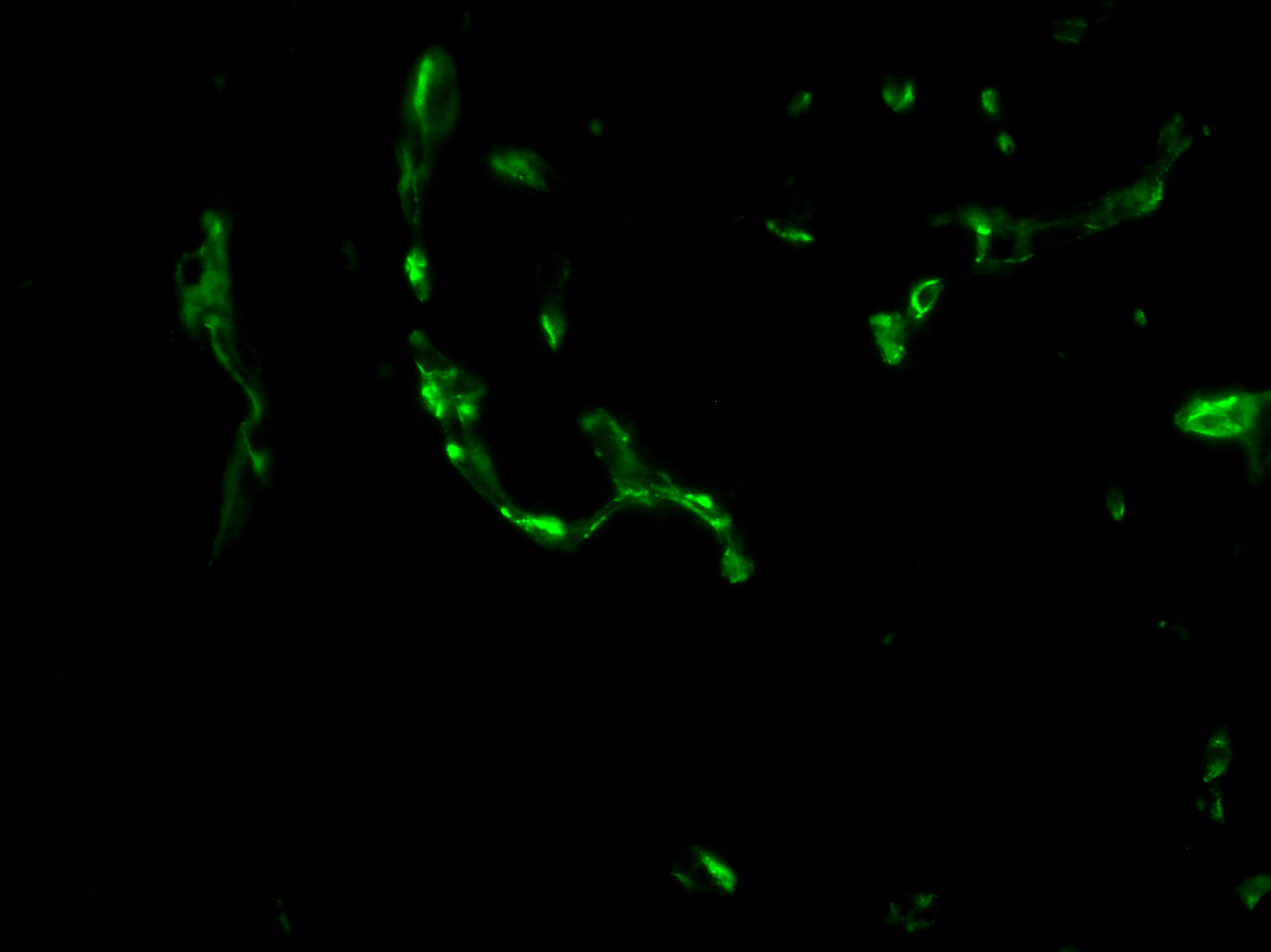

Supplement: Supplementary file 6 — Source data Fig. 5 [file 44321_2024_64_MOESM6_ESM.zip › Figure 5/Figure 5E/endoFoxf1OE/#12 OE 40X-1_Alexa 488.TIF]

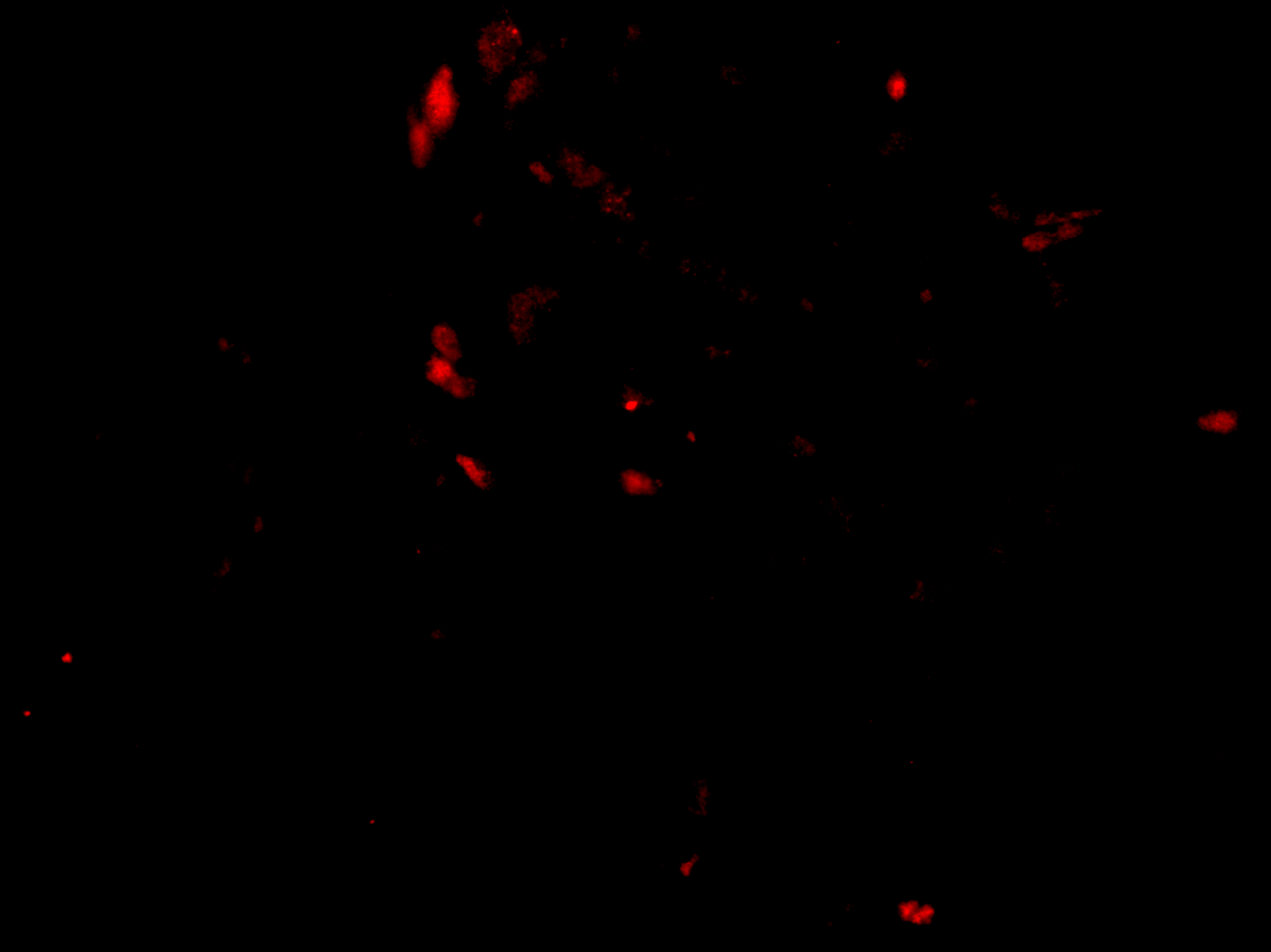

Supplement: Supplementary file 6 — Source data Fig. 5 [file 44321_2024_64_MOESM6_ESM.zip › Figure 5/Figure 5E/endoFoxf1OE/#12 OE 40X-1_Alexa 594.TIF]

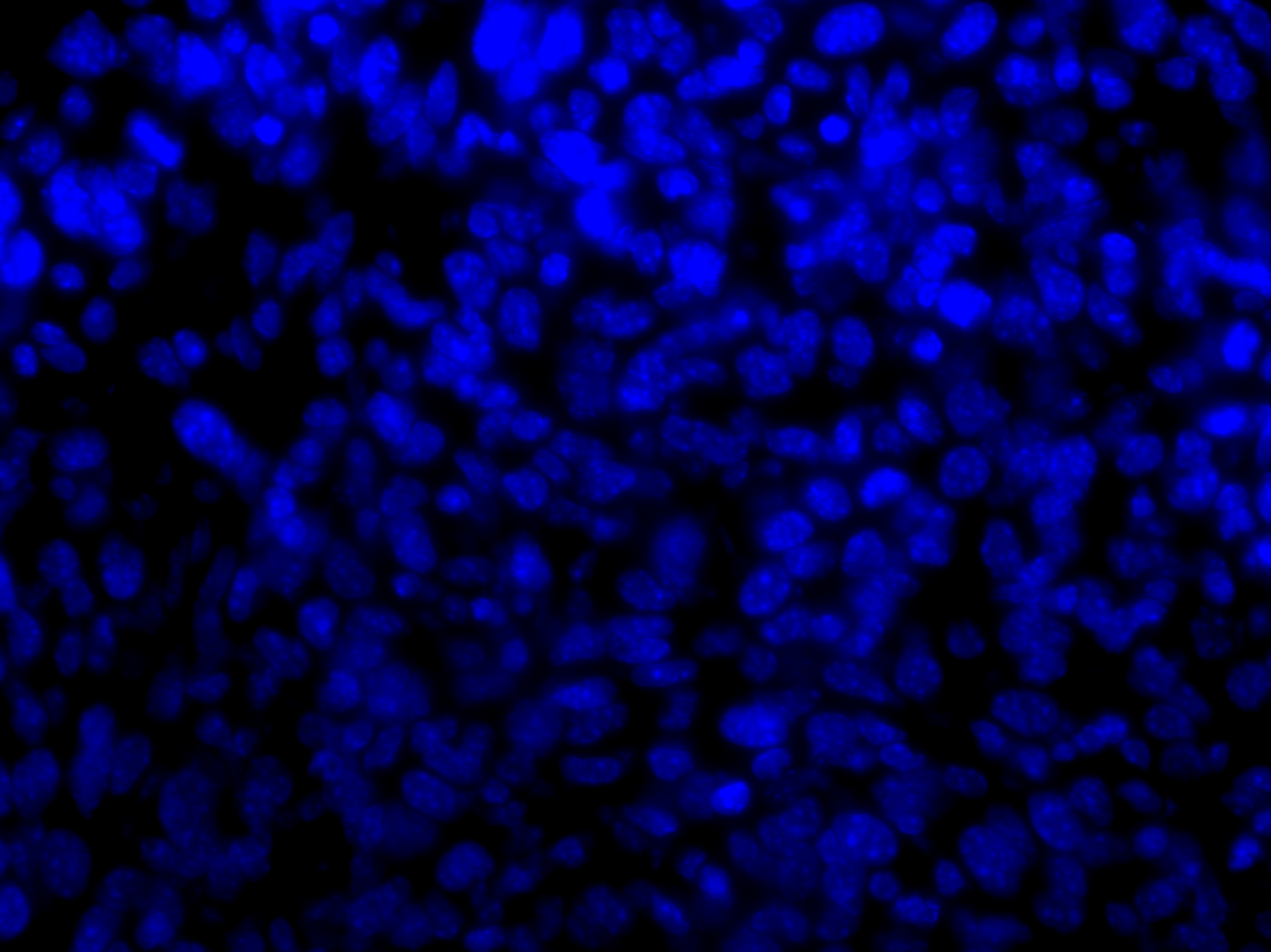

Supplement: Supplementary file 6 — Source data Fig. 5 [file 44321_2024_64_MOESM6_ESM.zip › Figure 5/Figure 5E/endoFoxf1OE/#12 OE 40X-1_DAPI.TIF]

# FZD4 (218665\_at)

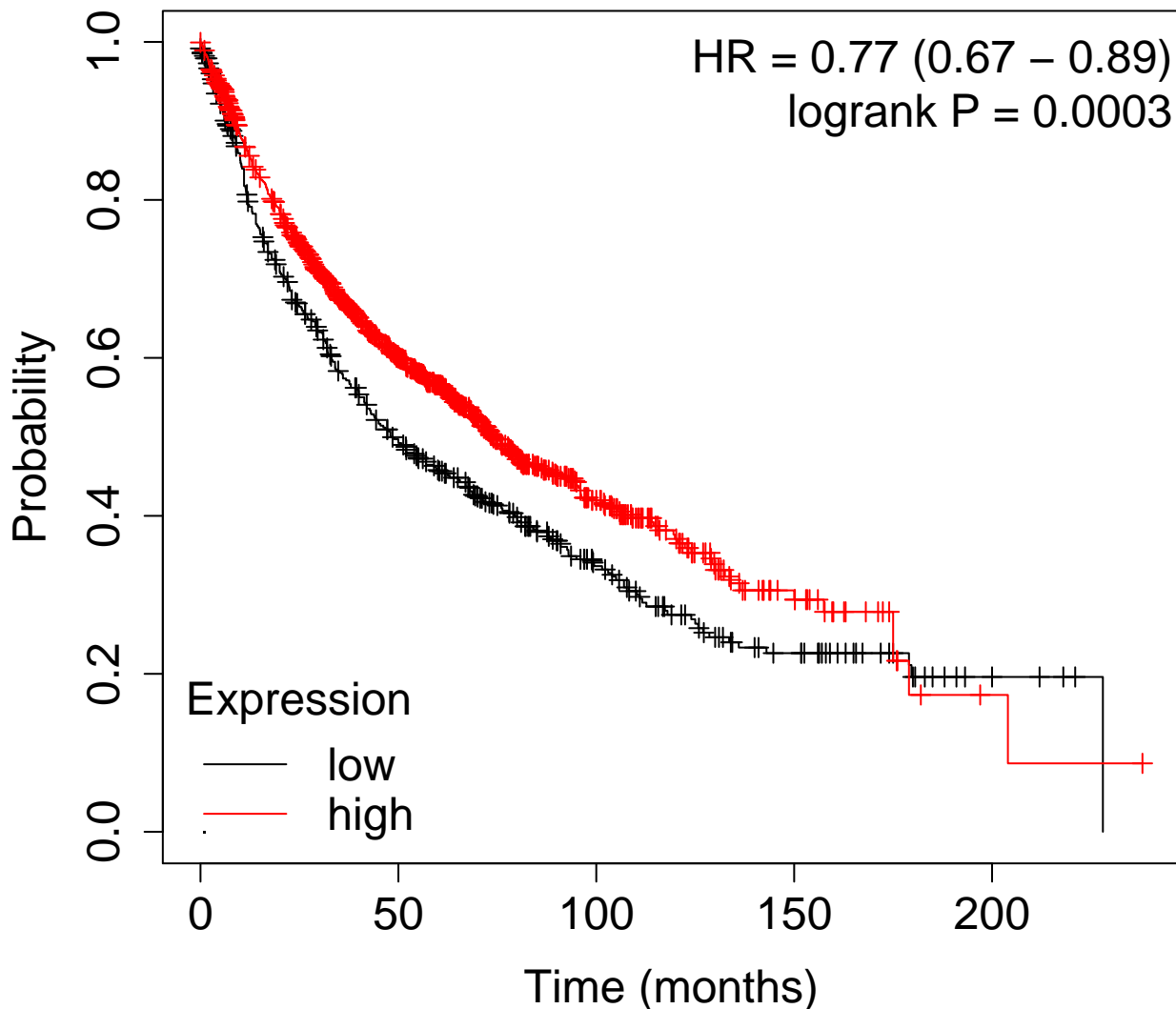

Number at risk

|      |      |     |     |    |   |
|------|------|-----|-----|----|---|
| low  | 483  | 202 | 77  | 31 | 5 |
| high | 1442 | 625 | 126 | 26 | 2 |

Supplement: Supplementary file 7 — Source data Fig. 6 [file 44321_2024_64_MOESM7_ESM.zip › Figure 6/Figure 6E/FZD4 KMPLOT.pdf]

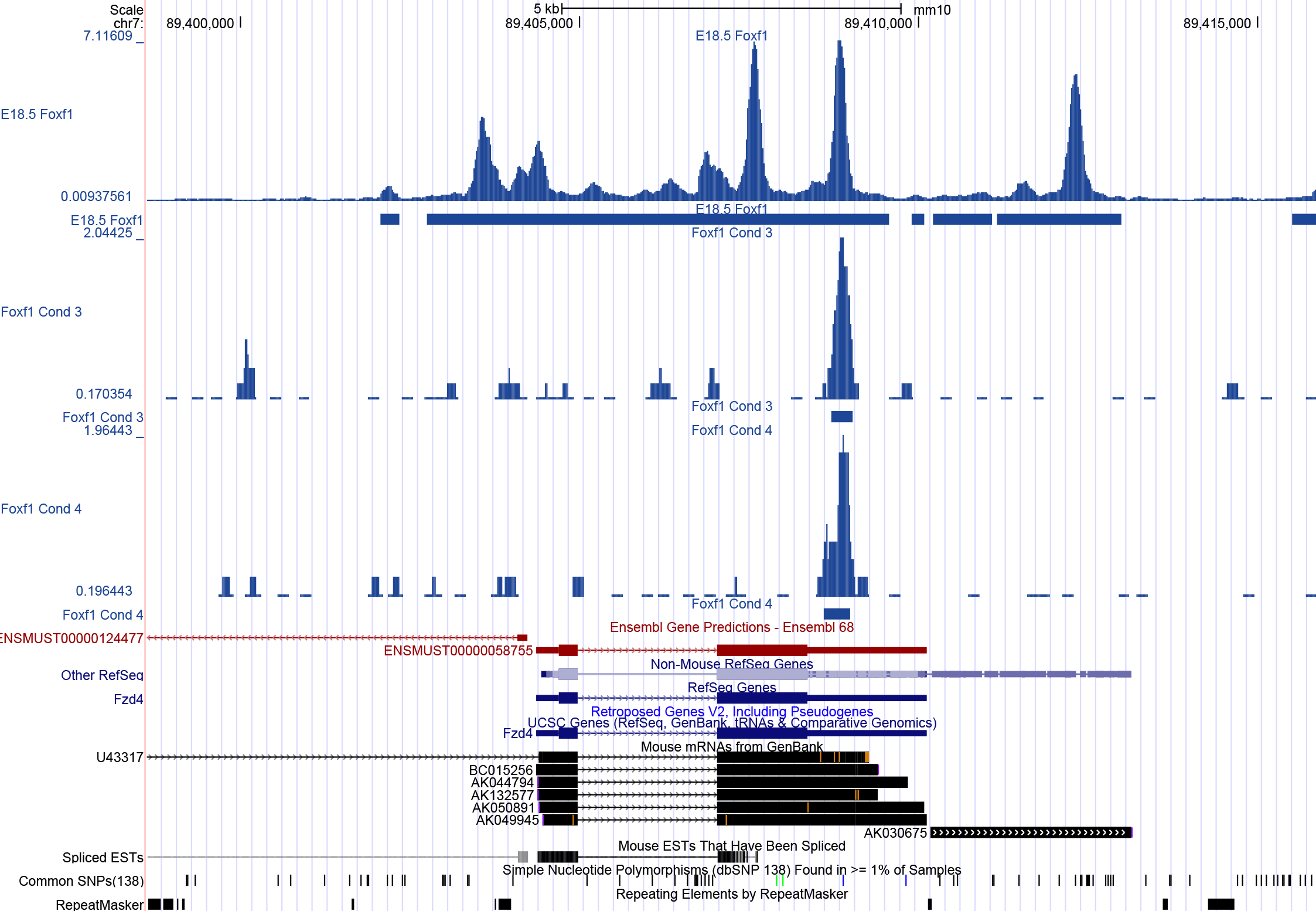

Supplement: Supplementary file 7 — Source data Fig. 6 [file 44321_2024_64_MOESM7_ESM.zip › Figure 6/Figure 6F/foxf1 binding FZD4.tif]

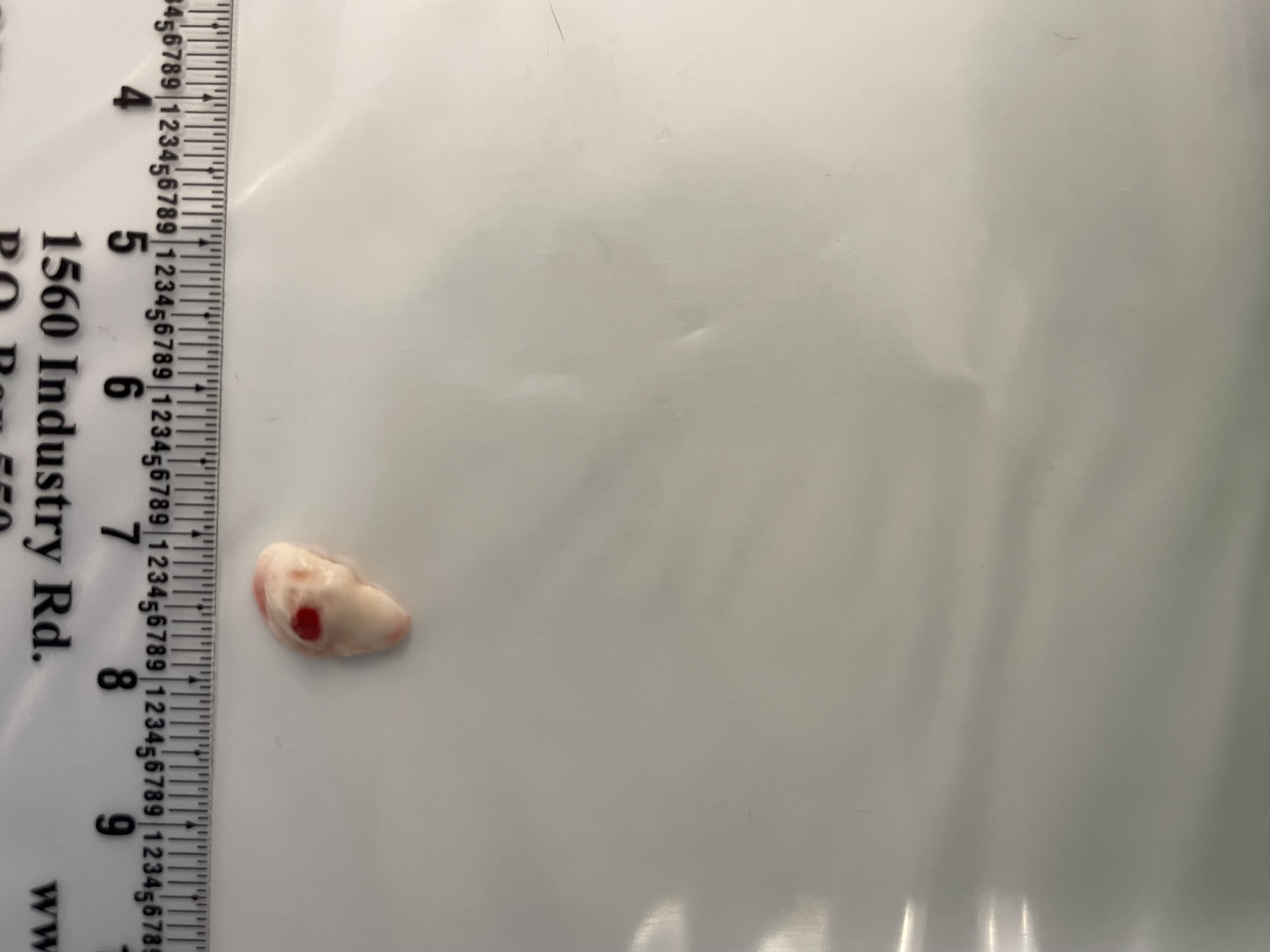

Supplement: Supplementary file 8 — Source data Fig. 7 [file 44321_2024_64_MOESM8_ESM.zip › Figure 7/Figure 7C/Control morpholohy.JPG]

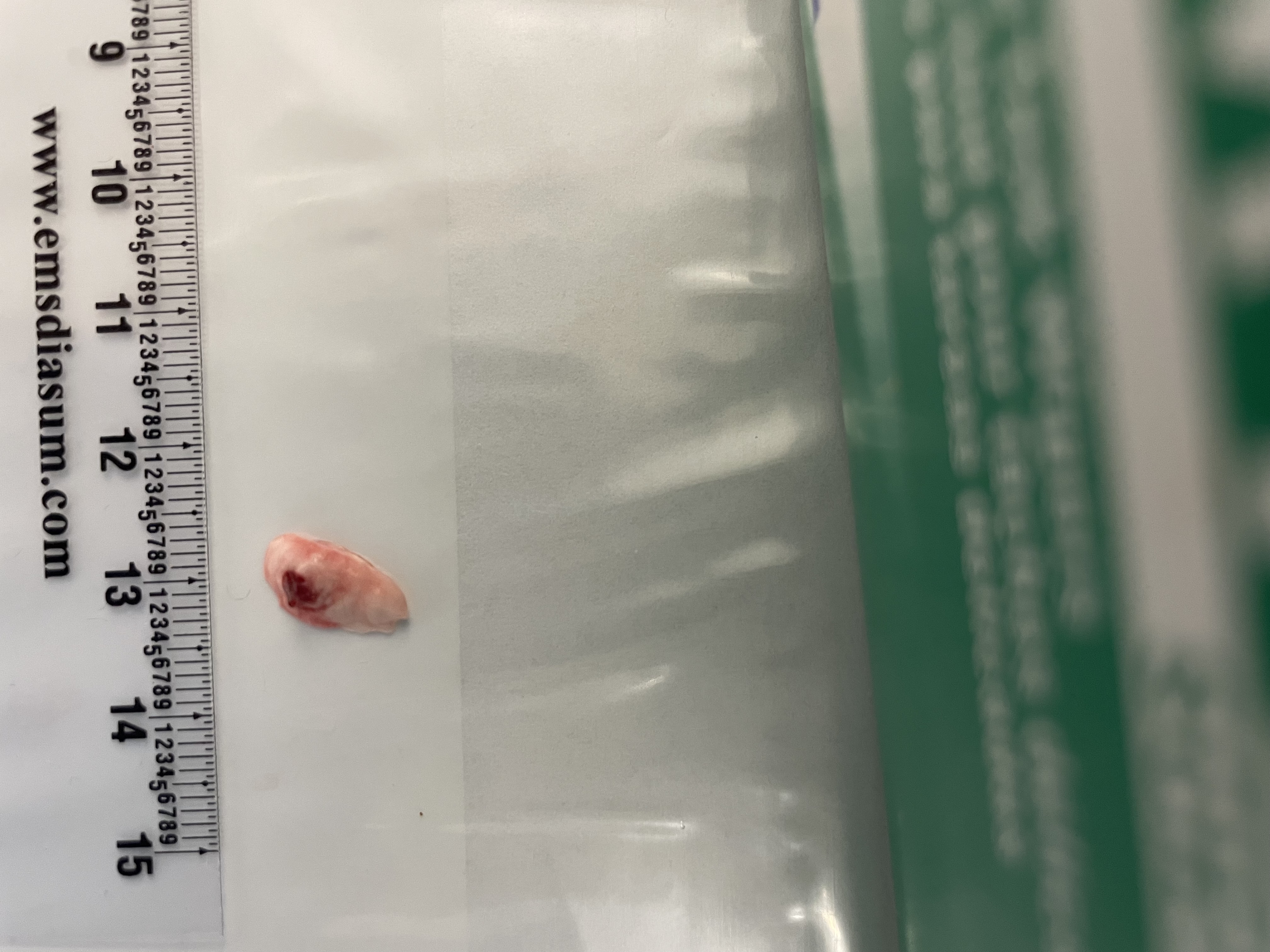

Supplement: Supplementary file 8 — Source data Fig. 7 [file 44321_2024_64_MOESM8_ESM.zip › Figure 7/Figure 7C/endoFoxf1 nano-empty morpholohy.JPG]

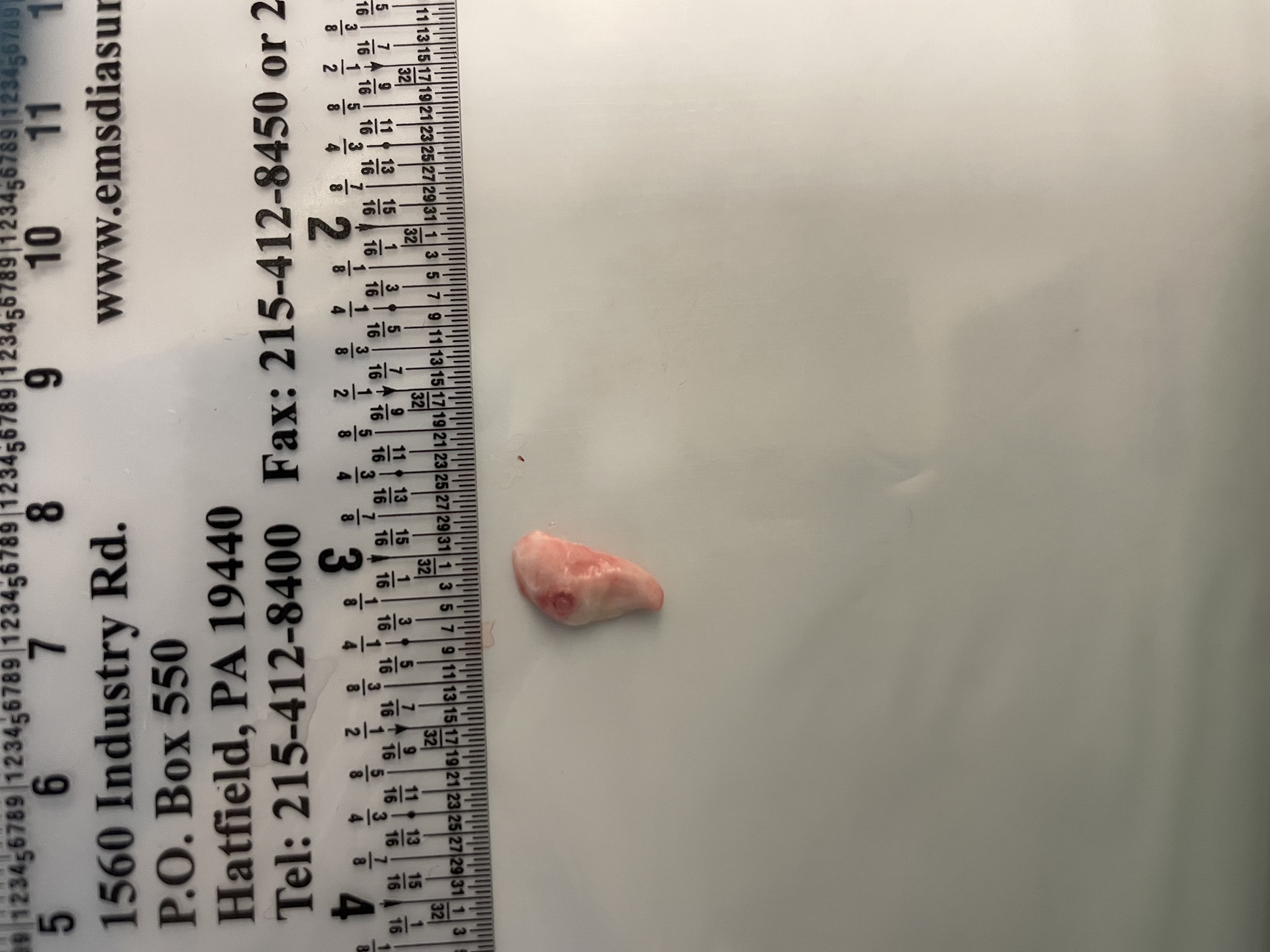

Supplement: Supplementary file 8 — Source data Fig. 7 [file 44321_2024_64_MOESM8_ESM.zip › Figure 7/Figure 7C/endoFoxf1 nano-Fzd4 morpholohy.JPG]
