# Supplementary material for: FOXF1 promotes tumor vessel normalization and prevents lung cancer progression through FZD4
Source: EMBO Mol Med. 2024 Apr 8;16(5):3. doi: 10.1038/s44321-024-00064-8 (PMC11099127; doi:10.1038/s44321-024-00064-8)
Supplement: Supplementary file 11 — Expanded View Figures [file 44321_2024_64_MOESM11_ESM.pdf]

Expanded View Figures

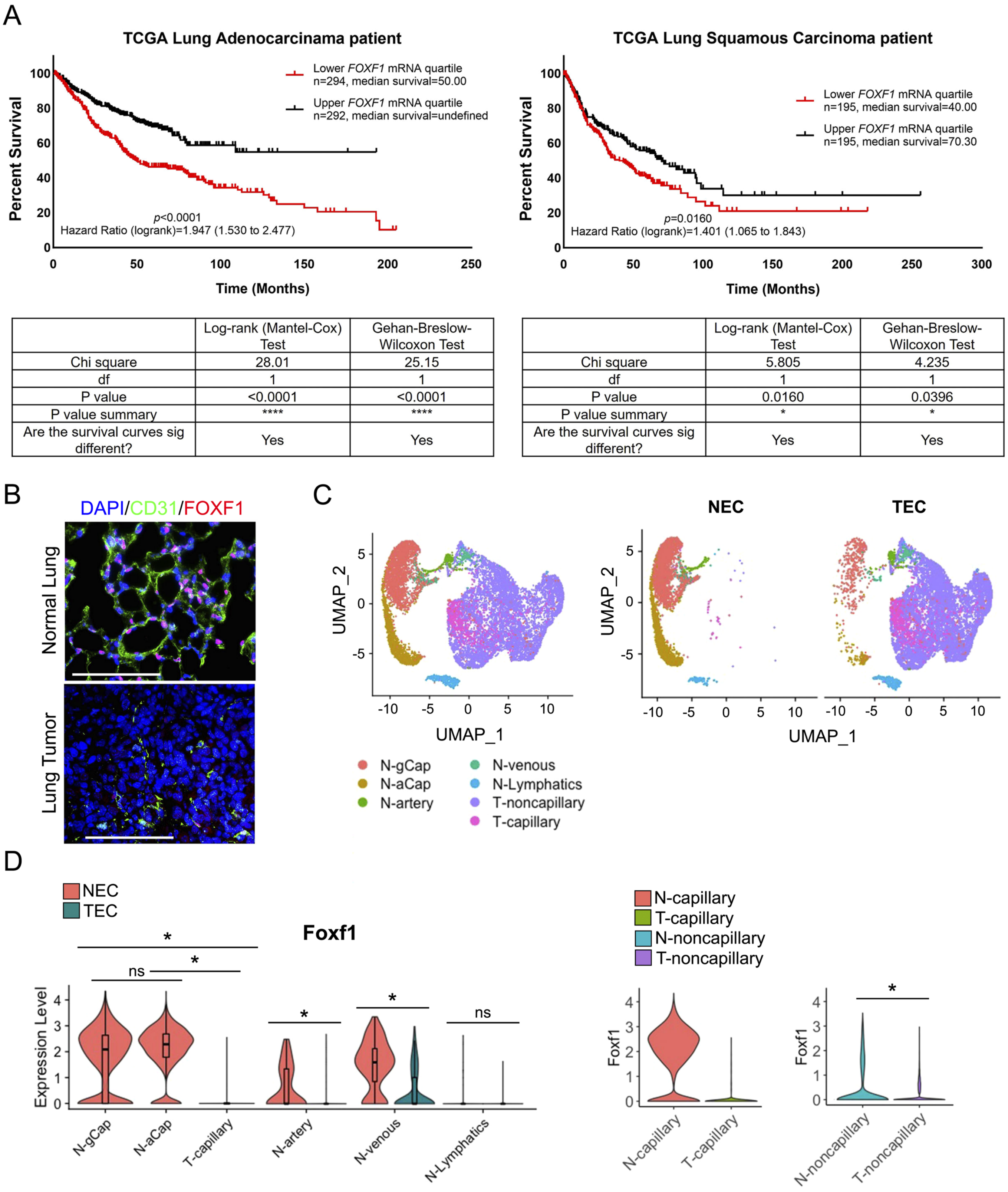

	Log-rank (Mantel-Cox) Test	Gehan-Breslow-Wilcoxon Test
Chi square	28.01	25.15
df	1	1
P value	<0.0001	<0.0001
P value summary	****	****
Are the survival curves sig different?	Yes	Yes

	Log-rank (Mantel-Cox) Test	Gehan-Breslow-Wilcoxon Test
Chi square	5.805	4.235
df	1	1
P value	0.0160	0.0396
P value summary	*	*
Are the survival curves sig different?	Yes	Yes

 © The Author(s)

Figure EV1. ***FOXF1*** **expression is decreased in TECs of mouse LLC lung tumors.**

(**A**) TCGA data mining show that lower *FOXF1* mRNA levels in tumors predicts poor overall survival in patients with adenocarcinoma (AD) and squamous cell carcinoma (SCC). The lower and upper quartile for *FOXF1* expression in AD and SCC were compared. ($N = 1926$). (**B**) Co-localization studies demonstrate decreased FOXF1 protein (red) in CD31$^+$ endothelial cells (green) within mouse LLC tumors compared to normal lungs. The normal lung tissue image is the same as in Fig. 6C. ($N = 3$ mice per group). Scale bar = 100 µm. (**C**) Endothelial cells from normal lung (NEC) and LLC tumors (TEC) were visualized using uniform manifold approximation and projection (UMAP) after samples integration with Harmony. (**D**) Both *Foxf1* mRNA levels and the total number of FoxF1-expressing EC were decreased in LLC lung tumors compared to normal lung. Based on endothelial cell sub-clustering, decreased Foxf1 mRNA was detected in tumor-associated capillary, arterial and venous ECs. Foxf1 was not expressed in lymphatics. Colored by group; red- Donor EC, (NEC; $n = 3841$ cells); blue- EC from LLC tumors, (TEC, $n = 10012$ cells). Boxes show median, Q1 and Q3 quartiles and whiskers up to 1.5× interquartile range. Data information: Data represent different numbers (N) of biological replicates. The data with error bars are shown as mean ± SEM. *$P < 0.05$, ns: not significant, as determined using the logrank test (**A**), or Wilcoxon rank-sum test (**D**).

© The Author(s)

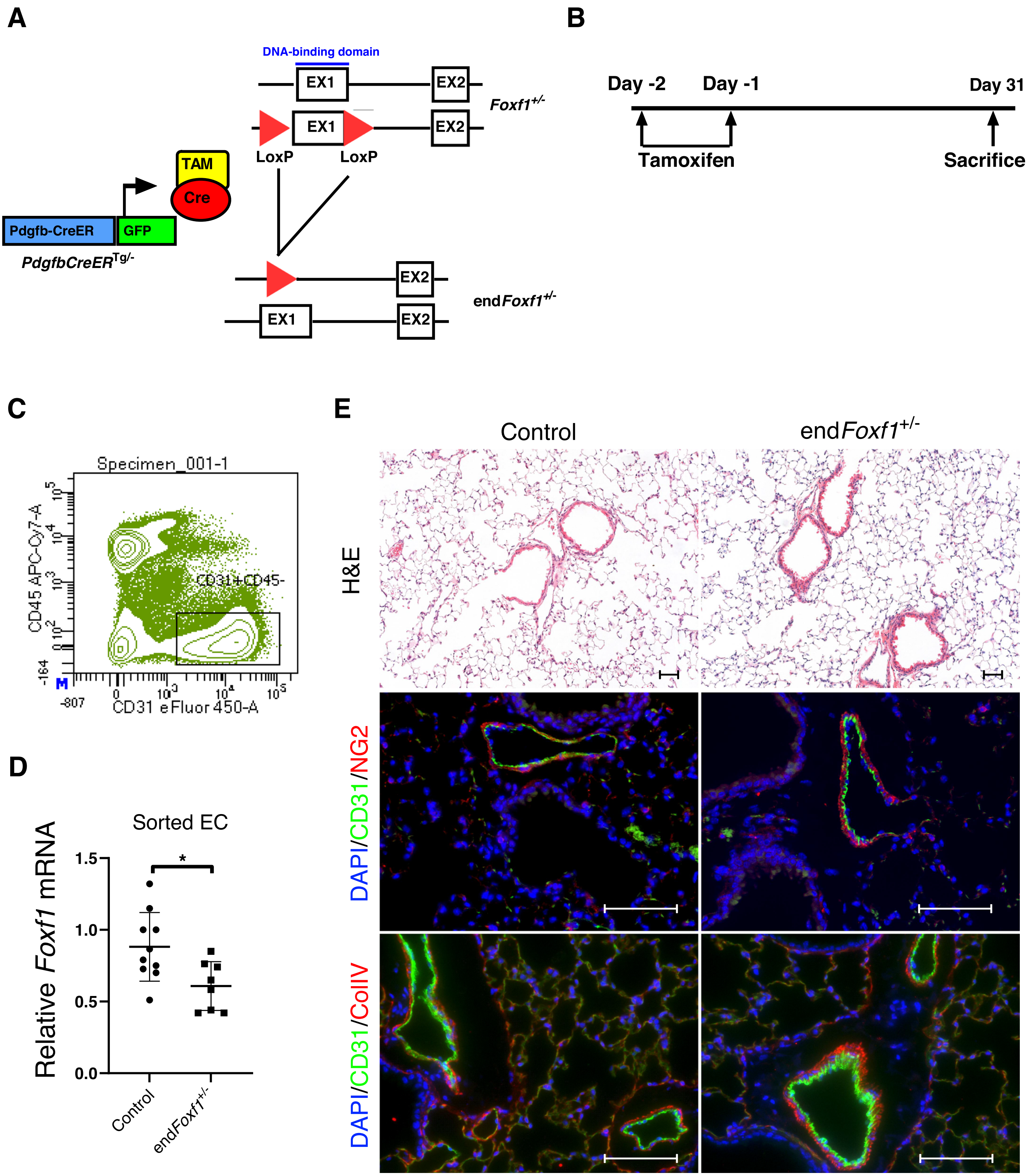
A
DNA-binding domain
EX1
EX2
Foxf1+/-
LoxP
LoxP
TAM
Cre
Pdgfb-CreER
GFP
PdgfbCreERTg/-
endFoxf1+/-
B
Day -2
Day -1
Day 31
Tamoxifen
Sacrifice
C
Specimen_001-1
CD45 APC-Cy7-A
CD31+CD45-
CD31 eFluor 450-A
D
Sorted EC
Relative Foxf1 mRNA
1.5
1.0
0.5
0.0
*
Control
endFoxf1+/-
E
Control
endFoxf1+/-
H&E
DAPI/CD31/NG2
DAPI/CD31/ColIV

 © The Author(s)

Figure EV2. Conditional deletion of one *Foxf1* allele from endothelial cells does not affect lung histology.

(**A**) Schematic representation of breeding strategy for the conditional deletion of one allele of *Foxf1* from endothelial cells (end*Foxf1*$^{+/-}$ mice). *Foxf1*$^{F/F}$ mice were crossed with *Pdgfb-cre*ER$^{+/-}$ mice to generate *Pdgfb-creER*$^{+/-}$; *Foxf1*$^{F/+}$ mice. *Foxf1*$^{F/+}$ littermates were used as controls. (**B**) Schematic representation of tamoxifen treatment to delete *Foxf1* from endothelial cells. (**C**) The gating strategy for FACS-sorting. Single, live cells from the lungs were gated to identify endothelial cells ($CD45^-$ $CD31^+$). (**D**) qRT-PCR shows decreased *Foxf1* mRNA in endothelial cells isolated from end*Foxf1*$^{+/-}$ lungs compared to control lungs. *Actb* mRNA was used for normalization. ($N = 8$–10 mice per group). **P* = 0.0151. (**E**) H&E staining (upper panels) shows no morphological differences between tamoxifen-treated control *Foxf1*$^{F/+}$ lungs and end*Foxf1*$^{+/-}$ lungs at 31 days after tamoxifen administration. Scale bar = 25 μm. No difference in pericyte coverage of blood vessels was found in control and end*Foxf1*$^{+/-}$ lungs is shown by co-localization of CD31 (green) with NG2 (red) (Middle panels). Blood vessels in end*Foxf1*$^{+/-}$ and control lungs have similar basement membrane as shown by co-localization of CD31 (green) with Collagen IV (red). (Bottom panels). Scale bar = 100 μm. Data information: Data represent different numbers (*N*) of biological replicates. The data with error bars are shown as mean ± SD. Statistical analysis was performed using Mann–Whitney Two-tailed test (**D**). Source data are available online for this figure.

© The Author(s)

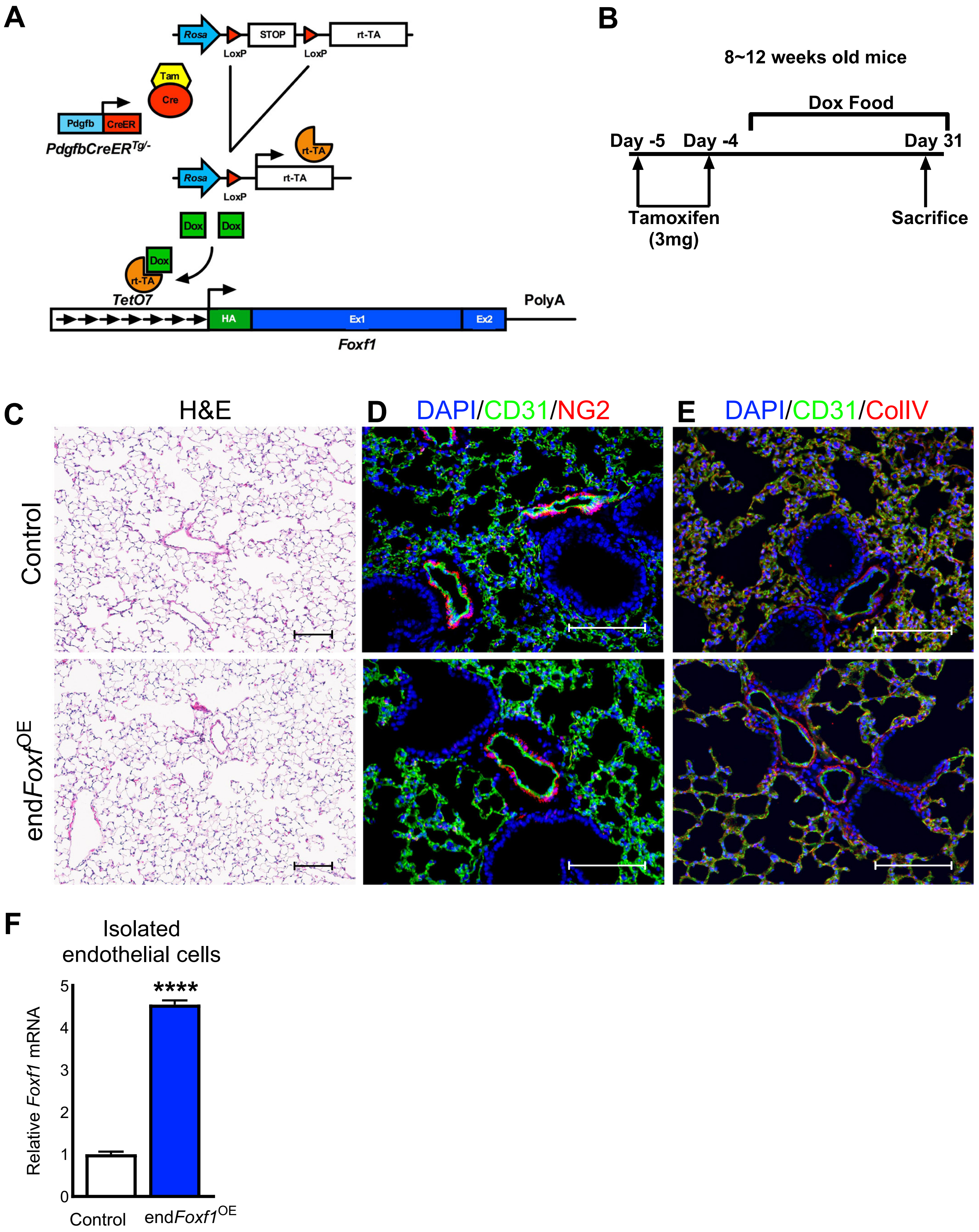
A
Rosa
LoxP
STOP
LoxP
rt-TA
Tam
Cre
Pdgfb
CreER
PdgfbCreER^Tg/-
rt-TA
Rosa
LoxP
rt-TA
Dox
Dox
Dox
rt-TA
TetO7
PolyA
HA
Ex1
Ex2
Foxf1
B
8~12 weeks old mice
Dox Food
Day -5
Day -4
Day 31
Tamoxifen
(3mg)
Sacrifice
C
H&E
D
DAPI/CD31/NG2
E
DAPI/CD31/CollV
Control
endFoxf1^OE
F
Isolated
endothelial cells
Relative Foxf1 mRNA
5
4
3
2
1
0
****
Control
endFoxf1^OE

Figure EV3. Overexpression of FOXF1 in endothelial cells does not affect lung architecture.

(**A**) Schematic representation shows breeding strategy for overexpression of FOXF1 in endothelial cells (end*Foxf1*^{OE}). (**B**) Schematic representation of tamoxifen and doxycycline treatment to overexpress FOXF1 in endothelial cells. (**C**) H&E staining shows no morphological differences between tamoxifen+doxycycline-treated control and end*Foxf1*^{OE} mouse lungs at 31 days after the treatment. Scale bar = 50 μm. (**D**) No difference in pericyte coverage of blood vessels in control and end*Foxf1*^{OE} lungs is shown by co-localization of CD31 (green) with NG2 (red). (**E**) Blood vessels in end*Foxf1*^{OE} and control lungs have similar basement membrane as shown by co-localization of CD31 (green) with Collagen IV (red). (**F**) *Foxf1* mRNA is increased by ~4.5-fold in $CD45^{-}CD31^{+}$ endothelial cells FACS-sorted from tamoxifen+doxycycline-treated end*Foxf1*^{OE} mice compared to controls. β-*actin* mRNA was used for normalization. ($N = 3$ mice per group). Scale bar = 100 μm. ****$P < 0.0001$. Data information: Data represent different numbers (N) of biological replicates. The data with error bars are shown as mean ± SEM. Statistical analysis was performed using the two-tailed unpaired-sample Student t test (**F**). Source data are available online for this figure.

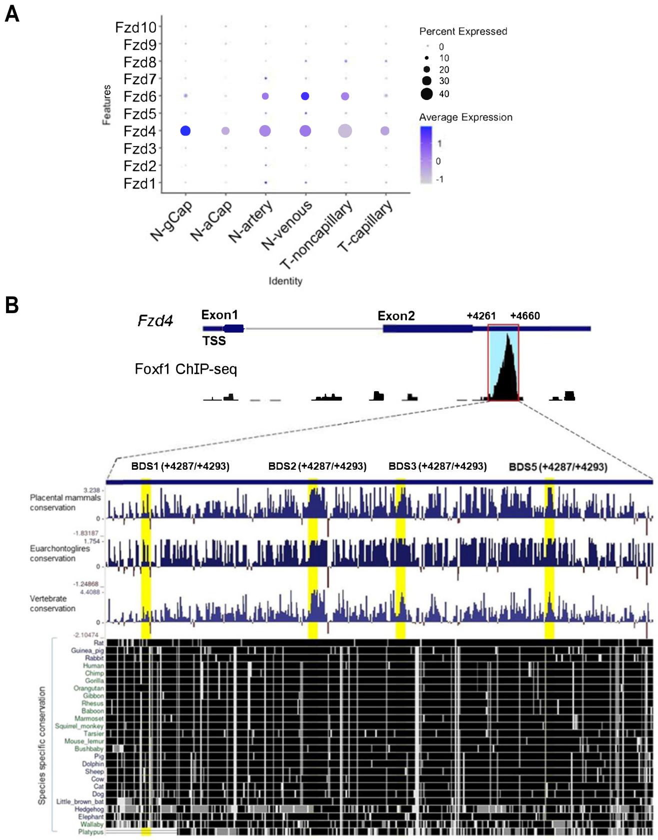

Figure EV4. Fzd4 is a direct transcriptional target of FOXF1.

(**A**) Expression of Frizzled receptor mRNAs in lung endothelial cells based on scRNA-seq analysis. (**B**) Analysis of ChIP-seq dataset from MFLM-91U endothelial cells shows FOXF1-binding picks in Fzd4 gene region located 3′ to the exon 2 of the Fzd4 coding sequence (top panel). Homology of the Fzd4 gene with FOXF1-binding sites (BDS) is shown for several mammalian species (bottom panel).

 © The Author(s)

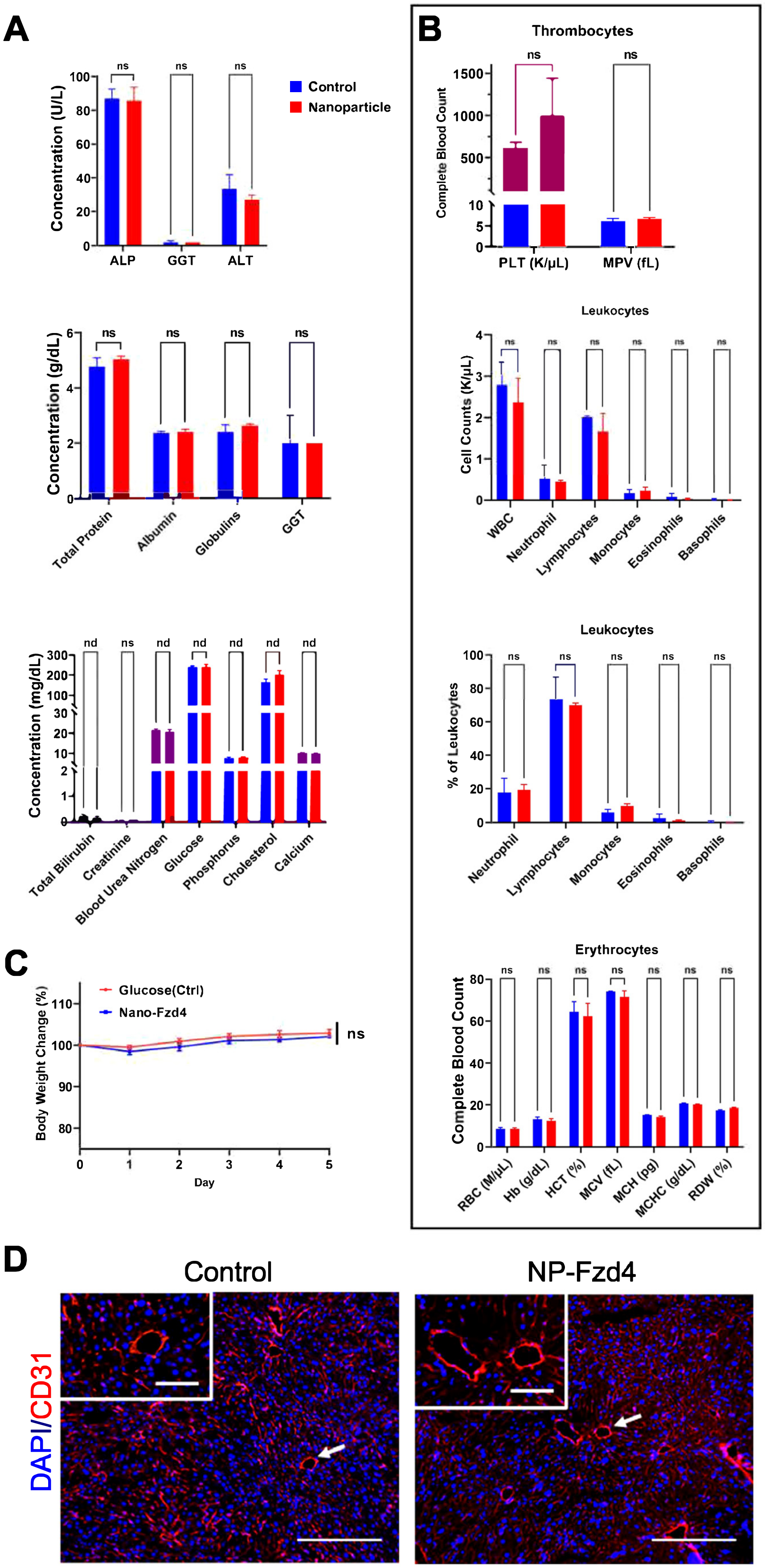
A
Control
Nanoparticle
Concentration (U/L)
ALP
GGT
ALT
ns
Concentration (g/dL)
Total Protein
Albumin
Globulins
GGT
Concentration (mg/dL)
Total Bilirubin
Creatinine
Blood Urea Nitrogen
Glucose
Phosphorus
Cholesterol
Calcium
nd
B
Thrombocytes
Complete Blood Count
PLT (K/μL)
MPV (fL)
Leukocytes
Cell Counts (K/μL)
WBC
Neutrophil
Lymphocytes
Monocytes
Eosinophils
Basophils
% of Leukocytes
Erythrocytes
RBC (M/μL)
Hb (g/dL)
HCT (%)
MCV (fL)
MCH (pg)
MCHC (g/dL)
RDW (%)
C
Glucose(Ctrl)
Nano-Fzd4
Body Weight Change (%)
Day
D
Control
NP-Fzd4
DAPI/CD31

◀ **Figure EV5. Treatment with intravenously injected PBAE nanoparticles carrying CMV- *Fzd4* (nano-Fzd4) is not toxic.**

(**A**) Normal liver functions: No differences were found in the concentrations of total protein, Albumin, Globulins, ALP, Total Bilirubin, GGT, and ALT in peripheral blood serum of mice treated with nano-Fzd4 compared to control mice treated with glucose. $N = 5$ mice per group. (**B**) Normal kidney functions: No differences in BUN and Creatinine levels were found in the nano-Fzd4 treated group compared to the control group. $N = 5$ mice per group. (**C**) No changes in hematologic parameters compared to the control group. $N = 5$ mice per group. (**D**) Nano-Fzd4 treatment does not change the histological appearance of endothelial cells in the liver shown with immunostaining for Pecam1 (CD31). Arrows indicated blood vessels. $N = 2–3$ mice per group. Data information: Data represent different numbers (N) of biological replicates. The data with error bars are shown as mean ± SEM. ns: not significant, as determined using two-tailed unpaired-sample Student *t* test (**A**–**C**). Source data are available online for this figure.

© The Author(s)
